# Factors Associated with Participation of Community-Dwelling Older Adults in a Home-Based Falls Prevention Program

**DOI:** 10.3390/ijerph16061087

**Published:** 2019-03-26

**Authors:** Branko F. Olij, Lotte M. Barmentloo, Dini Smilde, Nathalie van der Velde, Suzanne Polinder, Yvonne Schoon, Vicki Erasmus

**Affiliations:** 1 Erasmus MC, Department of Public Health, University Medical Center Rotterdam, 3000 CA Rotterdam, The Netherlands; l.barmentloo@erasmusmc.nl (L.M.B.); s.polinder@erasmusmc.nl (S.P.); v.erasmus@erasmusmc.nl (V.E.); 2 GENERO Foundation, 3001 AE Rotterdam, The Netherlands; dsmilde@xs4all.nl; 3 Amsterdam UMC, Department of Internal Medicine, University of Amsterdam, Section of Geriatric Medicine, Amsterdam Public Health Research Institute, 1105 AZ Amsterdam, The Netherlands; n.vandervelde@amc.uva.nl; 4 Department of Geriatric Medicine, Radboud University Medical Center, 6525 GC Nijmegen, The Netherlands; yvonne.schoon@radboudumc.nl

**Keywords:** accidental falls, aged, prevention and control, exercise, independent living

## Abstract

This observational study was conducted to determine which factors are associated with frequent participation in a home-based exercise program. The effects of frequent participation on health-related outcomes over time are investigated, as well. Community-dwelling adults aged ≥65 years participated in a twelve-week home-based exercise program. The program consisted of an instruction book with exercises that were performed individually at home. Frequent participation was classified as performing exercises of the instruction book daily or a few days a week during the study period. A logistic regression analysis was performed to determine the association between factors (i.e., demographic and health-related characteristics) and frequent participation. Furthermore, to investigate the effects of frequent participation on health-related outcomes, generalized linear and logistic regression models were built. A total of 238 participants (mean age 81.1 years (SD ± 6.7), 71% female) were included in the study. Frequent participation during the study period was indicated by fifty-two percent of participants. Analyses showed that a higher degree of pain (OR: 1.02, 95% CI: 1.–1.04) was associated with frequent participation. In addition, the effect of frequent participation over time was a significant improvement in current health perceptions (B: 4.46, SE: 1.99).

## 1. Introduction

Falls among older adults are a global public health problem, with high levels of healthcare consumption and high costs [1,2]. In the Netherlands, from the years 2000 to 2017, the population aged ≥65 years increased by 47% to 3.2 million [3]. During that time, fall-related emergency department visits increased by 87% to 124,000 per year [4]. Due to an aging population, the number of falls is expected to further increase in the coming decades. Fortunately, previous studies have shown that prevention programs can reduce falls among community-dwelling older adults [5,6,7,8]. The majority of the proven-effective programs consist of an exercise component [5,6,7,8]. Even though many exercise programs are offered in a group on location, older adults appear to favor an individual, home-based exercise program [9]. Advantages of a home-based program are good accessibility and lower program costs, as no exercise room or physiotherapist needs to be arranged. The level of participation of older adults in home-based exercise programs is generally low [10]. However, high participation levels may lead to a reduced fall risk [11]. A systematic review and meta-analysis investigated the relationship between program features, level of participation, and the effectiveness of home-based exercise programs [10]. No association was found between level of participation and the effectiveness of a program; however, an association between program features and level of participation was found. Namely, including walking and balance exercise in the program and providing home visit support were associated with a higher level of participation in the program. To our knowledge, no study has investigated the association between participant characteristics, the level of participation, and the effectiveness of a home-based exercise program. This information could help in the planning of future falls prevention interventions. Therefore, the aims of this paper were to: (1) determine which participant characteristics are associated with frequent participation of community-dwelling older adults in a home-based exercise program; and (2) investigate the effects of frequent participation on health-related outcomes over time.

## 2. Materials and Methods

### 2.1. Study Design and Population

In an observational study, community-dwelling adults aged ≥65 years, living in the city of Breda, in the Netherlands, were included in the study. Older adults living in a residential care facility, not understanding the Dutch language, or those with dementia were excluded from participation. All participants were offered a home-based exercise program for twelve weeks. At baseline and after twelve weeks of follow-up, a questionnaire was administered during home visits. Written informed consent was provided by all participants. The medical ethics committee of Erasmus MC, University Medical Center Rotterdam waived ethical approval of the study (number 2017-139).

### 2.2. Home-Based Exercise Program

The home-based exercise program was based on the Senior Step intervention [12]. In the Senior Step intervention, participants performed self-tests for assessing mobility and fall risk. The safety and feasibility of these tests were evaluated in that study. Apart from the self-tests, participants were also offered an instruction book with exercises. The instruction book was developed by two physiotherapists, and was based on the Otago program [13]. The book consists of exercises to: (1) promote safe use of walking aids, (2) improve mobility, (3) improve reaching (i.e., forwards, sideways, and backwards), (4) improve quality of walking and walking speed, and (5) improve overall fitness (i.e., agility, strength, balance, and conditioning). Amongst others, exercises consisted of walking up and down a slope with a walking aid, standing up from a chair, reaching for a kitchen cupboard, walking a figure eight, or walking up and down the stairs. Additional information on the instruction book can be found in Supplementary Materials S3. The book is divided into four levels, ranging from simple, low-intensity to complex, intensive exercises. In the current study, the instruction book of the Senior Step intervention was offered to participants as a home-based exercise program, for twelve weeks. At baseline, a member of the research team visited the participant at home. During this home visit, the researcher gave the participant the instruction book, explained how to use it, and advised the participant about which exercise level would be appropriate for beginning the program. However, during the study period, participants could change which exercises they performed, and how often, without interference from the research team. After twelve weeks of follow-up, a member of the research team called the participant to schedule a second home visit. During this home visit, the instruction book was returned to the research team.

### 2.3. Recruitment

Between March 2017 and March 2018, participants were recruited through primary care health professionals, such as community nurses, physiotherapists, and general practitioners. Furthermore, information sessions and workshops were held in community centers, advertisements were published in local papers, and commercials were broadcasted on a local television and radio channel. Older adults could apply to participate in the study by telephone, regular mail, or email. When the research team received the application, participants were sent an informed consent form by regular mail. When the research team did not receive a signed informed consent form, the participant was reminded by telephone, regular mail, or email. Participants were called to schedule a first home visit when the research team received a signed informed consent form.

### 2.4. Outcome Variables

#### 2.4.1. Participant Characteristics at Baseline and Follow-Up

Data collection took place between July 2017 and June 2018, as baseline home visits were performed between July 2017 and March 2018, and follow-up home visits were performed between October 2017 and June 2018. The baseline and follow-up questionnaire on participant characteristics included demographic characteristics, such as sex, age, living situation (i.e., alone or with someone else), and education. Education was classified as low (i.e., less than primary school, primary school, and little more than primary school), middle (i.e., technical school, vocational education, general secondary/pre-university education), and high (i.e., college/university). Several self-reported measurements were performed, as well. The five-dimensional EuroQol instrument (EQ-5D) and the domain cognition assessed the generic quality of life on the dimensions mobility, self-care, usual activities, pain and discomfort, anxiety and depression, and cognitive function [14]. Mean scores range from 0 (death) to 1 (full health). The fall risk test (part of a fall analysis assessment) determined that an elevated fall risk was present when a participant had a fall in the past twelve months, or the participant had mobility problems and a fear of falling [15,16]. The Timed “Up & Go” (TUG) measured the mobility, by measuring the time in seconds it took to stand up from a chair, walk three meters back and forth, and sit down again [17]. The Short Falls Efficacy Scale-International (Short FES-I) assessed the concern about falling [18]. The Self-Management Ability Scale Shorter (SMAS-S) determined self-management abilities, which was based on taking initiative, investment behavior, variety, multifunctionality, self-efficacy, and positive frame of mind [19]. Scores range from 0–100, a higher score means better self-management abilities. The Short-Form General Health Survey of the Medical Outcomes Study (SF-20) measured general health, which was based on physical functioning, role functioning, social functioning, mental health, current health perceptions, and pain [20]. Scores range from 0–100, a higher score means better functioning, and for pain, a higher score means a higher degree of pain.

#### 2.4.2. Level of Participation

After twelve weeks of follow-up, participants were asked how often they had performed the exercises outlined in the instruction book. Participants reported that the exercises were performed daily, a few days a week, one day a week, less than one day a week, or not at all. A review by Sherrington et al. (2016) has shown that participating in a falls prevention exercise program, for at least three hours a week, could reduce the fall rate among older adults [6]. We estimated that, in order to reach three hours of exercise, an individual should at least exercise a few days a week. Therefore, we decided to classify an individual who performed exercises daily or a few days a week as having ‘frequent participation’. ‘Infrequent or nonparticipation’ was classified as performing exercises one day a week, less than one day a week, or not at all. The reasons study participants gave for not frequently participating were recorded after twelve weeks as well. As the level of participation is based on self-report, it could have been influenced by social desirability bias. By consistently collecting data in the same way during home visits, an attempt was made to minimize potential bias. 

### 2.5. Statistical Analyses

#### 2.5.1. Baseline Characteristics

The frequencies of baseline characteristics of all participants were determined. Continuous variables are expressed as mean and standard deviation (SD), and dichotomous variables are expressed as number (n) and percentage (%). 

#### 2.5.2. Association between Factors and Frequent Participation

In order to determine the baseline differences between frequent and infrequent or nonparticipating individuals, an independent samples *t*-test was performed on continuous variables, whereas a Chi-squared test was performed on dichotomous variables. These analyses were performed to determine the baseline differences between completers and dropouts, as well. The association between factors and frequent participation was determined by performing a logistic regression analysis, in which frequent participation was used as a dependent variable. Factors included the baseline demographic characteristics sex, age, living situation, and education. Furthermore, the follow-up health-related outcomes quality of life, fall risk, mobility, fear of falling, self-management, and general health were included. In the univariate logistic regression analysis, the crude association between the factors and frequent participation was calculated. The variables that had a crude association with frequent participation, with a *p*-value < 0.20, were selected for multivariable model 1. An adjustment for other baseline confounders was performed in multivariable model 2. The confounders of model 2 were selected by investigating the baseline differences between the frequent and infrequent or nonparticipation groups. If a variable differed between the groups with a *p*-value < 0.20, this variable was selected. Results of the univariate and multivariable analysis are expressed as odds ratio (OR) and 95% confidence interval (CI). 

#### 2.5.3. Effects of Frequent Participation on Health-Related Outcomes

To investigate the effects of frequent participation on health-related outcomes, generalized linear and logistic regression models were built, in which follow-up health-related outcomes were used as a dependent variable. A generalized linear regression analysis was performed on continuous health-related outcomes and a generalized logistic regression analysis was performed on dichotomous health-related outcomes. Health-related outcomes included follow-up quality of life, fall risk, mobility, concern about falling, self-management, and general health. In multivariable model 1, the crude effect of frequent participation on health-related outcomes was adjusted for the baseline variable. An adjustment for the baseline variable and other baseline confounders was performed in multivariable model 2. The confounders of model 2 were selected by investigating the baseline differences between the frequent and infrequent or nonparticipation groups. If a variable differed between the groups with a *p*-value < 0.20, this variable was selected for multivariable model 2. Results of the logistic regression analysis are expressed as OR and 95% CI, whereas results of the linear regression analyses are expressed as Beta (B) and standard error (SE). 

#### 2.5.4. Multicollinearity

To take into account the presence of multicollinearity [21], multiple analyses were performed. The correlations between all independent variables were calculated, and expressed as Pearson correlation coefficients (r). The correlation between two variables is assumed to be low (r = 0.30–0.50), moderate (r = 0.50–0.70), or high (r = 0.70–0.90) [22]. Another way to detect multicollinearity is by calculating a variance inflation factor (VIF). A VIF has been calculated for all independent variables, of which a VIF larger than five or ten is suggested to detect multicollinearity [23].

As baseline and follow-up questionnaires were filled in properly (less than 9% missing data), no imputation measures were considered. A *p*-value < 0.05 was considered statistically significant. All analyses were performed using SPSS Statistical Data software (IBM), version 24. 

## 3. Results

### 3.1. Baseline Characteristics

**Figure 1:**
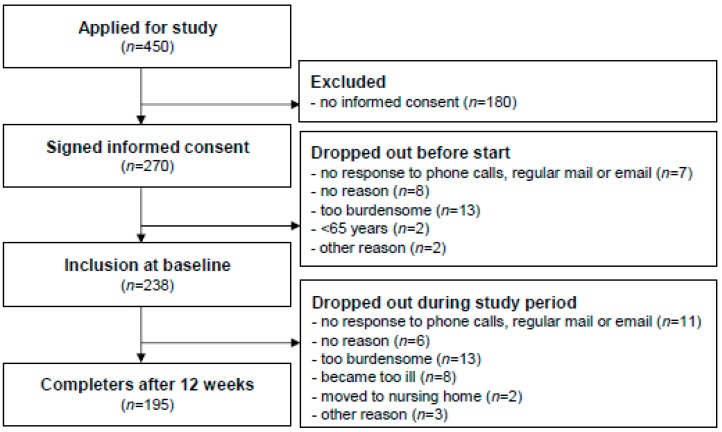
Flowchart of participants in the home-based exercise program.

A total of 238 adults aged ≥65 years participated in the study, of which 195 participants (82%) completed the twelve-week program (Figure 1). The mean age of all participants was 81.1 years (SD ± 6.7) (Table 1). The majority of participants were women (71%), living alone (63%), with a middle level education (55%), and with an elevated fall risk (69%). No statistically significant baseline differences between completers and dropouts were observed (Supplementary Materials Table S1).

### 3.2. Association between Factors and Frequent Participation

Fifty-two percent (*n* = 102/195) of the participants performed exercises of the home-based exercise program daily (*n* = 57) or a few days a week (*n* = 45), and so were classified as the frequent participation group. The infrequent or nonparticipation group consisted of participants performing exercises one day a week (*n* = 18), less than one day a week (*n* = 22), or not at all (*n* = 53). The most important reasons for not frequently participating, mentioned by the participants, were ‘exercises are too easy’ (29%), and ‘poor health’ (21%). At baseline, the demographic characteristics did not significantly differ between the frequent and infrequent or nonparticipation groups (Table 1). Health-related outcomes did differ significantly between the two groups, as the frequent participation group had a significantly higher quality of life, better self-management abilities, and better physical functioning (SF-20), than the infrequent or nonparticipation group. A significantly higher degree of pain (SF-20) was indicated by the frequent participation group, as well.

In the univariate logistic regression analysis, a higher quality of life and better self-management abilities were significantly associated with frequent participation (Table 2). One factor that was not significantly associated in the univariate analysis, was significantly associated in multivariable models 1 and 2. Namely, a higher degree of pain (SF-20) was associated with frequent participation in multivariable model 1 (OR: 1.02, 95% CI: 1.01–1.04) and model 2 (OR: 1.02, 95% CI: 1.00–1.04).

### 3.3. Effects of Frequent Participation on Health-Related Outcomes

In multivariable model 1, the effect of frequent participation over time was a significant improvement in current health perceptions (SF-20) (B: 4.49, SE: 2.01) (Table 3). This effect was observed in multivariable model 2, as well (B: 4.46, SE: 1.99). The direction of this change can be derived from Supplementary Materials Table S2, which shows the baseline and follow-up health-related outcomes of individuals frequently participating in the program.

### 3.4. Multicollinearity

As shown in Table 2, the variable pain was not significantly associated in the univariate analyses, but was significantly associated in multivariable model 1 and 2. Therefore, multicollinearity could have influenced the results. The variable pain has a low correlation (0.30–0.50) with six independent variables. Furthermore, this variable does not have a VIF larger than three.

## 4. Discussion

The current study showed that 52% of the participants performed the exercises of the home-based exercise program frequently during the entire study period. At baseline, the frequent participation group had a significantly higher quality of life, better self-management abilities, better physical functioning, and a higher degree of pain than the infrequent or nonparticipation group. A higher degree of pain was associated with frequent participation. Also, our study observed that the effect of frequent participation over time was a significant improvement in current health perceptions.

Several other studies have found that participation in an exercise program resulted in better health perceptions. Namely, three studies have shown that, among older adults, participation in a community exercise group, in a Pilates exercise group, or in Tai Chi has resulted in better health perceptions [24,25,26].

Frequent participation in the program was not associated with a lower fall risk or better mobility. An explanation could be that the study period was not long enough to detect clear associations or effects. For example, a meta-analysis of four Otago studies showed a reduction in falls and improvement in balance; however, fall events and balance were monitored for at least 44 weeks, whereas our study had only twelve weeks of follow-up [27].

A positive association between good self-rated health and participation has been observed in previous studies. Namely, among Mexican older adults, good self-rated health was associated with practicing regular physical activity [28]; among community-dwelling Japanese older adults, it was associated with high participation in sports groups [29]; and among community-dwelling white American older adults, it was associated with engaging in medium- or high-intensity activity [30]. In our study, we did not observe an association between good self-rated general health (SF-20) and frequent participation. An explanation for these differences in study results could be that ‘exercise participation’ was defined differently. Specifically, the cohort studies performed in Mexico, Japan, and in the United States classified participation as all physical activity that was performed in the past twelve months, whereas we classified participation as physical activity that was performed only in the exercise program. Furthermore, Hawley-Hague et al. (2016) published a review on studies reporting the level of participation of older adults in exercise programs [31]. They showed that there was hardly any consensus between studies on how to define the level of participation. 

Instead of an association between good self-rated health and participation, we observed that a higher degree of pain (SF-20) was associated with frequent participation. This could partly be explained by differences between participation groups at baseline. Specifically, at baseline, the frequent participation group had a higher degree of pain than the infrequent or nonparticipation group. However, the question remains why individuals with a higher degree of pain were more likely to participate frequently in the exercise program. A systematic review on older adults’ perspectives reported that pain can be a barrier or facilitator for participation [32]. Even though exercise can be perceived as physically demanding, some older adults exercise in order to deal with or relieve pain.

The percentage of individuals with frequent participation was relatively high in our study (52%), as a systematic review and meta-analysis showed that, on average, 21% of older adults are adherent in exercise interventions [10]. This can be explained by the fact that the program of the current study included balance and walking exercise, which have shown to increase participant adherence [10]. The participation level of the individuals who participated infrequently or not at all might have been higher if the exercises had been more challenging, as 29% of the participants mentioned that the exercises were too easy. A study by Elskamp et al. (2012) reported a similar result, as the older adults that refused to participate in their falls prevention interventions considered themselves to be too healthy [33].

Interestingly, a relatively old population (mean age 81.1 years) participated in the current study. The fact that the majority of participants were recruited through primary care health professionals, such as community nurses, could be an explanation. As in general, the oldest old receive homecare, this could have resulted in a relatively old population in the study. Another explanation could be that ‘younger’ older adults (i.e., those aged 64–75 years) prefer a group-based falls prevention program, whereas the oldest old prefer a home-based program [9].

As reported earlier in the results, the presence of multicollinearity could be an explanation for the variable pain to become statistically significant in the multivariable model 1 and 2. However, as the correlation with other independent variables is low, and it did not have a VIF larger than three, it is unlikely that multicollinearity has influenced the results. 

A strength of our study is that it resembles a real-life situation. Namely, in comparison to a program offered in a group on location, the participants of the current study could choose which home-based exercises they performed, and how often, without interference from the research team or a physiotherapist. Another strength of the study was that adherence was good, as 82% of the participants completed the program, with few avoidable dropouts. Furthermore, no statistically significant baseline differences between completers and dropouts were observed.

A limitation of our study is that the level of participation was based on self-report and so is subject to social desirability bias. If participation had been monitored by the research team during the study period, participation levels might have been different. For example, if participation levels had been measured again after six weeks, the research team could have guided participants to exercise more often, which could have changed participation levels. However, this would have detracted from the real-life nature of this falls prevention program. In addition, the level of participation was administered with an ordinal scale. This has reduced the precision of the measurement. Another limitation was that no information was available on what type or duration of exercises of the instruction book were performed by the participants. Therefore, we were unable to determine what type or duration may have contributed to the changes in the outcomes. Furthermore, other physical activity, performed outside of the study, was not assessed and thus could not be adjusted for in the analyses. The studied population might not be representative of general community-dwelling older adults. Specifically, as the majority of older adults were recruited through primary care health professionals, the participants of the current study might have been older and more frail than the general older adult population. Therefore, selection bias might have been present.

## 5. Conclusions

This study shows that the participant characteristic pain was associated with frequent participation in a home-based exercise program. Furthermore, the effect of frequent participation over time was a significant improvement in current health perceptions (SF-20). These characteristics should be taken into account in the planning of future falls prevention interventions. By monitoring these characteristics during an intervention, it is possible to guide and motivate specific participants, so that frequent participation rates will increase.

## Figures and Tables

**Table 1:** Baseline characteristics, and differences between individuals frequently and infrequently or not participating in the home-based exercise program.

	All Participants (*n* = 238)	Total Frequent and Infrequent or Nonparticipation (*n* = 195)	Frequent Participation (*n* = 102)	Infrequent or Nonparticipation (*n* = 93)	Difference between Frequent and Infrequent or Nonparticipation *
Demographic characteristics	*n* (%)	*n* (%)	*n* (%)	*n* (%)	*p*-value
Female	169 (71)	140 (72)	76 (75)	64 (69)	0.38
Age—mean ± SD	81.1 ± 6.7	80.9 ± 6.6	80.6 ± 6.4	81.3 ± 6.8	0.46
Living alone	151 (63)	124 (64)	66 (65)	58 (62)	0.73
Education					
low	70 (29)	53 (27)	24 (24)	29 (31)	0.23
middle	130 (55)	108 (55)	56 (55)	52 (56)	0.89
high	38 (16)	34 (17)	22 (22)	12 (13)	0.11
Health-related outcomes	mean ± SD	mean ± SD	mean ± SD	mean ± SD	*p*-value
Quality of life (EQ-5D + cognition) ^1^	0.64 ± 0.24	0.65 ± 0.24	0.70 ± 0.23	0.60 ± 0.24	0.00
Elevated fall risk—n (%)	161 (69) ^a^	133 (69) ^d^	65 (64) ^g^	68 (75) ^i^	0.12
Mobility (TUG) in seconds	17.0 ± 9.1 ^b^	16.9 ± 8.9 ^e^	16.2 ± 7.9 ^h^	17.6 ± 10.0 ^j^	0.29
Concern about falling (Short FES-I)	9.8 ± 4.0	9.8 ± 3.9	9.9 ± 3.6	9.7 ± 4.1	0.77
Self-management (SMAS-S) ^2^	59.3 ± 16.2 ^c^	60.1 ± 16.0 ^f^	63.8 ± 14.9	56.1 ± 16.3 ^k^	0.00
General health (SF-20) ^3^					
physical functioning	45.1 ± 31.7 ^c^	45.1 ± 31.6 ^f^	50.2 ± 32.0	39.5 ± 30.2 ^k^	0.02
role functioning	28.8 ± 41.4	29.2 ± 41.1	34.3 ± 43.9	23.7 ± 37.3	0.07
social functioning	72.5 ± 34.0	74.5 ± 32.7	76.3 ± 32.5	72.5 ± 33.0	0.42
mental health	73.0 ± 20.7 ^c^	73.2 ± 20.8 ^f^	74.1 ± 20.8 ^g^	72.3 ± 20.7	0.55
current health perceptions	46.4 ± 21.1 ^c^	46.9 ± 21.1 ^f^	47.1 ± 20.5	46.7 ± 21.9 ^k^	0.89
pain	33.0 ± 27.6	31.9 ± 27.8	35.8 ± 27.1	27.7 ± 27.9	0.04

SD: Standard deviation; ^1^: Mean scores range from 0 (death) to 1 (full health); ^2^: Scores range from 0–100, a higher score means better self-management abilities; ^3^: Scores range from 0–100, a higher score means better functioning, and for pain, a higher score means a higher degree of pain; ^a^: *n* = 235; ^b^: *n* = 217, as twenty-one participants were not able to do the test; ^c^: *n* = 237; ^d^ *n* = 192; ^e^ *n* = 178, as seventeen participants were not able to do the test; ^f^ *n* = 194; ^g^ *n* = 101; ^h^ *n* = 96, as six participants were not able to do the test; ^i^ *n* = 91; ^j^ *n* = 82, as eleven participants were not able to do the test; ^k^ *n* = 92; *: Independent samples *t*-test for continuous variables, Chi-squared test for dichotomous variables. A *p*-value < 0.05 is considered a statistically significant difference.

**Table 2:** Factors associated with frequent participation in the home-based exercise program.

	Univariate		Multivariable Model 1 ^†^		Multivariable Model 2 ^‡^	
	OR (95% CI)	*p*-value	OR (95% CI)	*p*-value	OR (95% CI)	*p*-value
Female	1.33 (0.71–2.48)	0.38	1.40 (0.66–2.98)	0.38	1.12 (0.50–2.47)	0.79
Age	0.98 (0.94–1.03)	0.46	1.01 (0.96–1.06)	0.78	1.01 (0.95–1.06)	0.82
Living alone	1.11 (0.62–1.98)	0.73	0.88 (0.45–1.74)	0.72	0.82 (0.40–1.68)	0.59
Education						
low	0.68 (0.36–1.28)	0.23	1.14 (0.55–2.37)	0.72	1.51 (0.68–3.36)	0.32
middle	0.96 (0.55–1.69)	0.89	0.88 (0.42–1.82)	0.72	0.66 (0.30–1.48)	0.32
high	1.86 (0.86–4.00)	0.12	1.93 (0.83–4.46)	0.13	1.47 (0.60–3.62)	0.40
Quality of life (EQ-5D + cognition)	3.52 (1.16–10.71)	0.03	1.39 (0.21–9.31)	0.73	0.63 (0.07–6.12)	0.63
Elevated fall risk	0.72 (0.41–1.27)	0.25	1.00 (0.49–2.03)	1.00	0.96 (0.43–2.18)	0.93
Mobility (TUG) in seconds	0.99 (0.96–1.03)	0.72	1.02 (0.97–1.07)	0.47	1.01 (0.95–1.06)	0.84
Concern about falling (Short FES-I)	1.02 (0.96–1.09)	0.50	1.07 (0.98–1.17)	0.12	1.08 (0.98–1.18)	0.14
Self-management (SMAS-S)	1.03 (1.01–1.05)	0.00	1.02 (1.00–1.05)	0.09	1.01 (0.98–1.04)	0.64
General health (SF-20)						
physical functioning	1.01 (1.00–1.02)	0.07	1.00 (0.99–1.02)	0.67	1.00 (0.99–1.02)	0.69
role functioning	1.01 (1.00–1.01)	0.09	1.00 (0.99–1.01)	0.50	1.01 (0.99–1.02)	0.41
social functioning	1.01 (1.00–1.01)	0.27	1.00 (0.99–1.01)	0.89	1.00 (0.99–1.01)	0.91
mental health	1.00 (0.99–1.02)	0.70	0.99 (0.97–1.01)	0.20	0.98 (0.96–1.00)	0.98
current health perceptions	1.01 (1.00–1.02)	0.14	1.01 (0.99–1.03)	0.27	1.01 (0.99–1.04)	0.19
pain	1.01 (1.00–1.02)	0.10	1.02 (1.01–1.04)	0.00	1.02 (1.00–1.04)	0.02

^†^: Adjusted for baseline high education, and follow-up quality of life, self-management, physical functioning, role functioning, current health perceptions, and pain; ^‡^: Adjusted for baseline high education, quality of life, elevated fall risk, self-management, physical functioning, role functioning and pain, and follow-up quality of life, self-management, physical functioning, role functioning, current health perceptions, and pain; A *p*-value <0.05 is considered a statistically significant difference.

**Table 3:** Effects of frequent participation in the home-based exercise program on health-related outcomes.

	Multivariable Model 1 ^†^		Multivariable Model 2 ^‡^	
Logistic Regression	OR (95% CI)	*p*-value	OR (95% CI)	*p*-value
Elevated fall risk	0.85 (0.43–1.71)	0.65	0.90 (0.42–1.94)	0.79
Linear regression	B (SE)	*p*-value	B (SE)	*p*-value
Quality of life (EQ-6D)	0.01 (0.03)	0.78	0.01 (0.03)	0.82
Mobility (TUG) in seconds	−0.10 (0.94)	0.92	−0.57 (0.91)	0.53
Concern about falling (Short FES-I)	0.30 (0.50)	0.55	0.56 (0.51)	0.27
Self-management (SMAS-S)	1.79 (1.55)	0.25	1.20 (1.58)	0.45
General health (SF-20)				
physical functioning	0.59 (3.49)	0.87	1.38 (3.53)	0.70
role functioning	2.91 (4.23)	0.49	2.43 (4.08)	0.55
social functioning	3.51 (4.32)	0.42	1.97 (4.37)	0.65
mental health	−0.29 (2.01)	0.89	−0.49 (2.06)	0.81
current health perceptions	4.49 (2.01)	0.03	4.46 (1.99)	0.03
pain	3.04 (3.59)	0.40	6.62 (3.60)	0.07

^†^: Adjusted for the baseline variable; ^‡^: Adjusted for the baseline variable, and baseline high education, quality of life, elevated fall risk, self-management, physical functioning, role functioning, and pain; A *p*-value < 0.05 is considered a statistically significant difference.

## Supplementary Materials

The following are available online at https://www.mdpi.com/1660-4601/16/6/1087/s1, Table S1: Baseline characteristics of completers and dropouts of the home-based exercise program, and differences between both groups. Table S2: Baseline and follow-up health-related outcomes of individuals frequently participating in the home-based exercise program. S3: Instruction book of the home-based exercise program.
