# Supplementary material for: Factors Associated with Participation of Community-Dwelling Older Adults in a Home-Based Falls Prevention Program
Source: Int J Environ Res Public Health. 2019 Mar 26;16(6):1087. doi: 10.3390/ijerph16061087 (PMC6466058; doi:10.3390/ijerph16061087)
Supplement: Supplementary file 1 [file ijerph-16-01087-s001.zip › Supplementary Materials Table S1 + S2.docx]

Supplementary Materials

**Table S1.** Baseline characteristics of completers and drop-outs of the home-based exercise program, and differences between both groups.

|  | **Completers**  **(n=195)** | **Drop-outs**  **(n=43)** | **Difference between completers and drop-outs*** |
| --- | --- | --- | --- |
| **Demographic characteristics** | *n (%)* | *n (%)* | *p-value* |
| Female | 140 (72) | 29 (67) | 0.57 |
| Age - mean ± SD | 80.9 ± 6.6 | 82.2 ± 6.9 | 0.24 |
| Living alone | 124 (64) | 27 (63) | 0.92 |
| Education  low  middle  high | 53 (27)  108 (55)  34 (17) | 17 (40)  22 (51)  4 (9) | 0.11  0.62  0.19 |
| **Health-related outcomes** | *mean* *± SD* | *mean* *± SD* | *p-value* |
| Quality of life (EQ-5D + cognition)^1^ | 0.65 ± 0.24 | 0.61 ± 0.23 | 0.35 |
| Elevated fall risk - n (%) | 133 (69)^a^ | 28 (65) | 0.60 |
| Mobility (TUG) in seconds | 16.9 ± 8.9^b^ | 17.8 ± 9.8^d^ | 0.58 |
| Concern about falling (Short FES-I) | 9.8 ± 3.9 | 9.7 ± 4.7 | 0.84 |
| Self-management (SMAS-S)^2^ | 60.1 ± 16.0^c^ | 55.4 ± 16.5 | 0.08 |
| General health (SF-20)^3^  physical functioning  role functioning  social functioning  mental health  current health perceptions  pain | 45.1 ± 31.6^c^  29.2 ± 41.1  74.5 ± 32.7  73.2 ± 20.8^c^  46.9 ± 21.1^c^  31.9 ± 27.8 | 45.0 ± 32.4  26.7 ± 42.7  63.7 ± 38.6  72.3 ± 20.5  44.1 ± 21.4  37.8 ± 26.9 | 0.98  0.72  0.10  0.79  0.43  0.21 |

SD: Standard deviation; ^1^: Mean scores range from 0 (death) to 1 (full health); ^2^: Scores range from 0-100, a higher score means better self-management abilities; ^3^: Scores range from 0-100, a higher score means better functioning, and for pain, a higher score means a higher degree of pain; ^a^: n=192; ^b^: n=178, as seventeen participants were not able to do the test; ^c^: n=194; ^d^: n=39, as four participants were not able to do the test; *: Independent samples t-test for continuous variables, Chi-squared test for dichotomous variables. A p-value <0.05 is considered a statistically significant difference.

**Table S2.** Baseline and follow-up health-related outcomes of individuals frequently participating in the home-based exercise program.

|  | **Baseline frequent participation**  **(n=102)** | **Follow-up frequent participation**  **(n=102)** |
| --- | --- | --- |
| **Health-related outcomes** | *mean* *± SD* | *mean* *± SD* |
| Quality of life (EQ-5D + cognition)^1^ | 0.70 ± 0.23 | 0.70 ± 0.26^c^ |
| Elevated fall risk - n (%) | 65 (64)^a^ |  |
| Mobility (TUG) in seconds | 16.2 ± 7.9^b^ | 16.9 ± 9.1^d^ |
| Concern about falling (Short FES-I) | 9.9 ± 3.6 | 9.9 ± 4.3^c^ |
| Self-management (SMAS-S)^2^ | 63.8 ± 14.9 | 61.6 ± 14.5 |
| General health (SF-20)^3^  physical functioning  role functioning  social functioning  mental health  current health perceptions  pain | 50.2 ± 32.0  34.3 ± 43.9  76.3 ± 32.5  74.1 ± 20.8^a^  47.1 ± 20.5  35.8 ± 27.1 | 51.7 ± 31.1^e^  36.3 ± 42.3  73.9 ± 33.4  75.1 ± 20.8  49.0 ± 19.4^c^  36.0 ± 26.7 |

SD: Standard deviation; ^1^: Mean scores range from 0 (death) to 1 (full health); ^2^: Scores range from 0-100, a higher score means better self-management abilities; ^3^: Scores range from 0-100, a higher score means better functioning, and for pain, a higher score means a higher degree of pain; ^a^: n=101; ^b^: n=96, as six participants were not able to do the test; ^c^: n=101; ^d^: n=82, as twenty participants were not able to do the test; ^e^: n=100.
