# Supplementary material for: Factors Associated with Participation of Community-Dwelling Older Adults in a Home-Based Falls Prevention Program
Source: Int J Environ Res Public Health. 2019 Mar 26;16(6):1087. doi: 10.3390/ijerph16061087 (PMC6466058; doi:10.3390/ijerph16061087)
Supplement: Supplementary file 1 [file ijerph-16-01087-s001.zip › Supplementary Materials Table S1 + S2.pdf]

Supplementary Materials

Table S1. Baseline characteristics of completers and drop-outs of the home-based exercise program, and differences between both groups.

	Completers (n=195)	Drop-outs (n=43)	Difference between completers and drop-outs*
**Demographic characteristics**	*n (%)*	*n (%)*	*p-value*
Female	140 (72)	29 (67)	0.57
Age - mean ± SD	80.9 ± 6.6	82.2 ± 6.9	0.24
Living alone	124 (64)	27 (63)	0.92
Education			
low	53 (27)	17 (40)	0.11
middle	108 (55)	22 (51)	0.62
high	34 (17)	4 (9)	0.19
**Health-related outcomes**	*mean ± SD*	*mean ± SD*	*p-value*
Quality of life (EQ-5D + cognition)[1]	0.65 ± 0.24	0.61 ± 0.23	0.35
Elevated fall risk - n (%)	133 (69)[a]	28 (65)	0.60
Mobility (TUG) in seconds	16.9 ± 8.9[b]	17.8 ± 9.8[d]	0.58
Concern about falling (Short FES-I)	9.8 ± 3.9	9.7 ± 4.7	0.84
Self-management (SMAS-S)[2]	60.1 ± 16.0[c]	55.4 ± 16.5	0.08
General health (SF-20)[3]			
physical functioning	45.1 ± 31.6[c]	45.0 ± 32.4	0.98
role functioning	29.2 ± 41.1	26.7 ± 42.7	0.72
social functioning	74.5 ± 32.7	63.7 ± 38.6	0.10
mental health	73.2 ± 20.8[c]	72.3 ± 20.5	0.79
current health perceptions	46.9 ± 21.1[c]	44.1 ± 21.4	0.43
pain	31.9 ± 27.8	37.8 ± 26.9	0.21

SD: Standard deviation; [1]: Mean scores range from 0 (death) to 1 (full health); [2]: Scores range from 0-100, a higher score means better self-management abilities; [3]: Scores range from 0-100, a higher score means better functioning, and for pain, a higher score means a higher degree of pain; [a]: n=192; [b]: n=178, as seventeen participants were not able to do the test; [c]: n=194; [d]: n=39, as four participants were not able to do the test; *: Independent samples t-test for continuous variables, Chi-squared test for dichotomous variables. A p-value <0.05 is considered a statistically significant difference.

Table S2. Baseline and follow-up health-related outcomes of individuals frequently participating in the home-based exercise program.

	**Baseline frequent participation (n=102)**	**Follow-up frequent participation (n=102)**
**Health-related outcomes**	*mean ± SD*	*mean ± SD*
Quality of life (EQ-5D + cognition)[1]	0.70 ± 0.23	0.70 ± 0.26[c]
Elevated fall risk - n (%)	65 (64)[a]	
Mobility (TUG) in seconds	16.2 ± 7.9[b]	16.9 ± 9.1[d]
Concern about falling (Short FES-I)	9.9 ± 3.6	9.9 ± 4.3[c]
Self-management (SMAS-S)[2]	63.8 ± 14.9	61.6 ± 14.5
General health (SF-20)[3]		
physical functioning	50.2 ± 32.0	51.7 ± 31.1[e]
role functioning	34.3 ± 43.9	36.3 ± 42.3
social functioning	76.3 ± 32.5	73.9 ± 33.4
mental health	74.1 ± 20.8[a]	75.1 ± 20.8
current health perceptions	47.1 ± 20.5	49.0 ± 19.4[c]
pain	35.8 ± 27.1	36.0 ± 26.7

SD: Standard deviation; [1]: Mean scores range from 0 (death) to 1 (full health); [2]: Scores range from 0-100, a higher score means better self-management abilities; [3]: Scores range from 0-100, a higher score means better functioning, and for pain, a higher score means a higher degree of pain; [a]: n=101; [b]: n=96, as six participants were not able to do the test; [c]: n=101; [d]: n=82, as twenty participants were not able to do the test; [e]: n=100.
