# Supplementary material for: Factors Associated with Participation of Community-Dwelling Older Adults in a Home-Based Falls Prevention Program
Source: Int J Environ Res Public Health. 2019 Mar 26;16(6):1087. doi: 10.3390/ijerph16061087 (PMC6466058; doi:10.3390/ijerph16061087)
Supplement: Supplementary file 1 [file ijerph-16-01087-s001.zip › S3. Instruction book of the home-based exercise program.pdf]

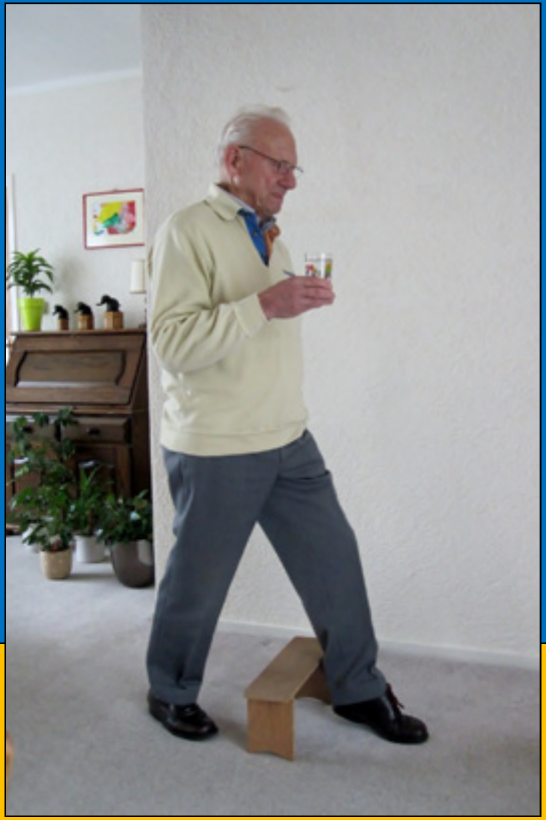

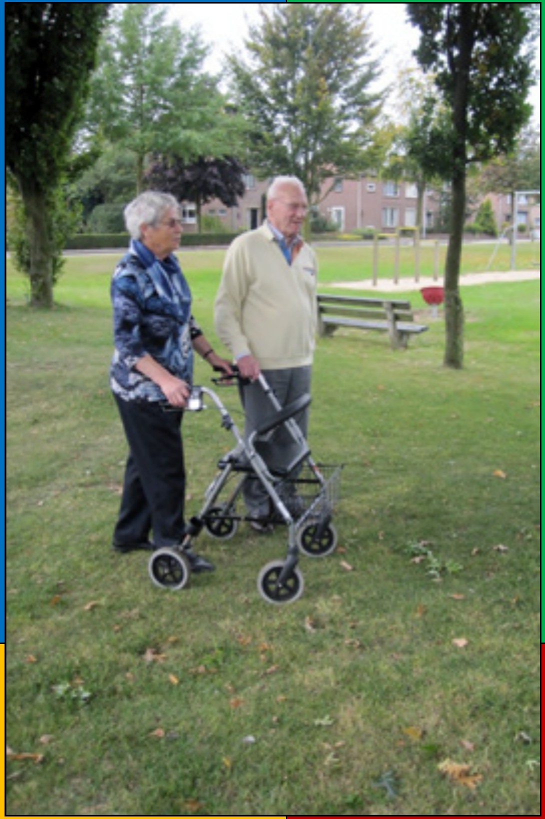

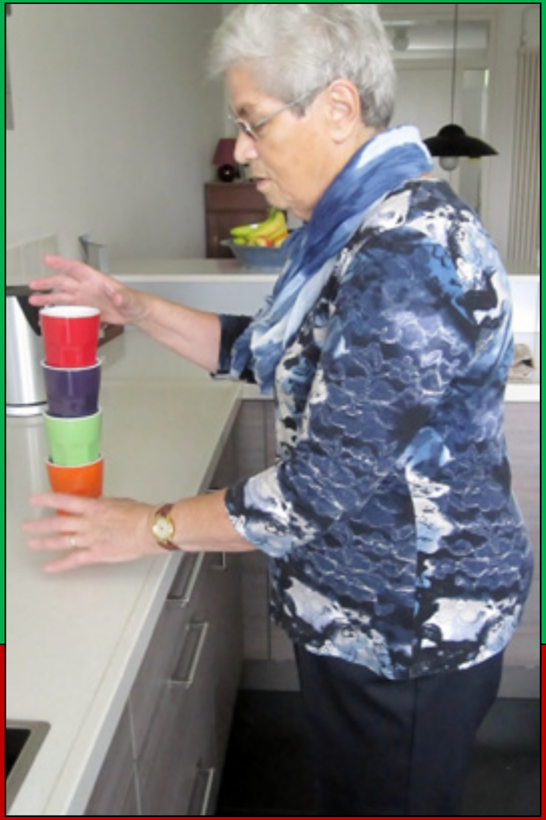

Oefenboek

Senior Stap Studie

©Afdeling Geriatrie, UMC St Radboud Nijmegen, 2012

Alle rechten voorbehouden. Niets uit deze uitgave mag worden verveelvoudigd, opgeslagen in een geautomatiseerd gegevensbestand, of openbaar gemaakt, in enige vorm of op enige wijze, hetzij elektronisch, mechanisch, door fotokopieën of opnamen, hetzij op enige andere manier, zonder voorafgaande schriftelijke toestemming van de eigenaar.

Inhoud

Afdeling Revalidatie, UMC St Radboud Nijmegen;

Miranda Jacobs, geriatriefysiotherapeut

Drs. Rogier van Oosten, geriatriefysiotherapeut i.o.

Dr. Ellen Smulders, onderzoeker en fysiotherapeut

Met dank aan

Mw. Jacobs-Beelen (figurant)

Dhr. Schoenmakers (figurant)

Maartje Graauwmans MSc (vormgeving)

ZonMW (subsidiegever)

In opdracht van

Afdeling Geriatrie, Senior Stap Studie

UMC St Radboud Nijmegen

Bij vragen kunt u contact opnemen met Branko Olij
010-7037773
b.olij@erasmusmc.nl

A
B
C
D

Inleiding

Door veroudering en lichamelijke klachten verandert het looppatroon. De kwaliteit van lopen kan hierbij in de loop der jaren verminderen waarbij uw lichaam zich soms ongemerkt aanpast. Het kan zijn dat u niet gemerkt heeft dat u langzamer bent gaan lopen, met kleinere passen of misschien zelfs meer schuifelend bent gaan lopen. Alledaagse bewegingen zoals lopen zonder loophulpmiddel, traplopen of bijvoorbeeld het opstaan van een stoel kunnen daardoor lastiger worden. Soms zelfs zo lastig dat er sprake is van onveilige situaties met risico op vallen of angst voor vallen. Dit kan leiden tot minder zelfstandig functioneren.

Ook is het mogelijk dat u eigenlijk nog goed ter been bent maar desondanks toch gevallen bent. Mogelijke oorzaken kunnen zijn dat u dingen doet die voor u onveilig zijn, dat u te snel handelt of misschien te veel dingen tegelijk doet.

Verandering van het bewegen bij het ouder worden

Het bewegingspatroon en daarmee het lopen verandert bij het ouder worden. Dit kan verschillende oorzaken hebben:

- Houdingsveranderingen, bijvoorbeeld krommer lopen
- Verminderd gevoel van evenwicht
- Afname spierkracht in benen
- Aandoeningen in het zenuwstelsel bijvoorbeeld een beroerte, ziekte van Parkinson of verminderd gevoel in uw voeten of benen
- Pijnlijke gewrichten
- Duizeligheid
- Slecht zien
- Vallen of valangst
- Verminderde conditie
- Verminderde lichamelijke activiteit

Het oefenboek

Dit oefenboek is een onderdeel van de Senior Stap Studie en kan u helpen om uw fitheid (balans, kracht, loopkwaliteit, loopsnelheid en conditie) te bevorderen. Het bevat oefeningen waarvan een groot deel bestaat uit alledaagse handelingen en bewegingen. Zo wordt het gemakkelijker om deze alledaagse activiteiten te benutten als oefenmoment.

Het boek bestaat uit de volgende onderdelen:

Loophulpmiddelen: hierbij wordt aandacht gegeven aan het veilig gebruik van loophulpmiddelen, zoals de rollator.

Oefeningen ter bevordering van het verplaatsen: denk hierbij aan het opstaan en gaan zitten op een stoel, het in en uit bed komen en het opstaan van de vloer.

Oefeningen ter bevordering van het reiken: hierbij komt het voorwaarts, zijwaarts en achterwaarts reiken aan de orde.

Looptraining: in dit onderdeel worden oefeningen en tips gegeven voor zowel in huis als buitenshuis, om de kwaliteit van lopen en de loopsnelheid te verbeteren.

Oefeningen ter verbetering van uw algehele fitheid: de oefeningen zijn gericht op het verbeteren van de lenigheid, kracht, balans en conditie.

De oefeningen worden aan de hand van foto´s afgebeeld en toegelicht met een korte instructie.

U kunt de oefeningen zelfstandig uitvoeren, of met hulp van uw partner, familie of andere mensen uit uw omgeving. Als u behandeld wordt door een fysiotherapeut kunt u uiteraard ook samen met de fysiotherapeut bekijken welke oefeningen voor u geschikt zijn.

Het oefenboek is opgedeeld in een algemeen hoofdstuk over loophulpmiddelen (paars). Er zijn vier verschillende profielen met ieder een eigen kleur waarbij er een verdeling is in AB (blauw/groene) en CD (oranje/rode) oefeningen. De kleur van de oefening kunt u ook zien aan de zijkant van de pagina.

Voor verdere uitleg zie p. 14 t/m 19 voor de AB-profielen en p. 46 t/m 51 voor de CD-profielen.

Welk profiel past bij u?

Niet alle oefeningen zijn geschikt voor u. Om de veiligheid van de oefeningen te waarborgen is het belangrijk dat u de oefeningen gaat doen uit het profiel wat bij u past.

Hiernaast volgt een kort overzicht per profiel. Bepaal wat uw loopsnelheid is. Als u werkt met de loopmeter dan kunt u uw loopsnelheid aan de hand hiervan bepalen. Werkt u niet met de loopmeter, dan zal de onderzoeker u vertellen wat uw loopsnelheid is. Lees daarna de bijbehorende stellingen aandachtig door. Dit kunt u eventueel samen met een familielid doen. Kies vervolgens het profiel dat het beste bij u past. Mocht het desondanks toch lastig blijken om het juiste profiel te bepalen dan kunt u de onderzoeker van de Senior Stap Studie raadplegen.

Profiel A (Blauw)

- U heeft een loopsnelheid van 0,4 t/m 1,4 km/u
- U maakt gebruik van een loophulpmiddel in huis
- U voelt zich onzeker en heeft hulp nodig om met een loophulpmiddel in huis te lopen
- U heeft hulp nodig bij activiteiten zoals aankleden, douchen of baden

Profiel B (Groen)

- U heeft een loopsnelheid van 1,4 t/m 2,2 km/u
- U maakt gebruik van een loophulpmiddel in huis
- U voelt zich onzeker om zonder loophulpmiddel in huis te lopen of grijpt bijvoorbeeld meubelstukken vast
- U maakt gebruik van een loophulpmiddel buitenshuis of komt nauwelijks buiten

Profiel C (Oranje)

- U heeft een loopsnelheid van 1,8 t/m 2,9 km/u
- U gebruikt geen loophulpmiddel in huis
- U gebruikt eventueel een loophulpmiddel buitenshuis

Profiel D (Rood)

- U heeft een loopsnelheid hoger dan 2,9 km/u
- U gebruikt geen loophulpmiddel in huis
- U gebruikt eventueel een loophulpmiddel buitenshuis
- U kunt zelfstandig traplopen

Loophulpmiddelen

Als het lopen verslechtert en u meer moeite krijgt om zich te verplaatsen kan het zijn dat een loophulpmiddel uitkomst biedt. Misschien loopt u inmiddels al met een loophulpmiddel. Soms is het echter lastig om te bepalen wat het juiste moment is om een loophulpmiddel in te zetten. Vooral als het lopen geleidelijk achteruit gaat. Bovendien kan ook een gevoel van schaamte een reden zijn waarom u er nog niet aan toe bent om de voordelen van een loophulpmiddel in te zien.

Tip:

Bekijk de checklist hiernaast. Indien u één van deze stellingen met ja kunt beantwoorden zou een loophulpmiddel een meerwaarde kunnen hebben.

Checklist

- U loopt binnenshuis met moeite en moet regelmatig steun nemen aan het meubilair
- U loopt buitenshuis met moeite en gaat daardoor minder vaak naar buiten
- U kunt alleen kleine afstanden lopen en staat vaak stil om te rusten
- U heeft moeite om uw evenwicht te bewaren bijvoorbeeld in het donker of als u moe bent
- U bent bang om te vallen en verplaatst zich daardoor minder
- U heeft moeite met het op- en afstappen van bijvoorbeeld stoepranden en traptreden
- U heeft moeite met in- of uitstappen bij de bus of trein

Stok

De stok wordt ook wel handstok of wandelstok genoemd en biedt ondersteuning bij lichte evenwichtsproblemen tijdens het lopen. Een stok kan een gevoel van veiligheid bieden in situaties waarbij u zich zonder loophulpmiddel onzeker begint te voelen en is zeer geschikt om te gebruiken als u bijvoorbeeld met het openbaar vervoer wilt reizen, langere afstanden gaat lopen of ondersteuning nodig heeft bij traplopen.

U kunt de stok op de juiste hoogte afmeten door uw arm losjes te laten hangen waarbij u de stok zodanig afstelt dat het handvat ter hoogte van de pols (horlogebandje) komt. De onderkant van de stok dient te bestaan uit een dop van antislipmateriaal waardoor u niet wegglijdt op de ondergrond.

De rollator

De rollator biedt meer stabiliteit dan een stok en is daarom een geschikt loophulpmiddel bij verminderde spierkracht, balans en/of duizeligheidsklachten.

De meeste rollators zijn zowel binnen als buiten te gebruiken.

Rollators zijn er in verschillende uitvoeringen met diverse accessoires (zoals een zitje, dienblad of stokhouder). Bij een inklapbare rollator is een vergrendeling belangrijk voor uw veiligheid. Het comfort van de rollator hangt sterk af van lichaamsbouw en persoonlijke voorkeur. Het is daarom handig om bij aankoop verschillende rollators uit te testen.

Goed onderhoud van de rollator is belangrijk voor uw veiligheid.

Veilig rollatorgebruik

Naast goed onderhoud is ook het veilig gebruik van de rollator belangrijk. De volgende tips en oefeningen kunt u gebruiken om op een veilige manier met uw rollator om te gaan.

1. Juiste hoogte van rollator .... p. 8
2. Loophouding rollator .... p. 8
3. Spullen vervoeren .... p. 8
4. Met rollator de stoep af .... p. 9
5. Met rollator de stoep op .... p. 10
6. Met rollator een helling af .... p. 11
7. Met rollator een helling op .... p. 11
8. Zitten op rollator .... p. 12
9. Opstaan van rollator .... p. 13

1. Juiste hoogte van rollator

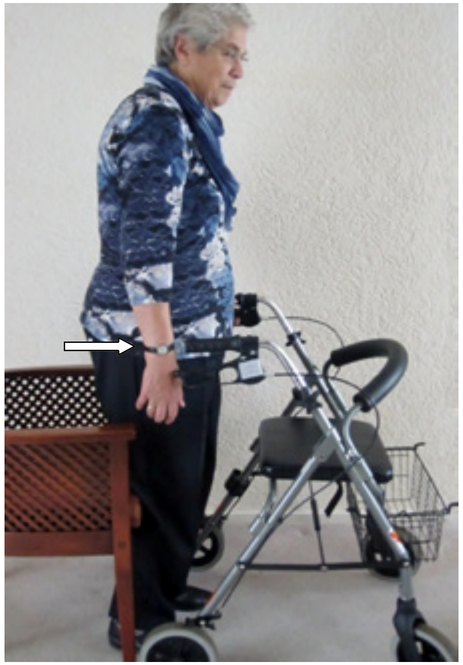

- Ga rechtop staan
- Laat uw armen losjes hangen
- De juiste rollatorhoogte: handvat ter hoogte van uw pols (horlogebandje)

2. Loophouding rollator

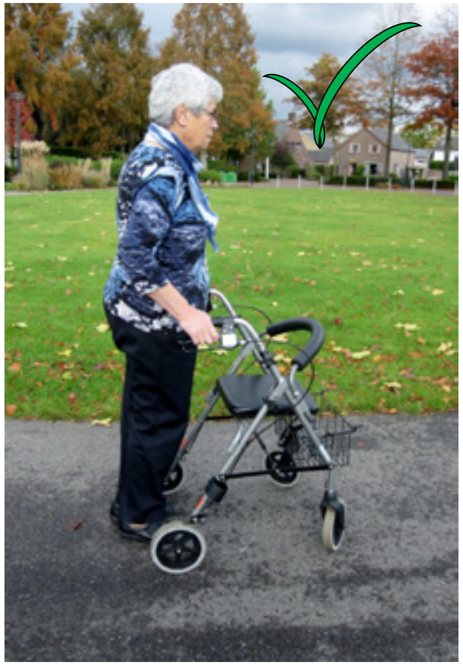

- U kunt de rollator alleen goed gebruiken als de handvatten op de juiste hoogte zijn ingesteld
- De juiste loophouding krijgt u door met uw voeten tussen de achterwielen te lopen

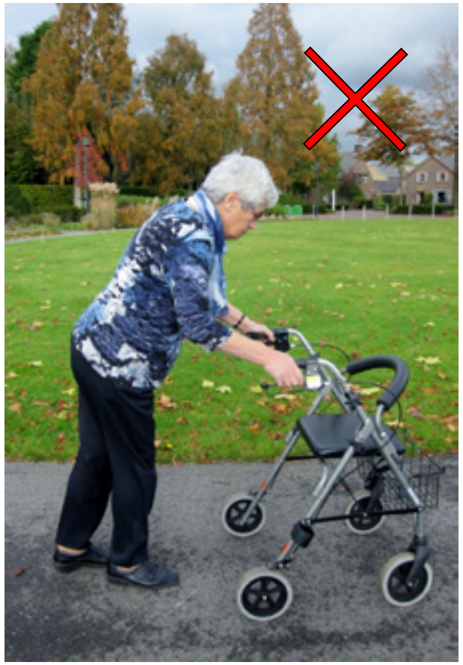

- Als de rollator te hoog staat, loopt u met opgetrokken schouders of te ver achter de rollator
- Als uw rollator te laag staat, gaat u voorovergebogen lopen

3. Spullen vervoeren

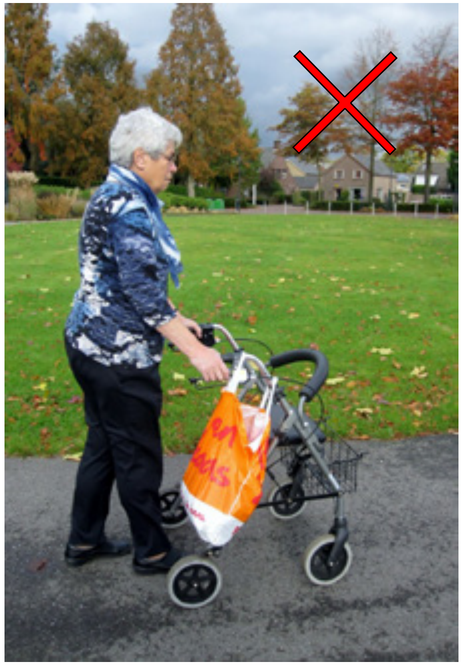

- Zorg ervoor dat u geen tassen aan het stuur hangt of teveel spullen meeneemt
- Door het gewicht kan uw rollator gaan kantelen
- Vervoer uw tas of lichte boodschappen in het mandje voorop uw rollator

4. Met rollator de stoep af

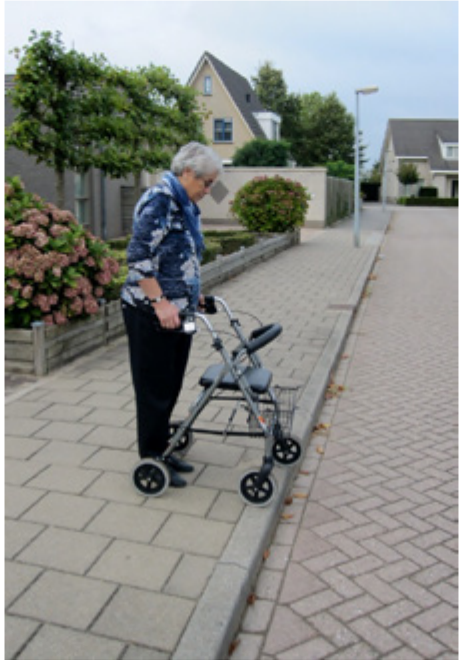

- Ga voor de stoeprand staan
- Rij de rollator met de voorwielen van de stoep
- Doe dit rustig en gecontroleerd

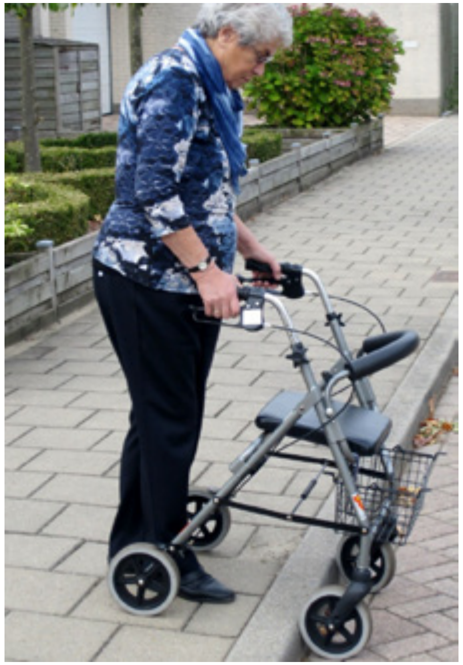

- Knijp in de remmen zodra de voorwielen van de stoep af zijn

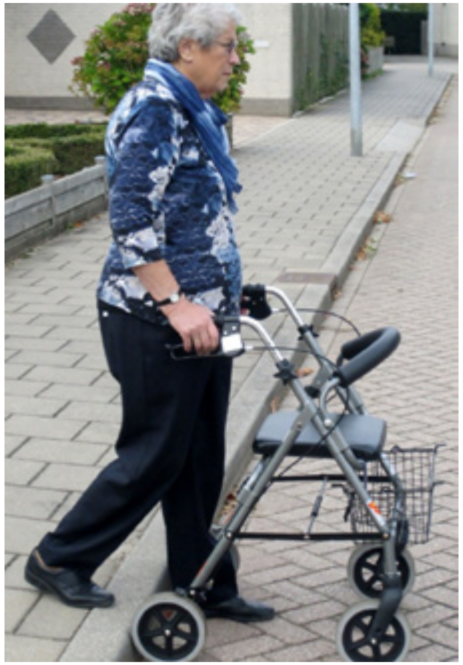

- Laat de remmen weer wat los en rij de rollator in zijn geheel van de stoep af
- Loop mee tussen de wielen

5. Met rollator de stoep op

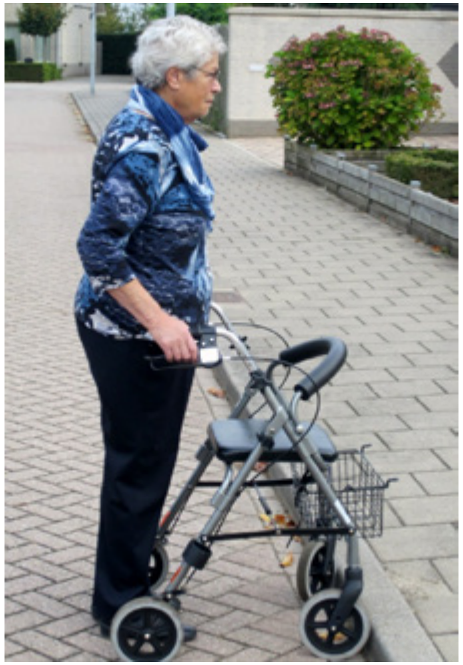

• Loop met de rollator tegen de stoeprand aan

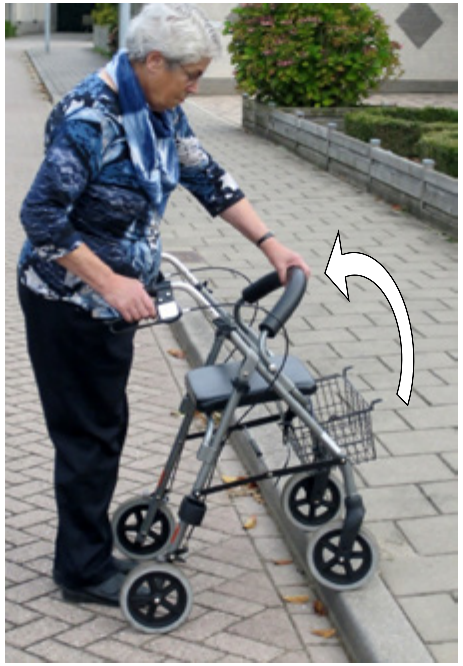

• Kiep met één hand de rollator met de zitbeugel naar u toe zodat de voorwielen omhoog komen

• Zorg dat u hierbij stevig staat

• Plaats de voorwielen op de stoeprand

Tip:

Gebruik het zitje om uw rollator te kiepen als u geen zitbeugel heeft

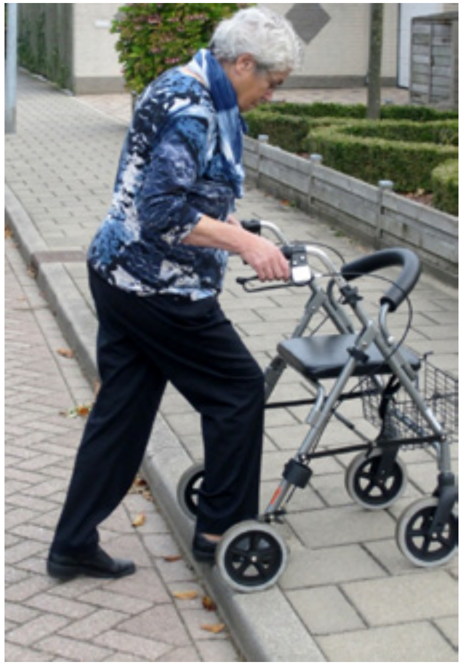

• Rij de rollator op de stoep

• Loop mee tussen de wielen

6. Met rollator een helling af

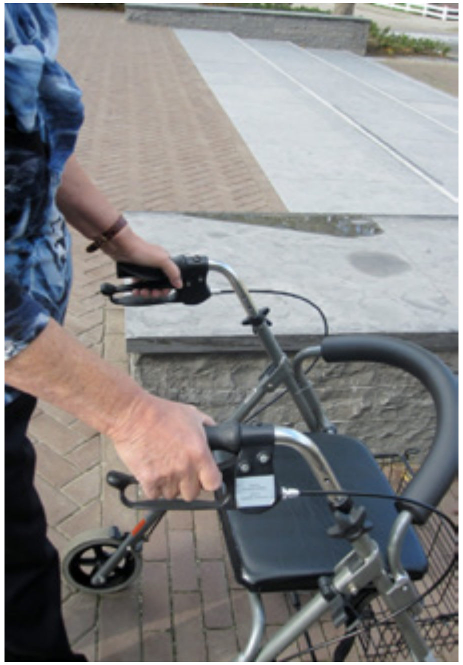

• Knijp in de looprem om bij te remmen

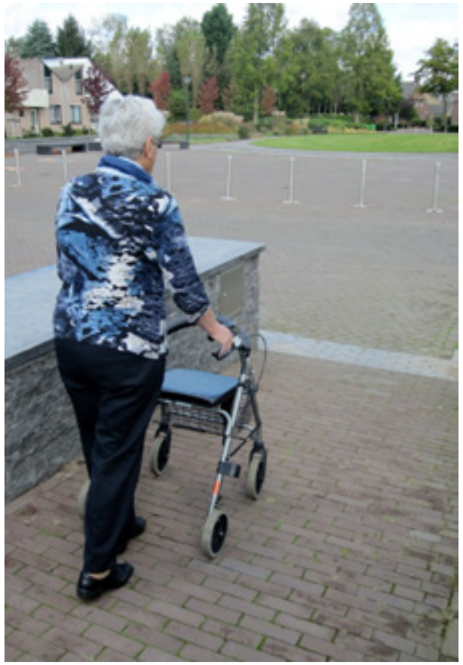

• Loop in rustig tempo de helling af

• Maak kleine stappen en rol over de hak af

7. Met rollator een helling op

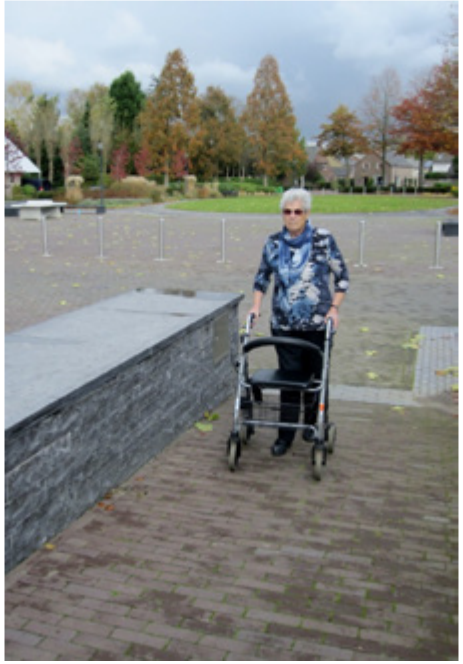

• Loop in rustig tempo de helling op

• Duw hierbij de rollator een stukje vooruit en gebruik indien nodig de looprem

8. Zitten op rollator

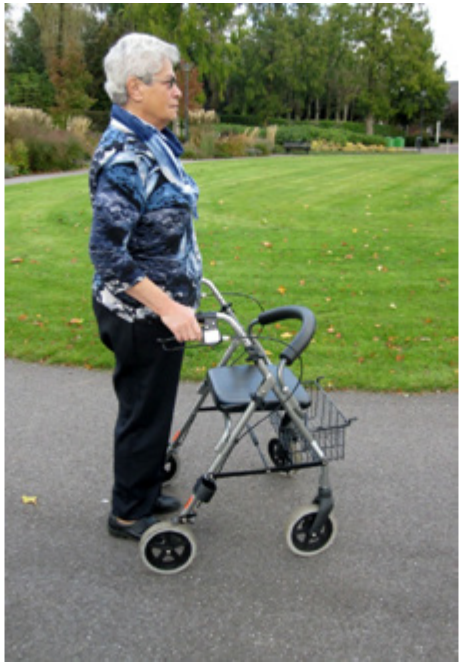

• Veel rollators hebben een zitje. Hier kunt u op zitten als u wilt uitrusten.

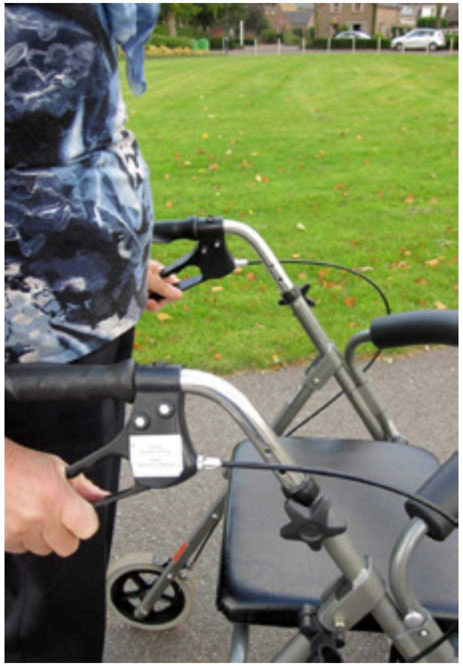

• Gebruik de parkeerrem

• Deze rem zorgt ervoor dat de rollator niet wegrijdt terwijl u zit

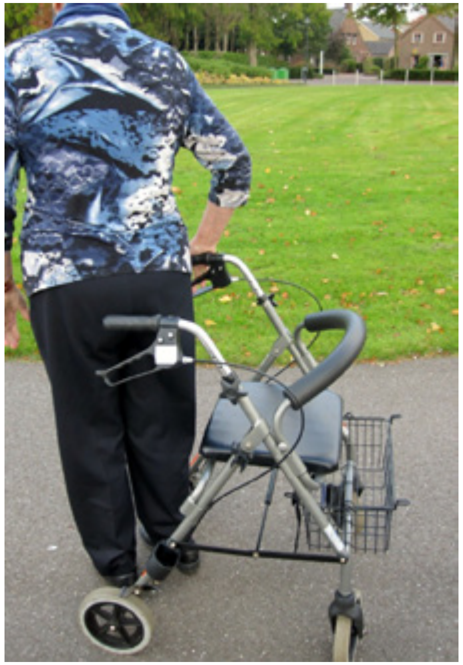

• Draai om

• Neem de handvatten vast

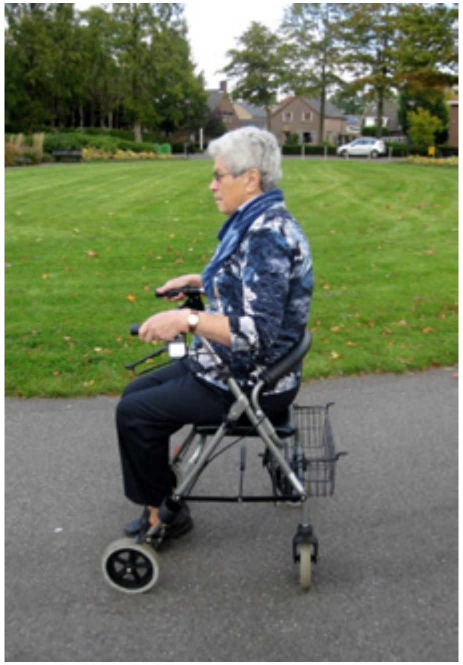

• Ga zitten

9. Opstaan van rollator

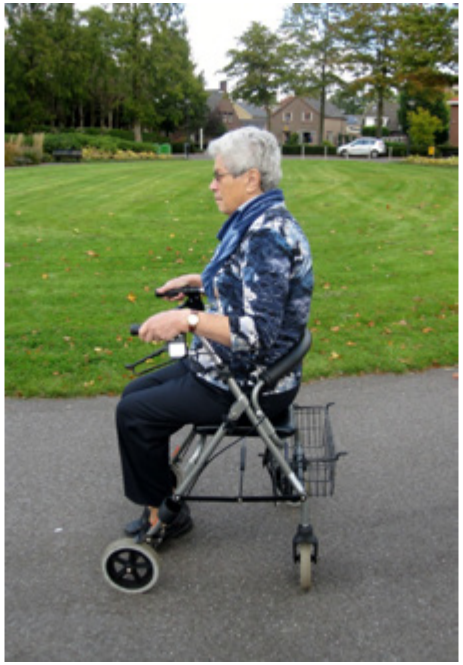

• U wilt weer verder lopen

• Handen op handvatten

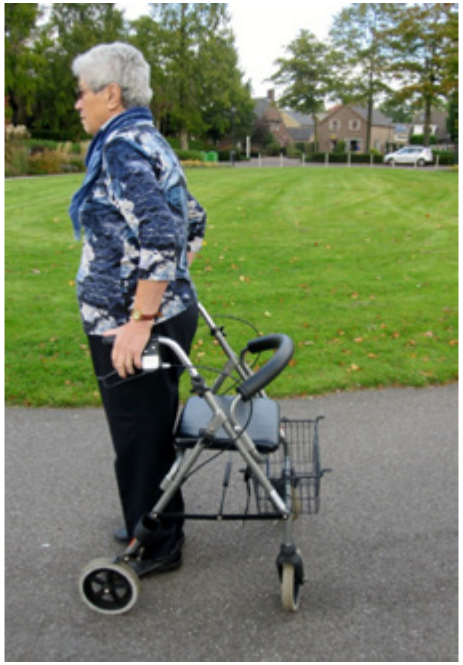

• Sta op

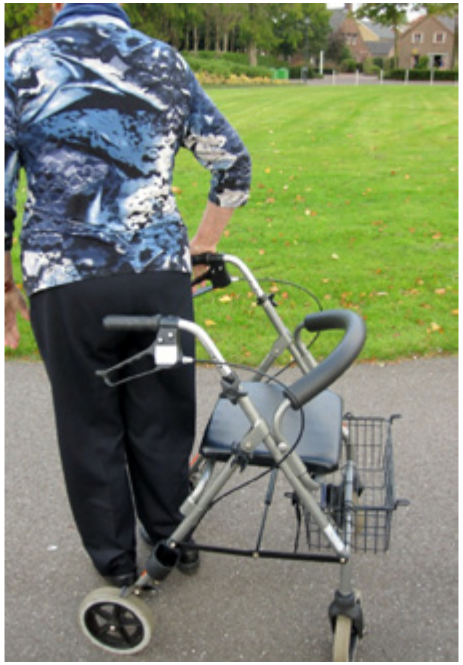

• Draai om

• Pak de handvatten vast

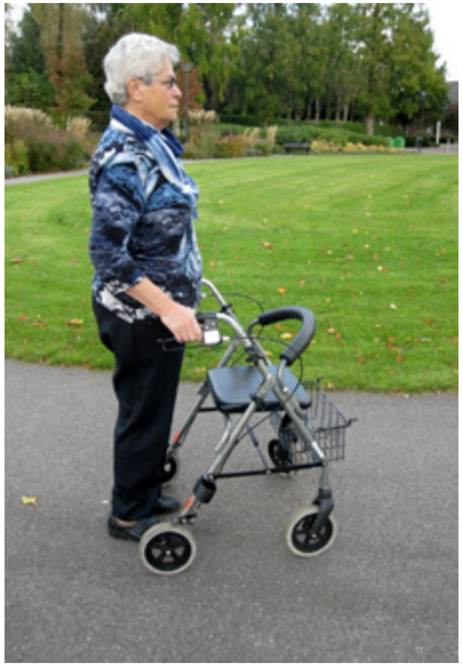

• Eerst stevig staan

• Dan pas parkeerrem er af halen

Instructies Profiel A en B

A

B

Hoe kunt u het oefenprogramma gaan gebruiken?

In het oefenboek zijn de oefeningen voor profiel A en B gebundeld. In de kantlijn van het boek kunt u de kleuren zien en zo bepalen of de oefening bij uw profiel hoort. De blauwe oefeningen horen bij profiel A en de groene bij profiel B. Sommige oefeningen behoren zowel bij profiel A als B, dan ziet u zowel een blauw als groen vlak in de kantlijn.

Profiel A (blauwe oefeningen)

In profiel A gaat u oefenen met het doel om zelfstandig met een loophulpmiddel in uw woning te kunnen lopen zonder begeleiding. Het is aan te bevelen om de oefeningen samen met hulp van uw partner, familie of andere mensen uit uw omgeving te doen.

Profiel B (groene oefeningen)

De oefeningen in profiel B zijn zodanig opgebouwd dat u met meer vertrouwen zelfstandig binnen- en buitenshuis met rollator kunt functioneren. De oefeningen zijn gericht op het verbeteren van uw loopkwaliteit, loopsnelheid en conditie.

Nederlandse Norm voor Gezond Bewegen

Voldoende beweging zorgt ervoor dat u zich fitter gaat voelen. U beweegt voldoende wanneer u op minimaal vijf, maar bij voorkeur alle dagen van de week, 30 minuten matig intensief beweegt. Dit is de Nederlandse Norm voor Gezond Bewegen.

Een matig intensieve beweging is een beweging waarvan u sneller gaat ademhalen. U krijgt het er warm van en uw hart gaat er wat sneller van kloppen.

Als u door beperkingen niet meer aan 30 minuten matig intensief bewegen toekomt, is elke extra hoeveelheid lichaamsbeweging zinvol, ongeacht duur, intensiteit, frequentie of type.

Oefenprogramma

Advies: Wij adviseren u om dagelijks 10 minuten bezig te zijn met oefeningen uit het onderdeel looptraining (zie p. 33 t/m 37).

Daarnaast is het goed om 5 dagen in de week 20 minuten oefeningen uit de andere onderdelen te selecteren. Indien 20 minuten achtereen oefenen te veel voor u is kunt u dit ook opsplitsen in blokjes van bijvoorbeeld 10 minuten.

Begin het programma altijd even met rustige warming up oefeningen uit het onderdeel 'Oefeningen ter verbetering van uw algehele fitheid'. Zie p. 16 t/m 19 voor het samenstellen van een oefenprogramma. Gebruik hierbij de inhoudsopgave op p. 20 t/m 21.

Veiligheid

Voelt u zich niet lekker, heeft u acute pijnklachten, last van duizeligheid of bent u ziek, doe het dan even wat rustiger aan. Raadpleeg eventueel uw huisarts.

Mocht u tijdelijk, door bijvoorbeeld ziekte, niet hebben kunnen oefenen bepaal dan opnieuw welke oefeningen geschikt voor u zijn.

Tip:

Kijk uit met buitenshuis oefenen of wandelen tijdens gladheid in de winter. U zou op dat moment ook kunnen kiezen om hiervoor in de plaats oefeningen binnenshuis te doen.

Profiel A. Hoe kunt u een oefenprogramma samenstellen?

(Gebruik een kopie van het invulformulier op de pagina hiernaast)

Stap 1. Maak een prioriteitenlijst van (voor u) moeilijke activiteiten

Bijvoorbeeld:

1. Opstaan met rollator
2. Lopen met rollator in huis
3. Draaien in bed
4. Gaan zitten
5. Gaan liggen in bed

Stap 2. Selecteer de bijbehorende blauwe oefeningen

Gebruik de inhoudsopgave op p. 20 t/m 21 om de oefeningen die bij de moeilijke activiteit horen te selecteren. Advies: voeg altijd looptraining toe.

Voorbeeld moeilijke activiteit: opstaan met rollator.

- Warming up: oefening 25, 26, 27, 28, 29, 30
- Moeilijke activiteit (opstaan met rollator): oefening 5, 6, 9, 31, 32
- Looptraining: oefening 16, 17, 18, 19

Stap 3. Stel uw oefenprogramma samen

Start met een deel van de oefeningen. Sluit aan bij wat u kunt en/of wat u leuk vindt.

Bijvoorbeeld:

- Warming up: oefening 28, 29, 30
- Opstaan met rollator: oefening 5, 6, 9
- Looptraining: oefening 16, 17

Stap 4. Uitbreiden van oefenprogramma

Als u de oefening goed beheerst kunt u doorgaan met een volgende oefening uit stap 2. Of gebruik de andere oefeningen ter afwisseling.

Stap 5. Maak een nieuw oefenprogramma

Neem nu de volgende moeilijke activiteit van uw prioriteitenlijst en herhaal stap 2, 3 en 4 op een nieuw formulier. Herhaal ook regelmatig oefeningen uit het voorgaande oefenprogramma.

Oefenprogramma A

Schrijf niet
op deze pagina!

Stap 1. Maak een prioriteitenlijst van moeilijke activiteiten

1. ....
2. ....
3. ....
4. ....
5. ....

Stap 2. Selecteer de bijbehorende blauwe oefeningen

Moeilijke activiteit: ....

- Warming up: oefening ....
- Moeilijke activiteit: oefening ....
- Looptraining: oefening ....

Stap 3. Stel uw oefenprogramma samen

- Warming up: oefening ....
- Moeilijke activiteit:oefening ....
- Looptraining: oefening ....

Stap 4. Uitbreiden van oefenprogramma

- Warming up: oefening ....
- Moeilijke activiteit:oefening ....
- Looptraining: oefening ....

Stap 5. Maak een nieuw oefenprogramma. Selecteer de bijbehorende oefeningen.

Neem nu de volgende moeilijke activiteit van uw prioriteitenlijst en herhaal stap 2, 3 en 4 op een nieuw formulier. Herhaal ook regelmatig oefeningen uit het voorgaande oefenprogramma.

Profiel B. Hoe kunt u een oefenprogramma samenstellen?

(Gebruik een kopie van het invulformulier op de pagina hiernaast)

Stap 1. Maak een prioriteitenlijst van (voor u) moeilijke activiteiten

Bijvoorbeeld:

1. Gaan liggen in bed
2. Opstaan uit bed
3. Reiken
4. Binnen lopen met rollator
5. Buiten lopen met rollator

Stap 2. Selecteer de bijbehorende groene oefeningen

Gebruik de inhoudsopgave op p. 20 t/m 21 om de oefeningen die bij de moeilijke activiteit horen te selecteren. Advies: voeg altijd looptraining toe.

Voorbeeld moeilijke activiteit: gaan liggen in bed.

- Warming up: oefening 25, 26, 27, 28, 29, 30
- Moeilijke activiteit (gaan liggen in bed): oefening 1,3, 33, 39, 40
- Looptraining: oefening 16, 17, 18, 19, 20, 21, 22, 23, 24

Stap 3. Stel uw oefenprogramma samen

Start met een deel van de oefeningen. Sluit aan bij wat u kunt en/of wat u leuk vindt.

Bijvoorbeeld:

- Warming up: oefening 25,26, 27
- Gaan liggen in bed: oefening 1, 3, 4
- Looptraining: oefening 16, 17, 18

Stap 4. Uitbreiden van oefenprogramma

Als u de oefening goed beheerst kunt u doorgaan met een volgende oefening uit stap 2. Of gebruik de andere oefeningen ter afwisseling.

Stap 5. Maak een nieuw oefenprogramma

Neem nu de volgende moeilijke activiteit van uw prioriteitenlijst en herhaal stap 2, 3 en 4 op een nieuw formulier. Herhaal ook regelmatig oefeningen uit het voorgaande oefenprogramma.

Oefenprogramma B

Schrijf niet
op deze pagina!

Stap 1. Maak een prioriteitenlijst van moeilijke activiteiten

1. ....
2. ....
3. ....
4. ....
5. ....

Stap 2. Selecteer de bijbehorende groene oefeningen

Moeilijke activiteit: ....

- Warming up: oefening ....
- Moeilijke activiteit: oefening ....
- Looptraining: oefening ....

Stap 3. Stel uw oefenprogramma samen

- Warming up: oefening ....
- Moeilijke activiteit:oefening ....
- Looptraining: oefening ....

Stap 4. Uitbreiden van oefenprogramma

- Warming up: oefening ....
- Moeilijke activiteit:oefening ....
- Looptraining: oefening ....

Stap 5. Maak een nieuw oefenprogramma. Selecteer de bijbehorende oefeningen.

Neem nu de volgende moeilijke activiteit van uw prioriteitenlijst en herhaal stap 2, 3 en 4 op een nieuw formulier. Herhaal ook regelmatig oefeningen uit het voorgaande oefenprogramma.

Inhoudsopgave Profiel A en B

A

B

Oefeningen ter bevordering van het verplaatsen

1. Gaan liggen in bed ..... p. 22
2. Opstaan uit bed ..... p. 23
3. Bruggetje en zijwaarts verplaatsen in bed ..... p. 24
4. In zijlig benen in en uit bed bewegen ..... p. 24
5. Opstaan met rollator ..... p. 25
6. Bovenlichaam voorwaarts bewegen ..... p. 26
7. Gaan zitten met rollator ..... p. 27
8. Rollator- stoelleuning overpakken ..... p. 28
9. Naar voren op puntje van stoel gaan zitten ..... p. 29
10. Goed achterin de stoel gaan zitten ..... p. 29

Oefeningen ter bevordering van het reiken

11. Voorwaarts reiken ..... p. 30
12. Gewicht verplaatsen en zijwaarts met één hand reiken ..... p. 31
13. Zijwaarts met twee handen reiken ..... p. 31
14. Reiken in de hoogte met steun ..... p. 32
15. Reiken in de laagte met steun ..... p. 32

Looptraining

<u>Oefeningen en tips voor bevordering van de loopkwaliteit, loopsnelheid buitenshuis en veilig traplopen</u>

16. Lopen op de plaats ..... p. 33
17. Voorwaarts/achterwaarts lopen ..... p. 34
18. 8-figuur lopen ..... p. 34
19. Lopen met grote stappen ..... p. 34
20. Met rollator deur openen en sluiten ..... p. 35
21. Met rollator een kopje koffie vervoeren ..... p. 35
22. Buitenshuis met rollator samen lopen m.b.v. ritme ..... p. 36
23. Buitenshuis met rollator samen lopen op oneffen terrein ..... p. 36
24. Traplopen onder begeleiding ..... p. 37

Oefeningen ter verbetering van uw algehele fitheid

Warming up en ondersteuning van reiken

25. Armen voorwaarts heffen .... p. 38
26. Armen zijwaarts heffen .... p. 38
27. Hoofd draaien .... p. 38

Warming up en ondersteuning van lenigheid en kracht

28. Strekken knie .... p. 38
29. Knieheffen .... p. 39
30. Tenen naar u toe trekken en wegduwen . p. 39

Ter ondersteuning van kracht in armen

31. Opdrukken stoelleuning .... p. 39

Ter ondersteuning van kracht in bovenbeenspieren, opstaan en gaan zitten in stoel

32. Bovenlichaam voorwaarts bewegen met billen los .... p. 40

Ter ondersteuning van kracht in bil- en beenspieren en zijwaarts verplaatsen in bed

33. Bruggetje .... p. 40

Ter ondersteuning van balans, kracht en lopen

34. Zijwaarts been heffen .... p. 41
35. Achterwaarts bewegen been .... p. 41
36. Kniebuigingen met steun .... p. 42
37. Tenenstand .... p. 42
38. Hakkenstand .... p. 42

Ter ondersteuning van gemakkelijker kunnen verplaatsen in en uit bed

39. Zijlig been heffen .... p. 43
40. Zijlig been heffen en uitstrekken .... p. 43

Ter ondersteuning voor het opbouwen van conditie

41. Conditie: Buitenshuis lopen .... p. 44

A

B

1. Gaan liggen in bed

A

B

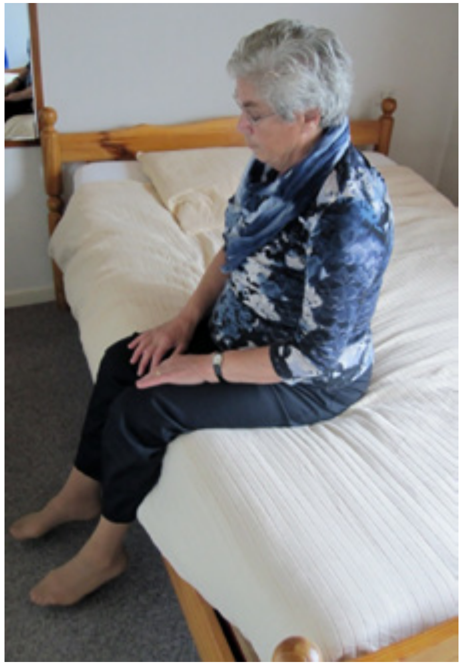

• Zit op bedrand

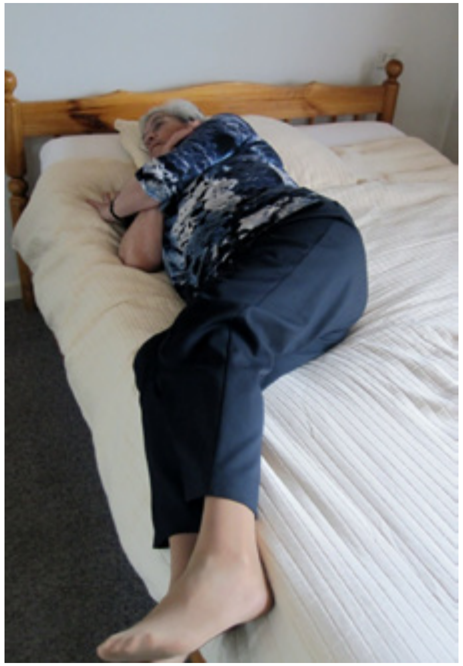

• Ga op de zij liggen

• Neem steun op uw rechterelleboog en linkerhand

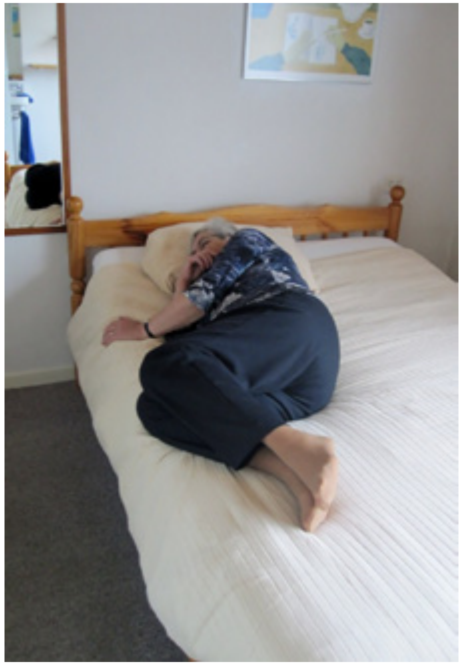

• Til uw voeten in bed

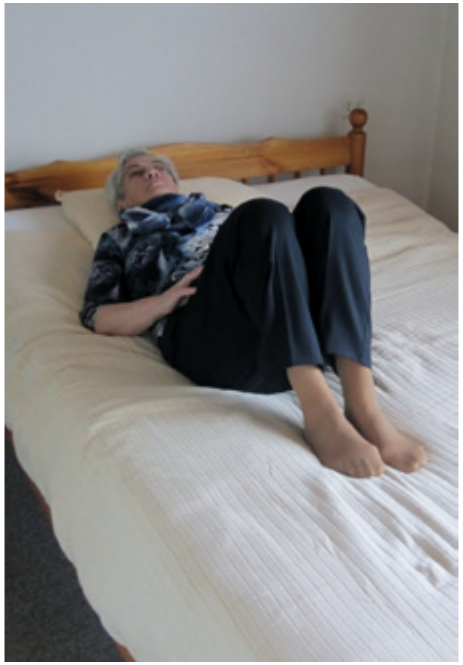

• Draai op de rug

Let op!

Gaat u via de andere kant in bed liggen? Steun op uw linkerelleboog en rechterhand bij het gaan liggen.

2. Opstaan uit bed

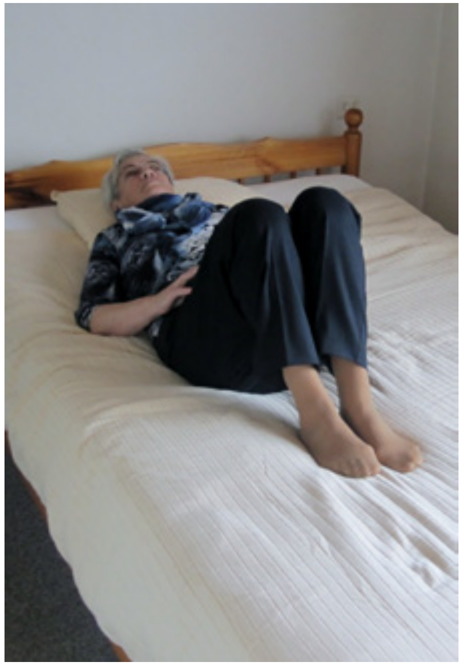

• Plaats uw voeten plat op het bed

• De knieën gebogen

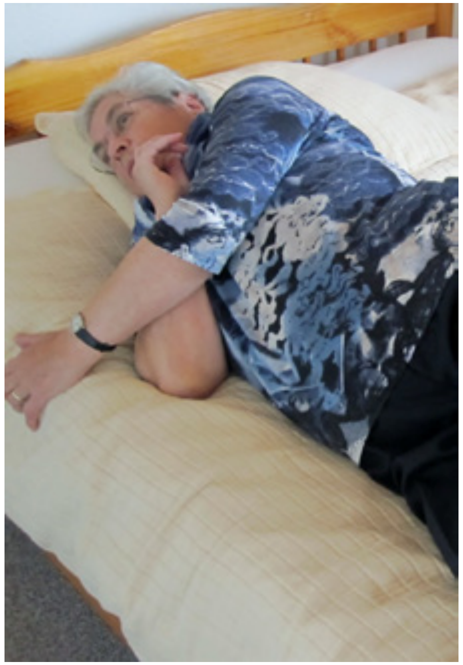

• Breng uw linkerhand naar de bedrand

• En draai op de rechterzij

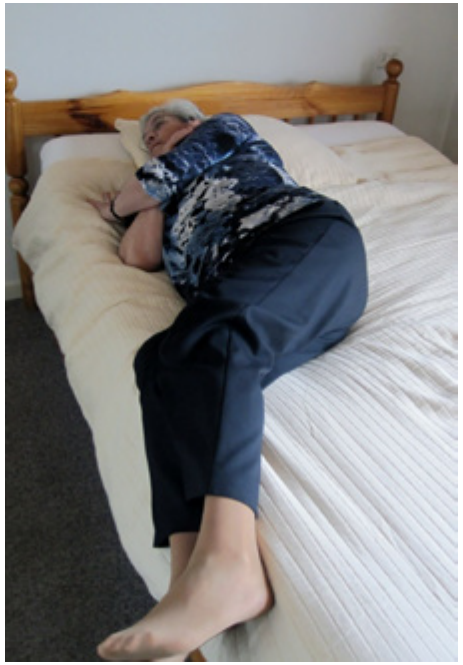

• Neem steun op uw rechterelleboog en linkerhand

• Breng de voeten buiten het bed

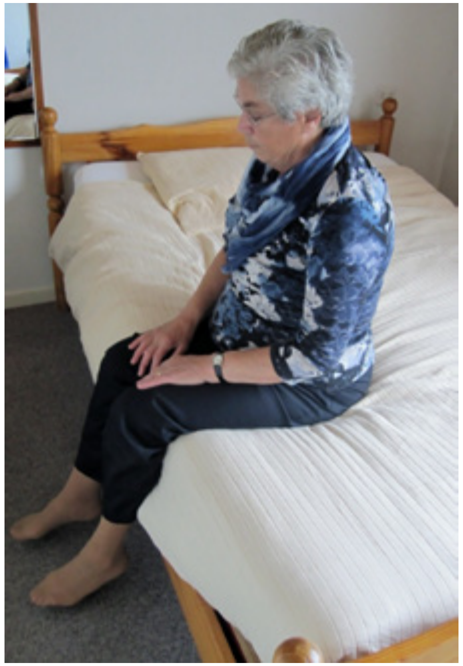

• Kom rustig tot zit

• Blijf even zitten indien u duizelig bent

A

B

Let op!

• Komt u via de andere kant uit bed? Draai op de linkerzij door uw rechterhand naar de bedrand te brengen. Steun op uw linkerelleboog en rechterhand bij het tot zit komen.

• Controleer uw afstand tot de rand van het bed door uw armen ter hoogte van uw schouder zijwaarts te plaatsen. De elleboog mag niet over de rand van het bed komen. Zo ja, doe eerst oefening 3 naar links om draairuimte te creëren.

A

B

3. Bruggetje en zijwaarts verplaatsen in bed

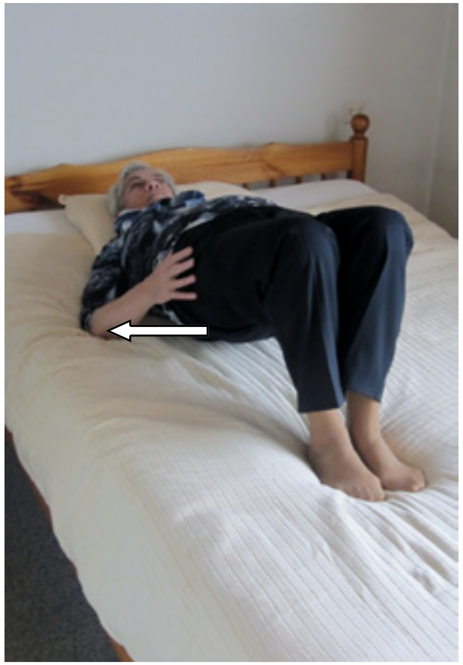

Tip:

Oefen het zijwaarts verplaatsen naar beide kanten. Het zijwaarts verplaatsen is belangrijk om voldoende ruimte te creëren voor het omdraaien.

- Plaats uw voeten plat op bed
- Breng uw hakken zo ver mogelijk naar uw billen
- Til uw billen op (bruggetje)
- Verplaats de billen naar rechts
- Verplaats uw voeten naar rechts
- Verplaats uw bovenlichaam naar rechts

4. In zijlig benen in en uit bed bewegen

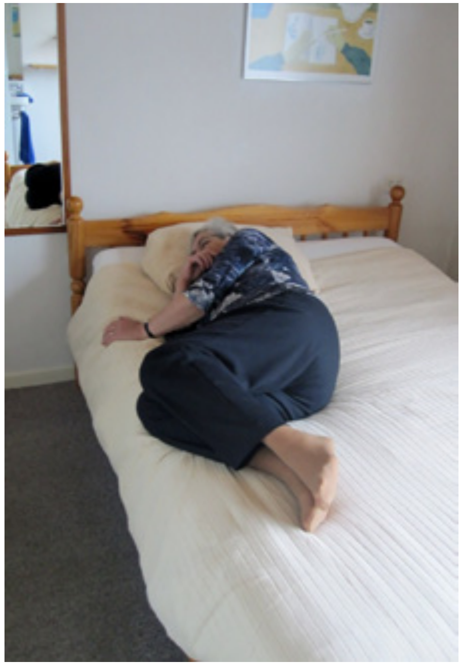

- Zijlig met benen gebogen

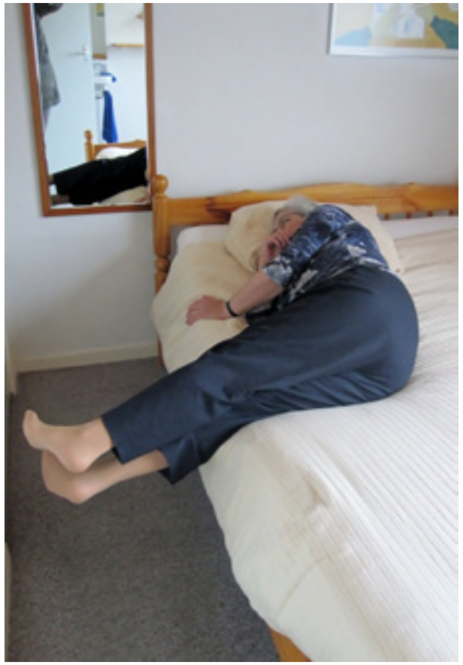

- Strek uw benen buiten de bedrand
- Buig ze vervolgens weer naar de uitgangspositie
- Herhaal 5x

5. Opstaan met rollator

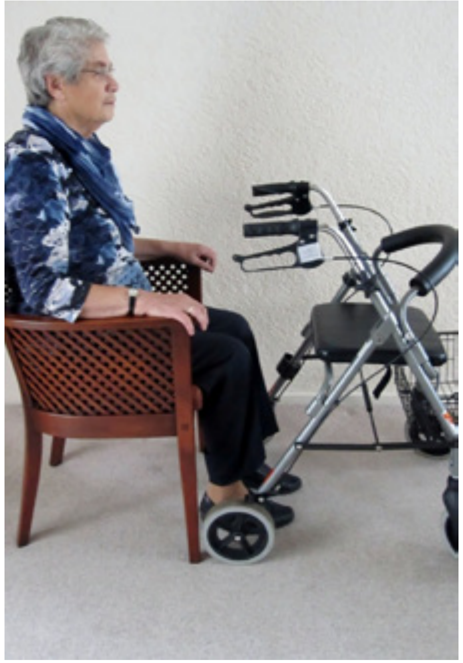

• Zet de voeten stevig op de grond

• Een stukje uit elkaar

• Plaats de handen op de stoelleuning

• Schuif eventueel iets naar voren in de stoel

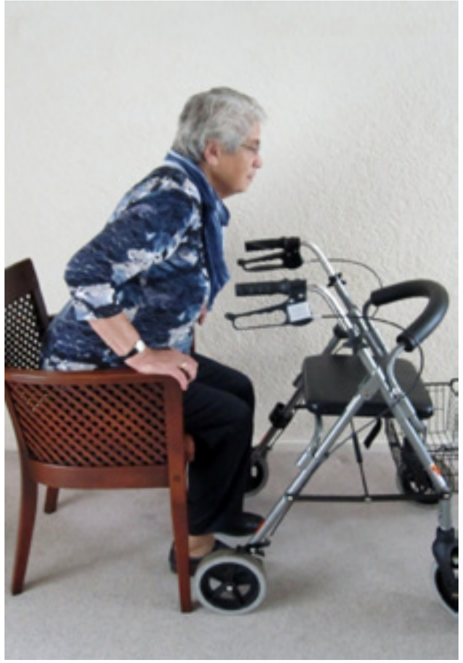

• Breng het bovenlichaam naar voren

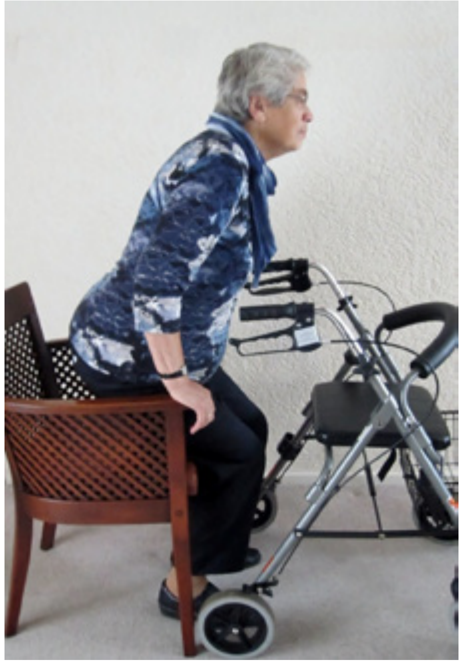

• Strek de benen

• Breng het bovenlichaam omhoog

• Gebruik de handen

• Sta op zoals een vliegtuig opstijgt en niet zoals een raket

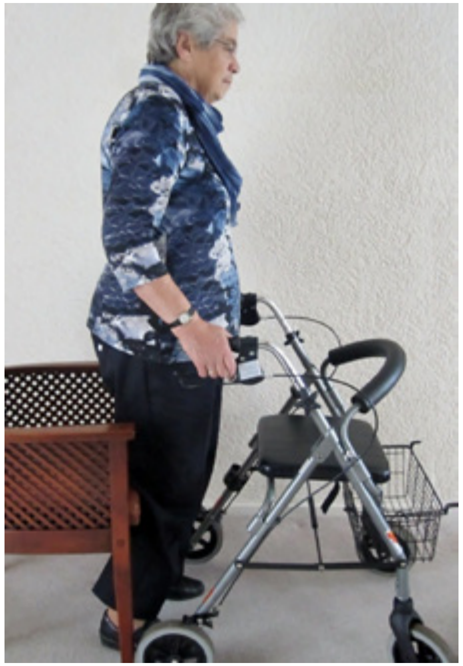

• Ga rustig staan en pak de rollator vast

• Zorg dat u eerst goed in balans bent voor u verder gaat

Tip:

Doe ook oefening 6 'Bovenlichaam voorwaarts bewegen'

6. Bovenlichaam voorwaarts bewegen

A

B

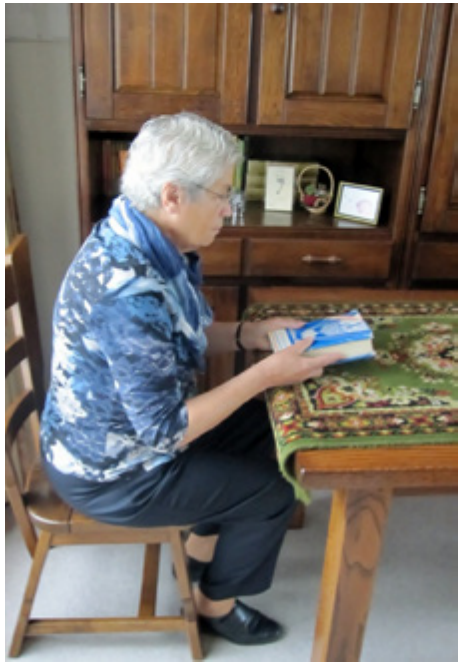

- Zet de voeten stevig op de grond
- Neem een boek in beide handen
- Steun met onderarmen op de tafel

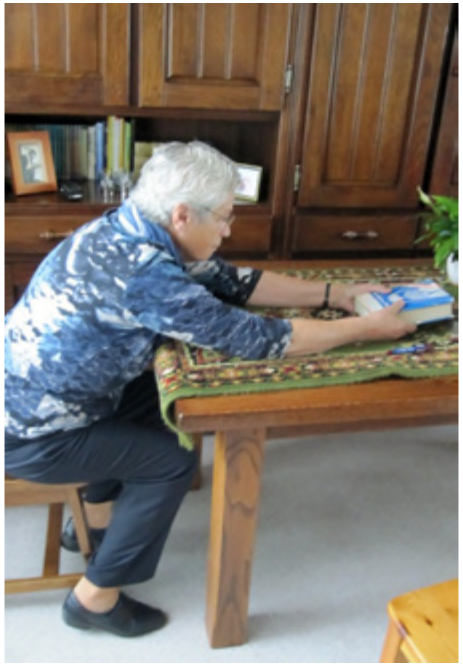

- Breng het boek naar voren
- Schuif hierbij met de armen over de tafel
- Ga weer terug naar de uitgangspositie
- Herhaal 5x

Tip:

Maak de oefening moeilijker door niet te steunen op de tafel

7. Gaan zitten met rollator

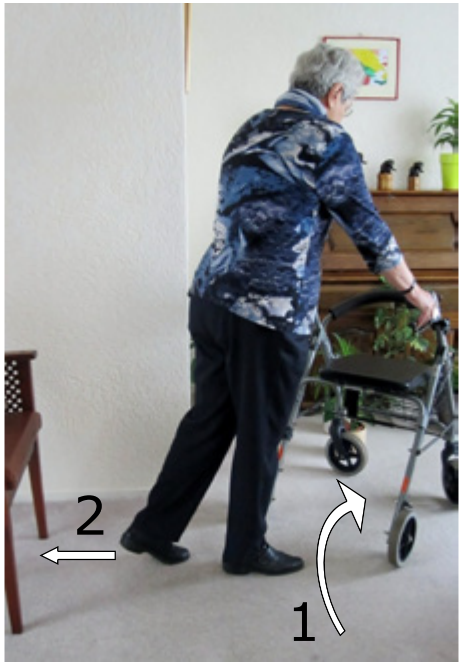

• Loop met rollator naar de stoel

• Draai met een ruime bocht voor de stoel

• Loop achterwaarts naar de stoel

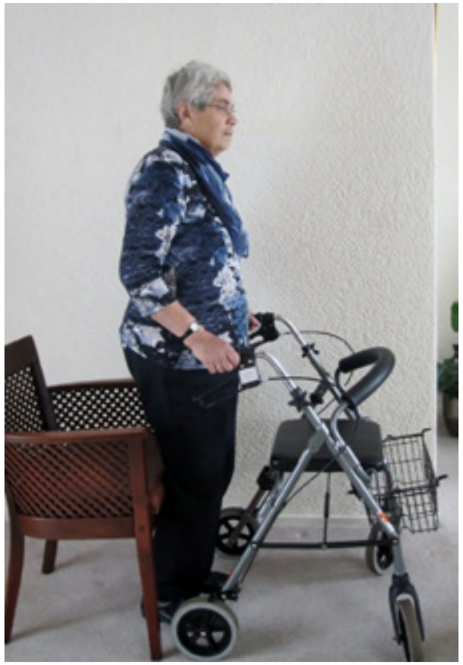

• Tot de knieholten de rand van de stoelzitting raken

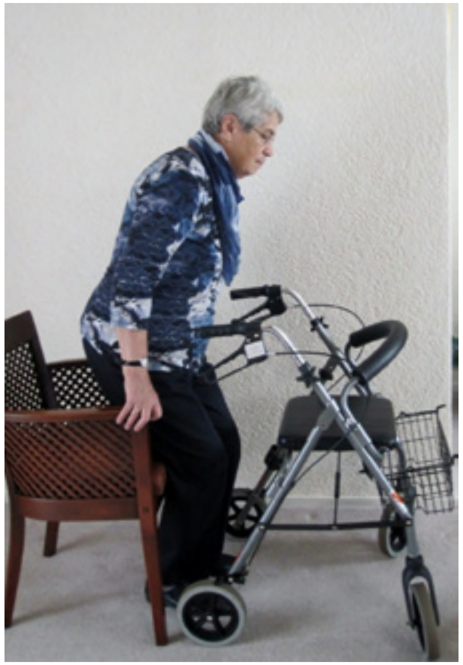

• Pak met beide handen de stoelleuning vast

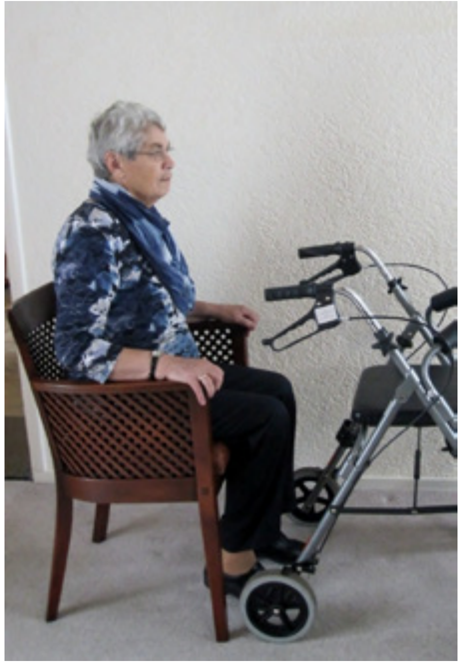

• Ga rustig zitten (niet laten vallen in stoel)

A

B

8. Rollator-stoelleuning overpakken

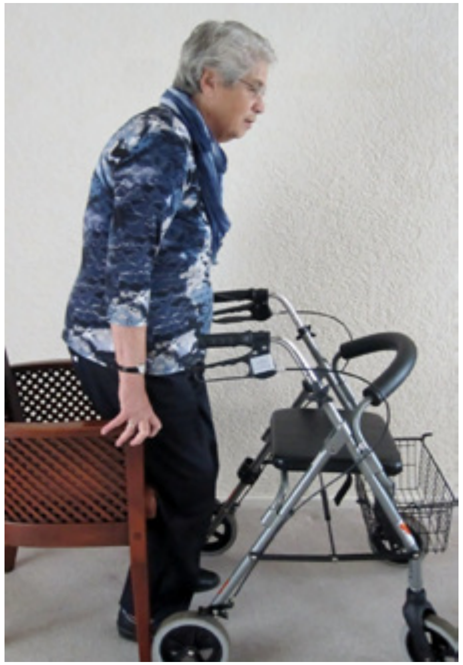

- Ga staan

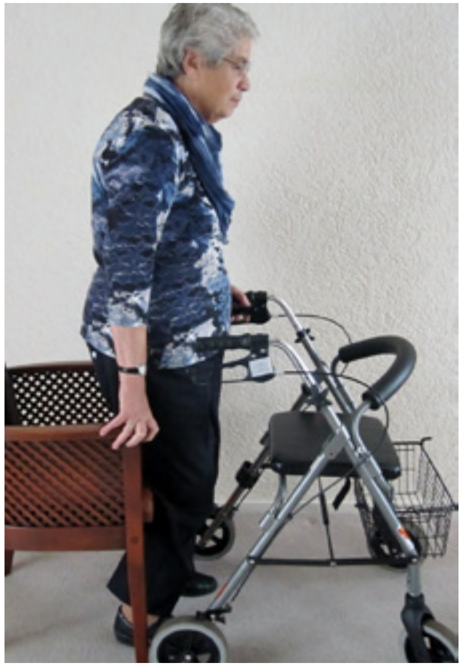

- De rechterhand heeft de stoelleuning vast
- Terwijl u met de linkerhand de rollator vastpakt

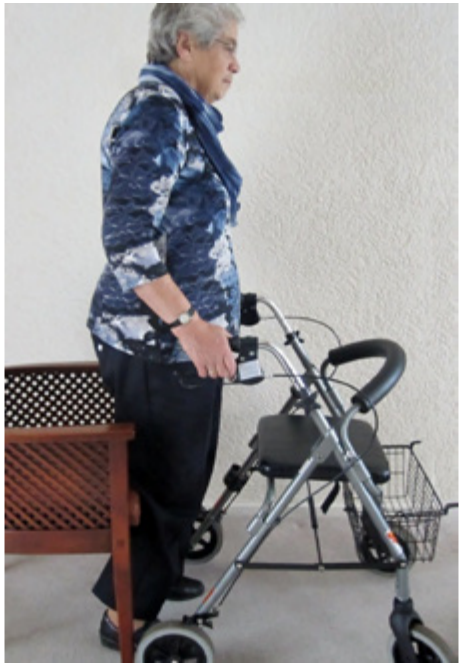

- Beide handen op rollator
- Ga stevig staan
- Herhaal de oefening 1 tot 5x door afwisselend de rollator en stoelleuning vast te pakken

9. Naar voren op puntje van stoel gaan zitten

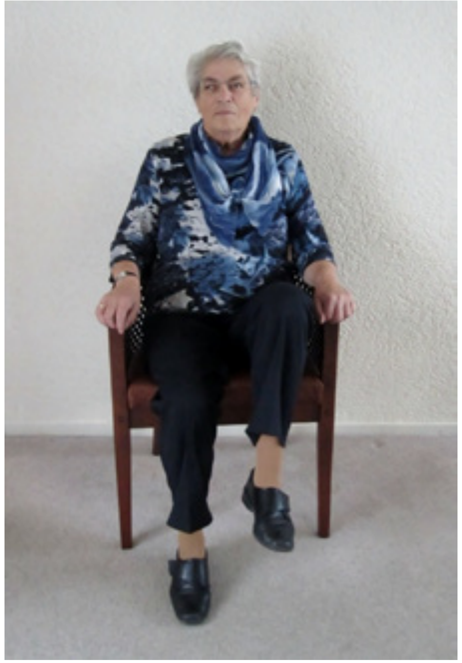

- Til de linkerknie op
- U mag de voorvoet op de grond laten staan

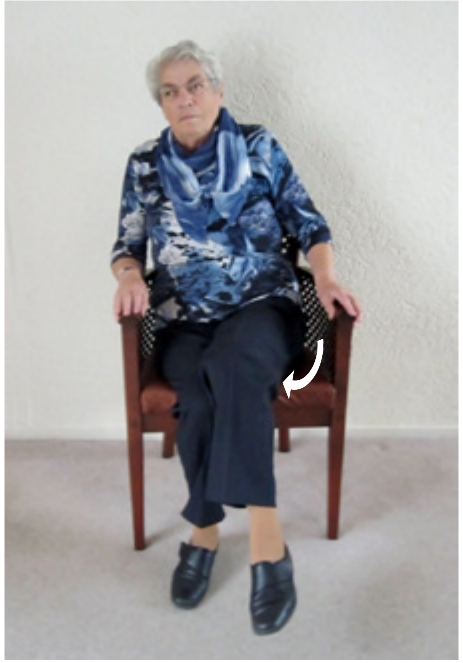

- Til de linkerbil omhoog en verplaats naar voren
- Probeer goed rechtop te blijven zitten
- Herhaal de oefening met uw rechterknie en bil
- Wissel links en rechts af totdat u ver genoeg naar voren zit

10. Goed achterin de stoel gaan zitten

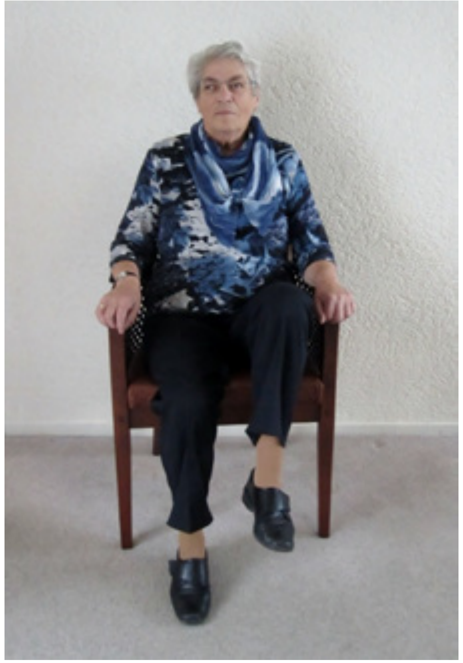

- Til de linkerknie op
- U mag de voorvoet op de grond laten staan

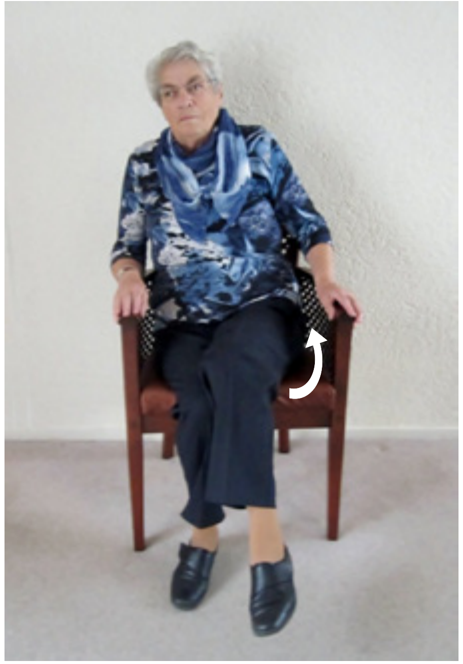

- Til de linkerbil omhoog en verplaats naar achteren
- Probeer goed rechtop te blijven zitten
- Herhaal de oefening met uw rechterknie en bil
- Wissel links en rechts af totdat u goed in de stoel zit

11. Voorwaarts reiken

A

B

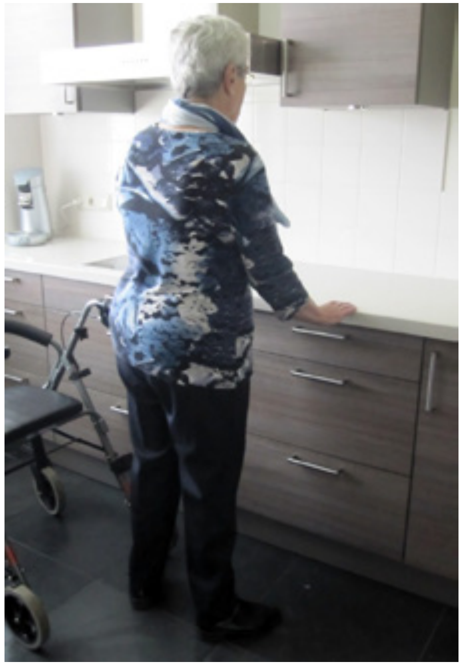

- Ga rechtop staan bij het aanrecht
- Voeten op heupbreedte

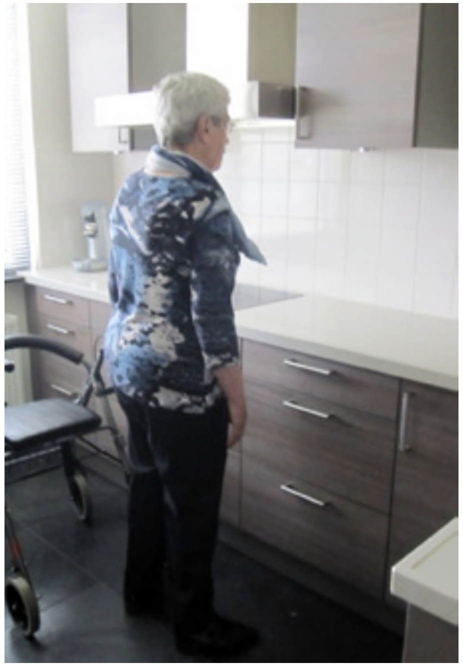

- Probeer 10 sec. zonder steun te staan
- Bouw deze oefening op door langer te staan (10 tot 60 sec.)

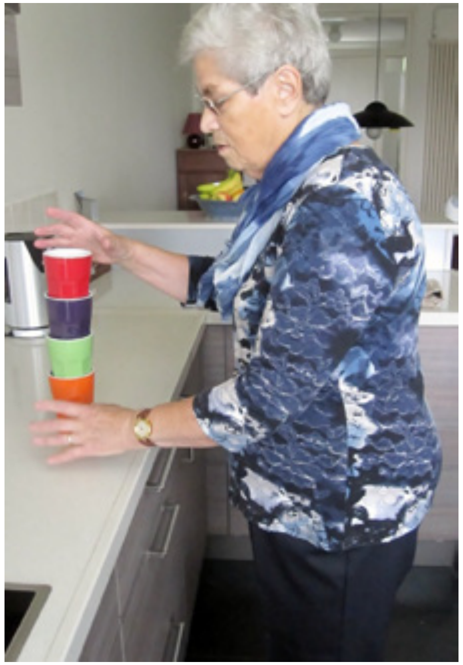

- Sta rechtop en stapel bekers gedurende 30 sec.

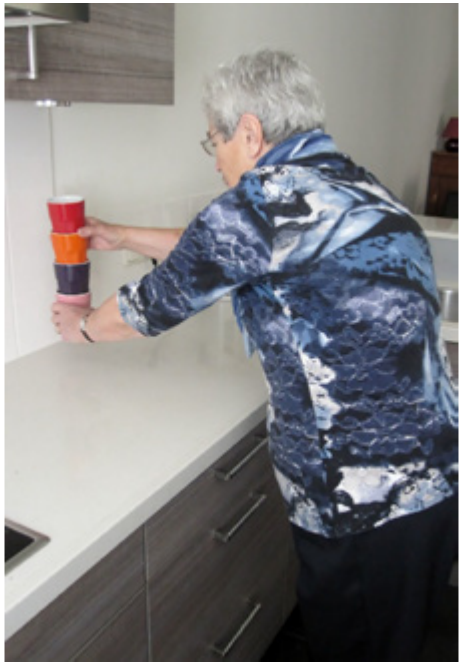

- Verplaats de bekers voorwaarts
- En weer terug
- Herhaal 5x

Tip:

Steun bij instabiliteit met één hand licht op het aanrecht

12. Gewicht verplaatsen en zijwaarts met één hand reiken

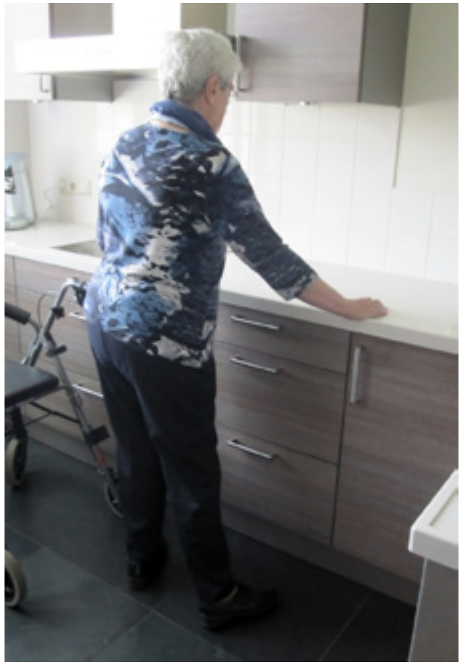

- Rechtop staan bij het aanrecht
- Neem licht steun met handen op het aanrecht
- Voeten op heupbreedte

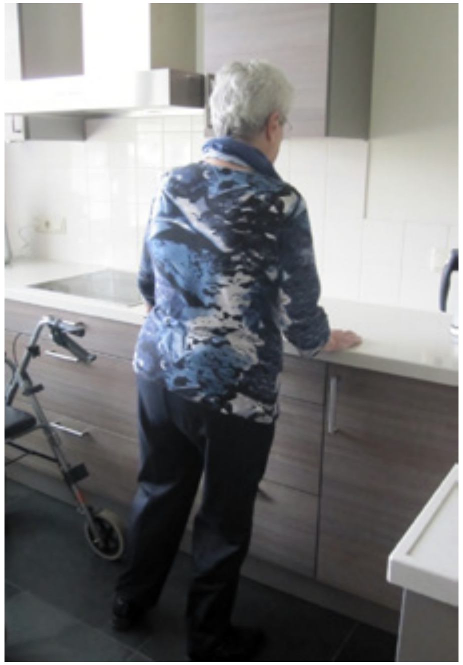

- Verplaats het gewicht heen en weer
- Van ene been op andere been
- Herhaal dit 5x voor beide benen

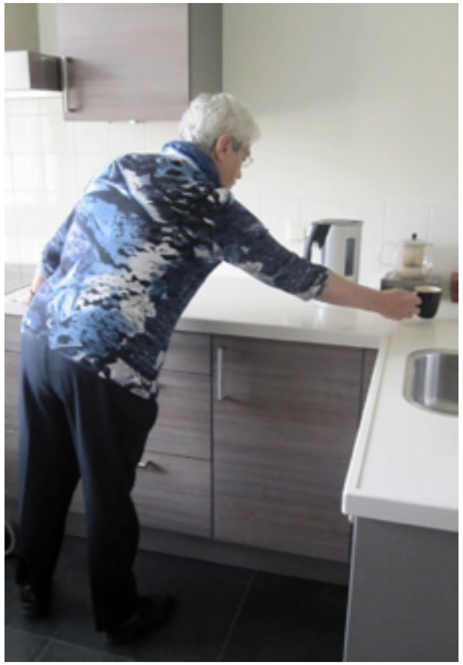

- Neem licht steun met de linkerhand op het aanrecht
- Pak met de rechterhand het kopje door zijwaarts te reiken
- Plaats het kopje afwisselend voor u en zet het weer terug
- Herhaal 5x voor beide kanten

Tip:

Probeer zo ver mogelijk te reiken

13. Zijwaarts met twee handen reiken

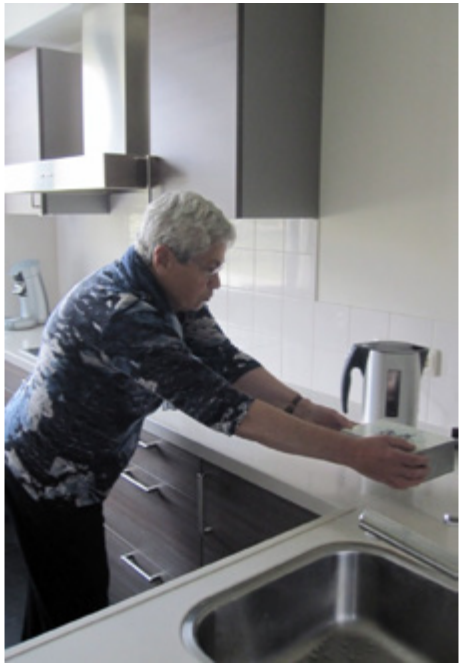

- Rechtop staan bij het aanrecht
- Breng met beide handen een voorwerp zijwaarts naar rechts
- Vervolgens naar links
- Herhaal 5x voor beide kanten

A

B

B

14. Reiken in de hoogte met steun

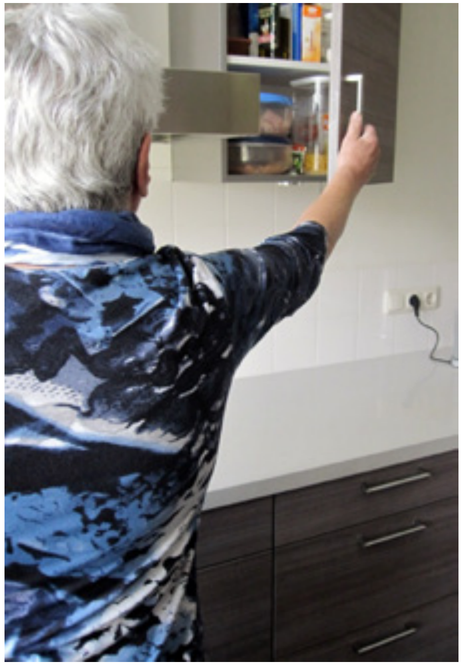

- Rechtop staan bij het aanrecht met de linkerhand gesteund
- Maak met de rechterhand een hoog kastje open
- Pak een voorwerp en plaats het op het aanrecht
- Zet het vervolgens terug in het kastje
- Doe de oefening naar een hoog kastje aan de andere kant
- Herhaal 5x voor beide kanten

Tip:

Probeer zo hoog mogelijk te reiken

15. Reiken in de laagte met steun

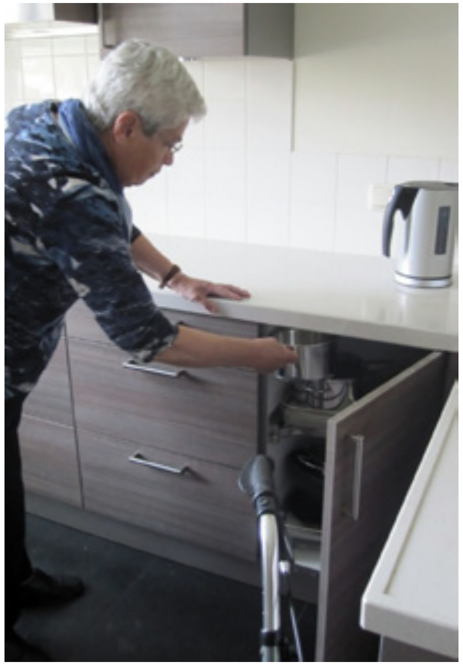

- Steun met uw linkerhand
- Maak met uw rechterhand een laag kastje open
- Pak een voorwerp en plaats het op het aanrecht
- Doe dezelfde oefening naar een laag kastje aan de andere kant

Tip:

U kunt de oefening opbouwen naar 5x voor beide kanten

16. Lopen op de plaats

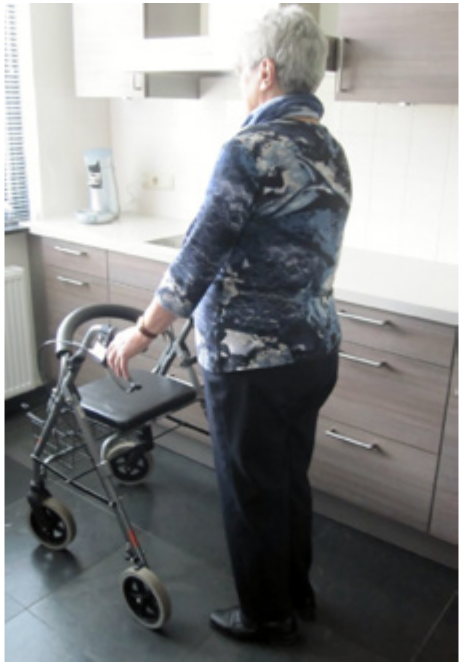

- Ga staan
- Zet de rollator op de parkeerrem
- Pak de rollator vast

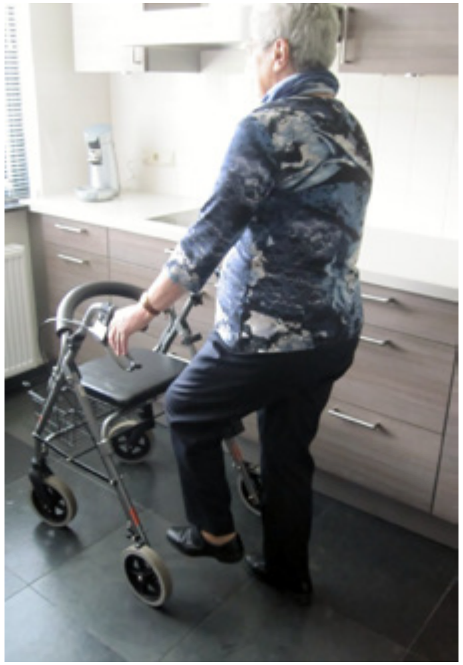

- Til uw linkerbeen op

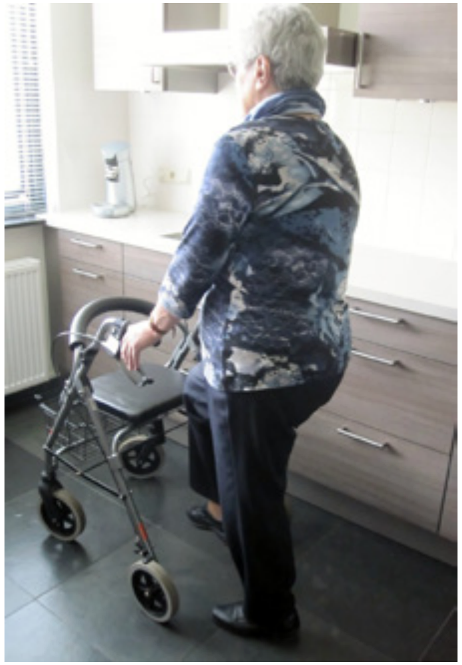

- Til uw rechterbeen op
- Maak afwisselend tien passen op de plaats

A

B

Tips:

- Oefening uitbreiden door één minuut op de plaats te lopen
- Oefening moeilijker maken door knieën hoog op te tillen
- Deze oefening bij het aanrecht uitvoeren indien u geen rollator maar een stok gebruikt. Steun daarbij aan één kant op het aanrecht en met de andere kant op de stok.

A

B

17. Voorwaarts/achterwaarts lopen

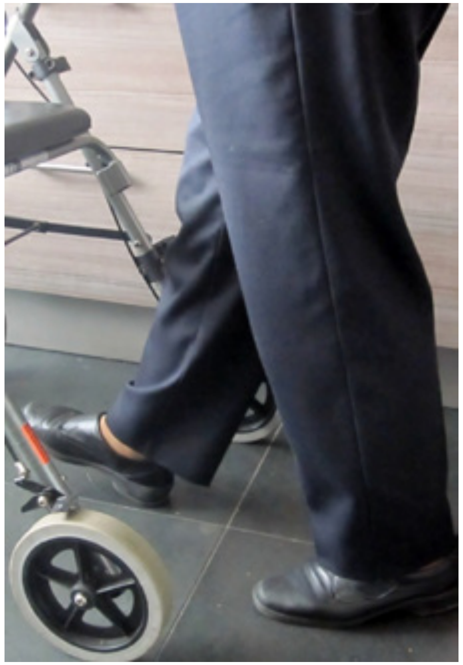

• Loop vijf passen voorwaarts

• Land op uw hak en rol uw voet naar voren af

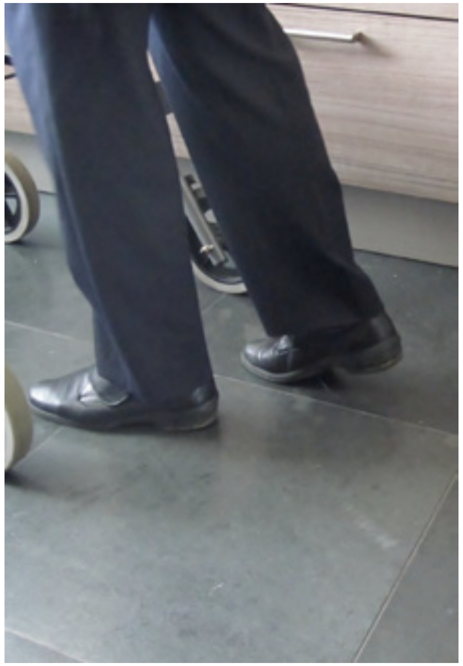

• Loop vijf passen achterwaarts

• Land op uw voorvoet en rol uw voet naar achteren af

• Herhaal het voor- en achterwaarts lopen 5x

18. 8-figuur lopen met steun

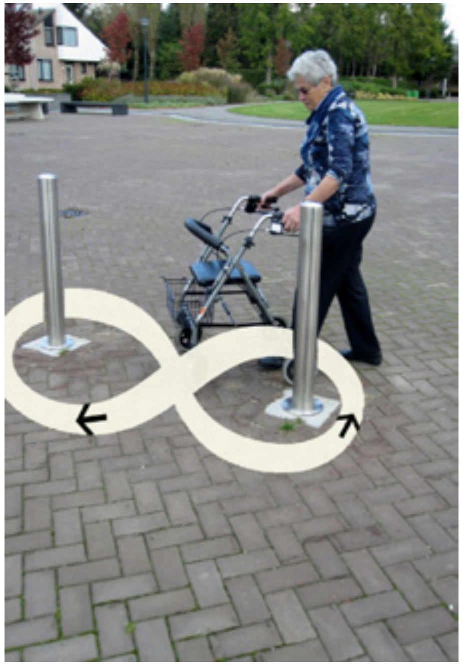

• Loop in een 8-figuur

• Land op uw hak en rol uw voet naar voren af

• Herhaal dit 1x en probeer op te bouwen naar 5x

Tip voor profiel A:

Deze oefening in huis uitvoeren

19. Lopen met grote stappen

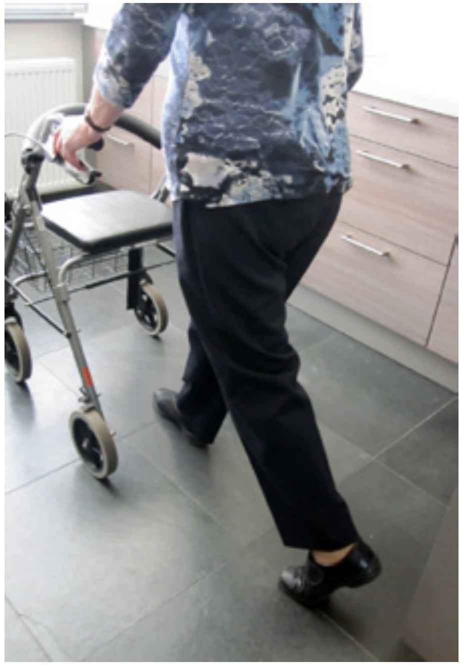

• Loop bijvoorbeeld van woonkamer naar keuken

• Tel het aantal stappen

• Probeer dezelfde route in minder stappen te lopen

• U loopt nu met grotere stappen

• Herhaal 5x de route met grotere stappen

20. Met rollator deur openen en sluiten

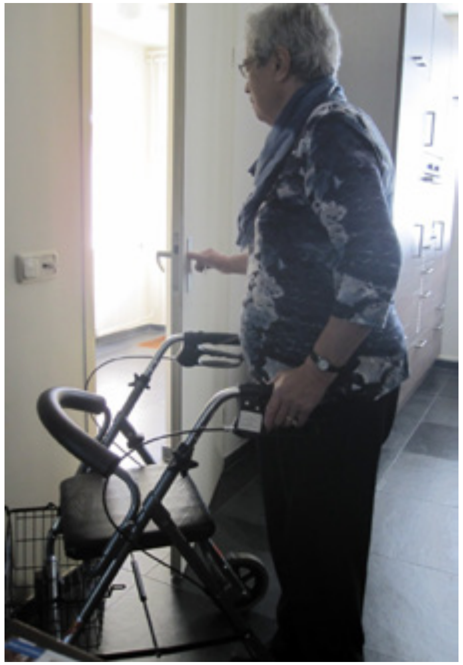

• Loop met rollator naar de deur

• Steun met één hand op de rollator

• Reik met de andere hand naar de deur

• Open de deur

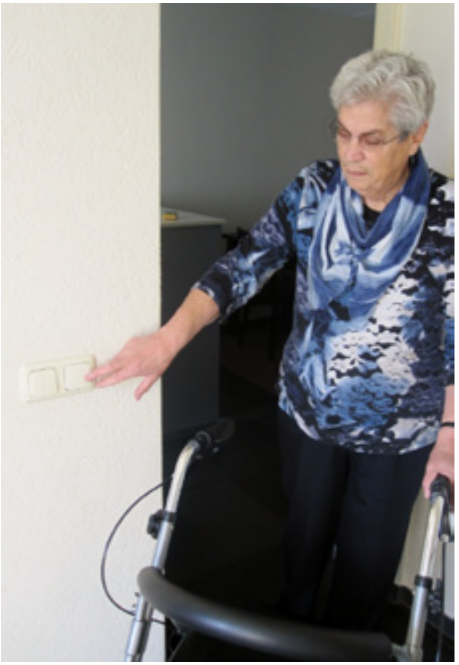

• Loop met twee handen gesteund op de rollator naar het volgende vertrek

• Doe het licht aan zodat de ruimte waar u naartoe gaat goed verlicht is

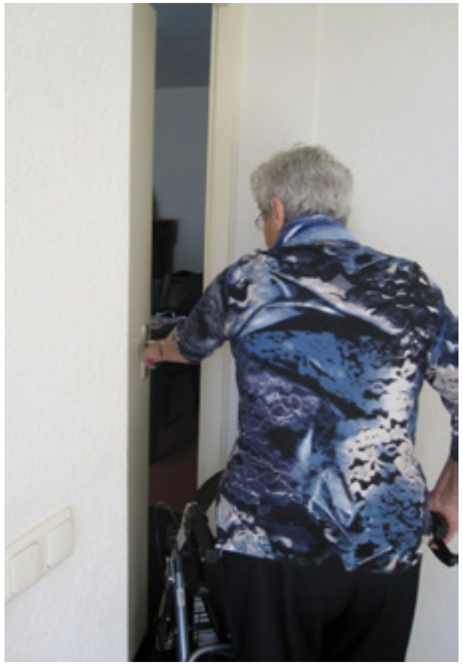

• Draai in rustig tempo met rollator om de as naar de deur

• Blijf steun houden met één hand op de rollator terwijl u met de andere hand de deur sluit

21. Met rollator een kopje koffie vervoeren

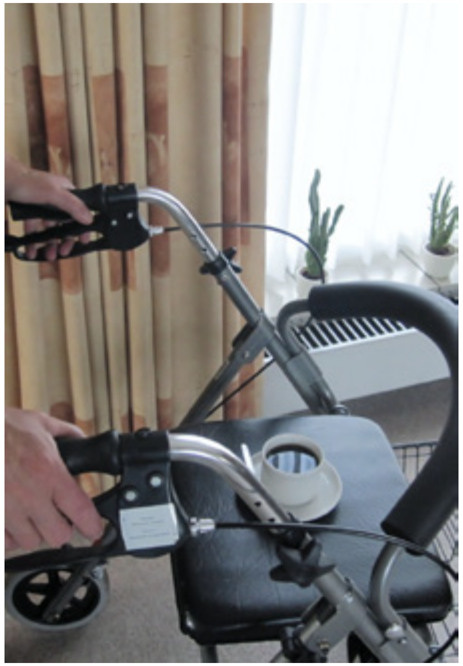

• Plaats een kopje koffie op de rollator

• Hou hierbij de rollater met één hand vast

• Loop een route door uw huis

• Plaats het kopje op de tafel

B

22. Buitenshuis met rollator samen lopen m.b.v. ritme

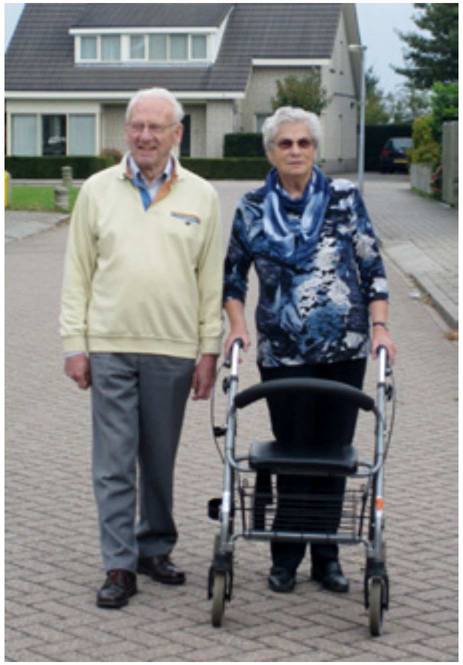

- Land op uw hakken en rol uw voeten verder af
- Loop in een ritme waarbij de begeleidend persoon zich aanpast aan uw tempo
- Maak grote stappen

Tips:

- Loop in de maat door hardop te tellen bijvoorbeeld 1, 2, 1, 2 of benoem links, rechts, links, rechts (marcheren)
- Als u met de rollator draait zorg ervoor dat u een ruime bocht maakt en niet om de as draait

B

23. Buitenshuis met rollator samen lopen op oneffen terrein

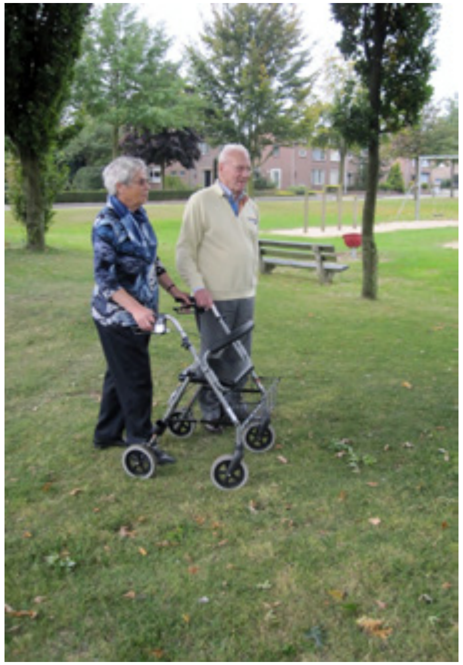

- Zorg dat u uw voeten goed optilt
- Loop in een rustig tempo met volle aandacht

24. Traplopen onder begeleiding

Trap op met steun trapleuning

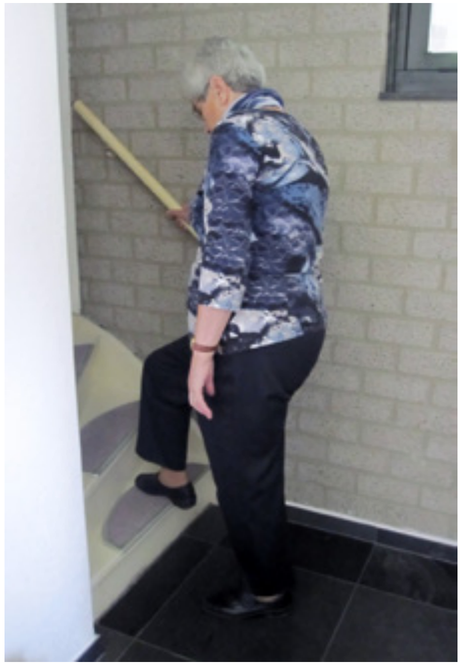

- Pak altijd de trapleuning vast
- Indien mogelijk aan beide kanten
- Plaats uw sterkste been op de eerste traptrede

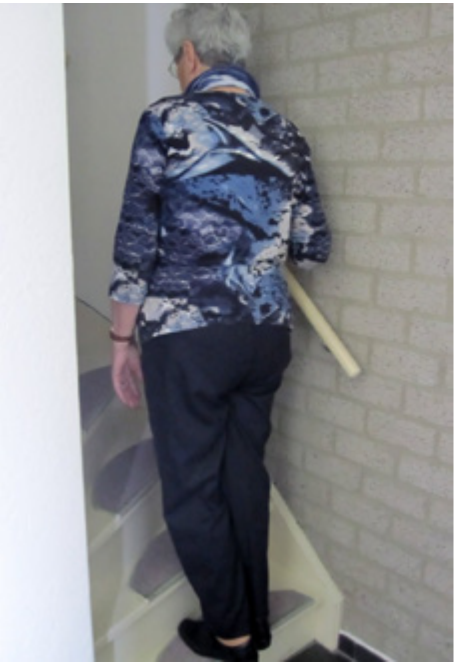

- U kunt nu uw andere been ernaast zetten
- Loop op deze manier in rustig tempo met volle aandacht naar boven

Trap af met steun trapleuning

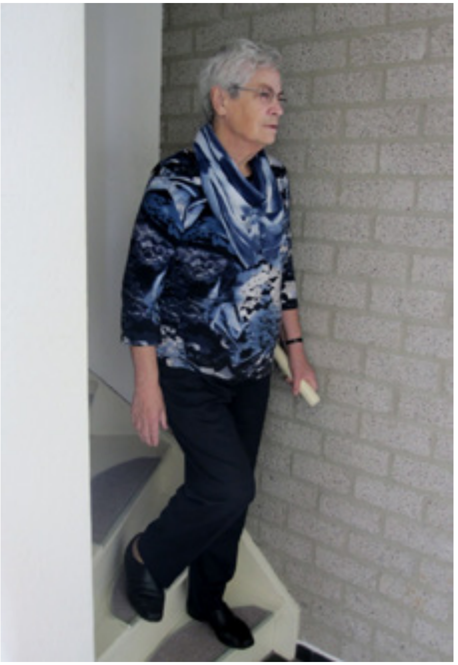

- Pak altijd de trapleuning vast
- Indien mogelijk aan beide kanten
- Plaats eerst uw zwakkere been omlaag
- Zet het sterkere been ernaast

Let op!

- Zorg dat er geen voorwerpen op de trap liggen
- Zorg dat er iemand is die u kan begeleiden
- Bij twijfel niet traplopen
- Indien het goed gaat kunt u ook doorstappen in plaats van uw voet steeds naast de andere te zetten

A

B

25. Armen voorwaarts heffen

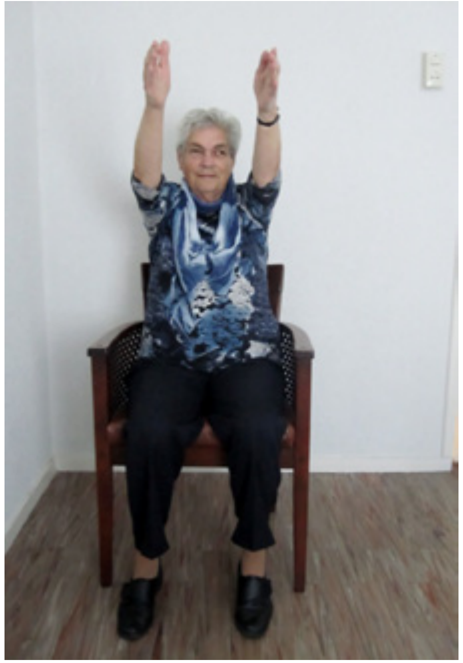

• Breng uw armen in 3 sec. voorwaarts omhoog en adem diep in

• Breng uw armen op de uitademing omlaag in 6 sec.

• Herhaal 5x

26. Armen zijwaarts heffen

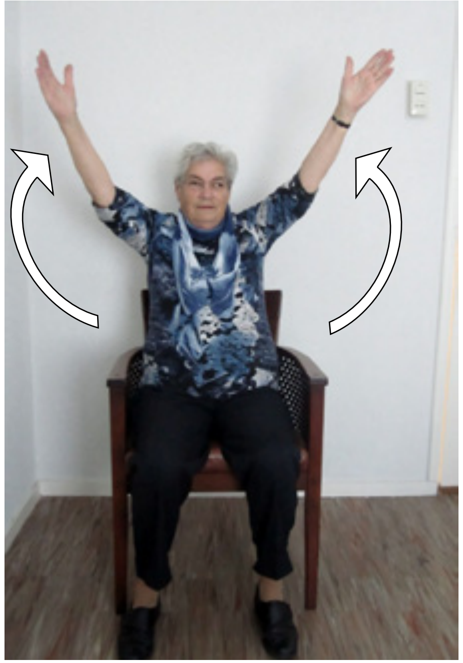

• Breng uw armen in 3 sec. zijwaarts omhoog en adem diep in

• Handpalmen wijzen omhoog naar elkaar toe

• Breng uw armen op de uitademing omlaag in 6 sec.

• Herhaal 5x

27. Hoofd draaien

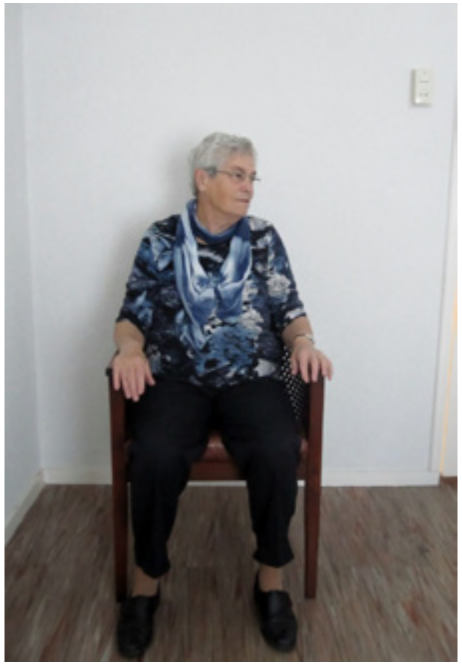

• Draai uw hoofd zo ver mogelijk naar links

• Probeer over uw schouder te kijken

• Doe dit vervolgens ook naar rechts

• Herhaal 5x naar beide kanten

28. Strekken knie

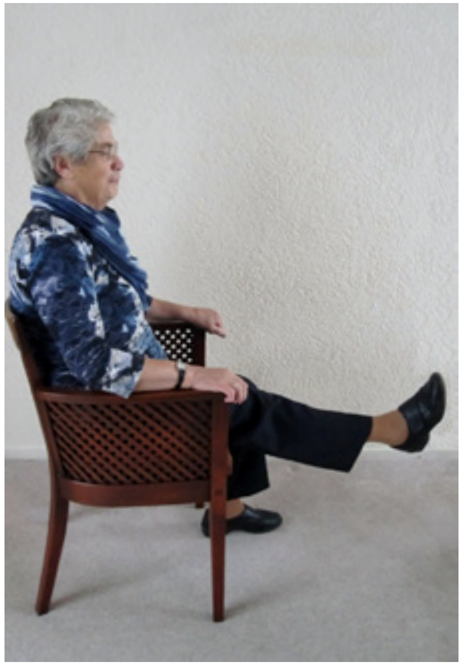

• Strek de rechterknie uit in langzaam tempo (2 sec.)

• Het bovenbeen rust op de stoel

• Buig het rechterbeen in 4 sec. naar de beginpositie

• Herhaal 5x voor beide benen

29. Knieheffen

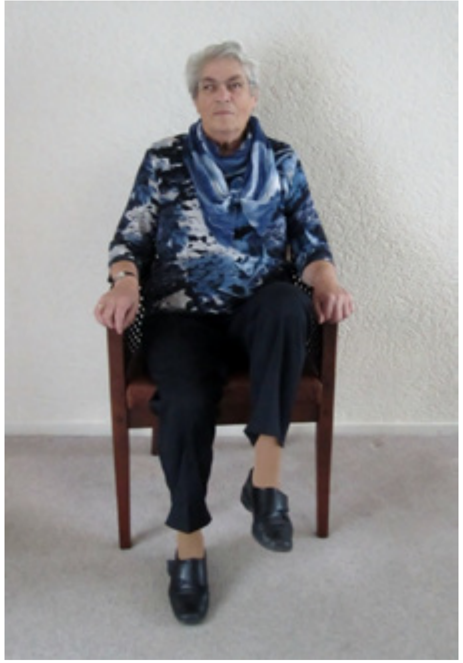

- Til de linkerknie op in 2 sec.
- Breng hem in 4 sec. terug naar de beginpositie
- Herhaal 5x voor beide benen

30. Tenen naar u toe trekken en wegduwen

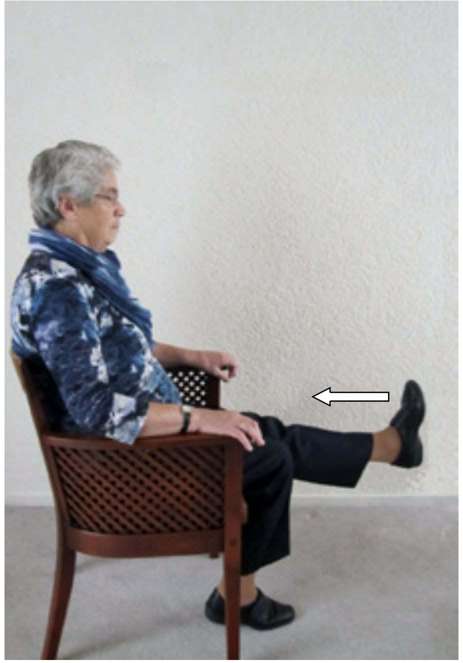

- Strek uw been uit
- Tenen naar u toe trekken
- Hou 2 sec. vast

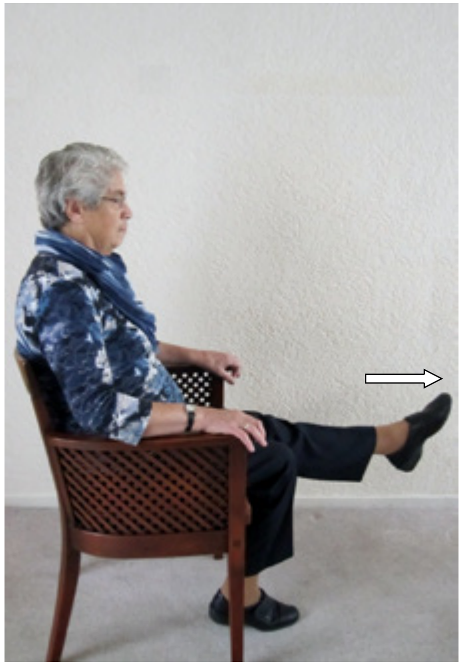

- En afwisselend wegduwen
- Hou 2 sec. vast
- Herhaal 5x keer voor beide voeten

31. Opdrukken stoelleuning

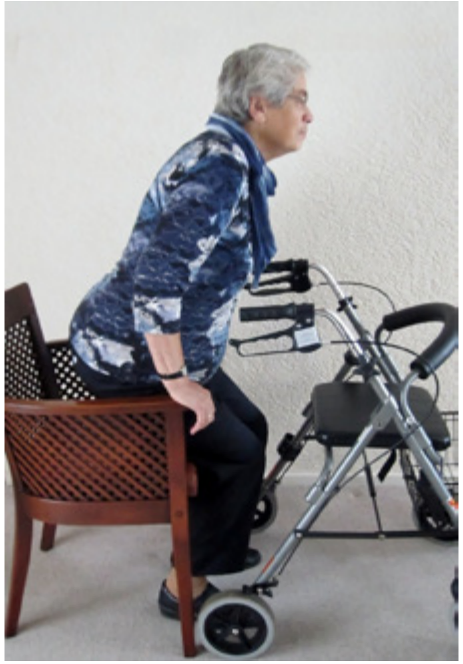

- Druk uw armen op door ze te strekken
- Gebruik hiervoor de stoelleuning
- Neem hierbij zoveel mogelijk gewicht op uw armen

Tip:

Probeer deze oefening op te bouwen naar 5x

A

B

A

B

32. Bovenlichaam voorwaarts bewegen met billen los

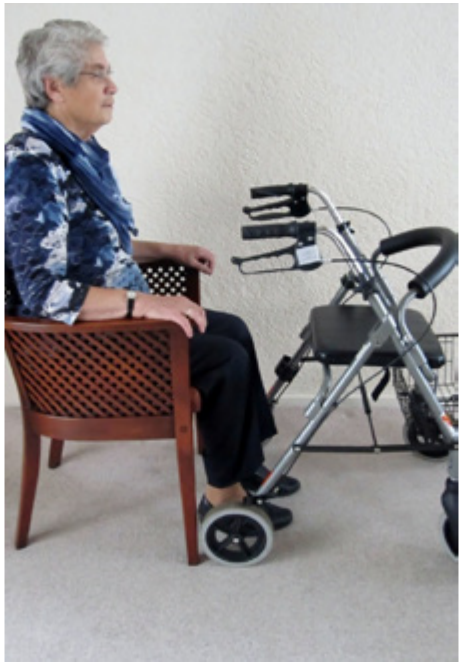

- Zet de voeten stevig op de grond
- Een stukje uit elkaar
- Plaats de handen op de stoelleuning

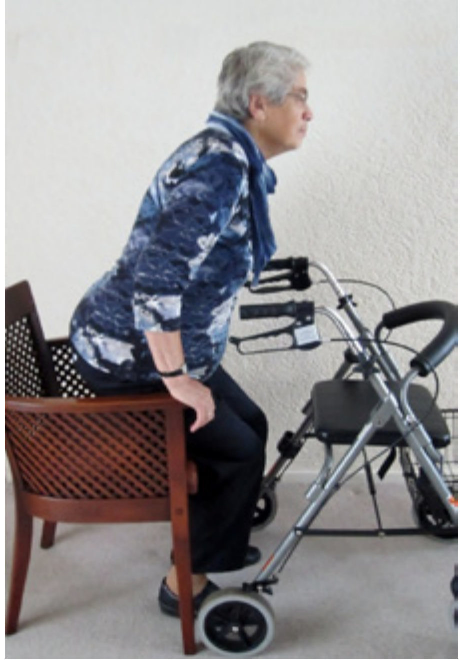

- Breng het bovenlichaam naar voren
- Breng het bovenlichaam een stukje omhoog
- Gebruik de handen
- En ga weer zitten
- Herhaal 5x

33. Bruggetje

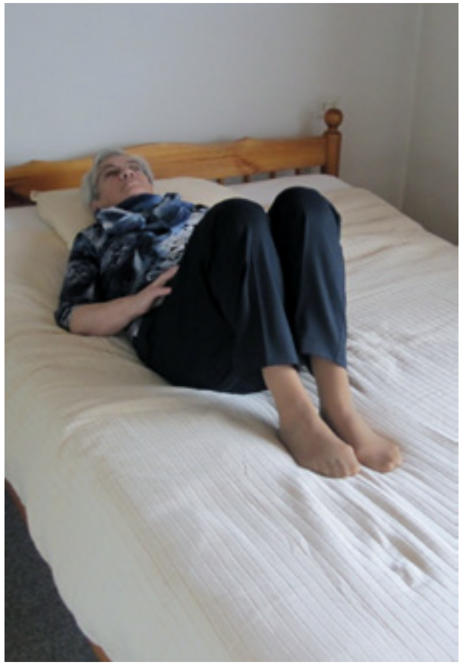

- Plaats uw voeten plat op het bed
- Breng uw hakken zo ver mogelijk naar uw billen

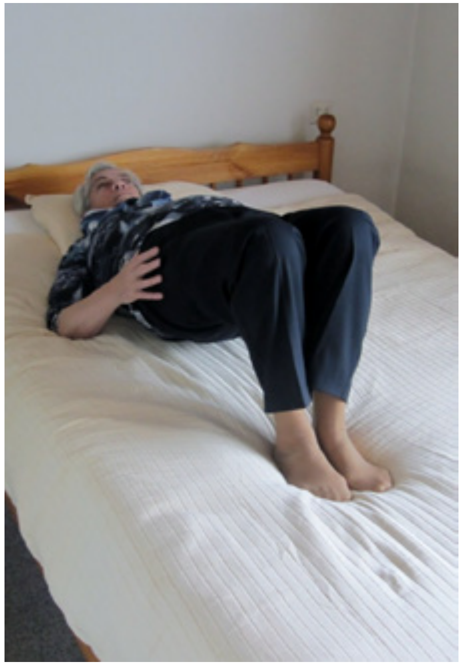

- Til uw billen op (bruggetje)
- Leg de billen weer terug op het bed

Tip:

Probeer de herhalingen op te bouwen naar 5x

34. Zijwaarts been heffen

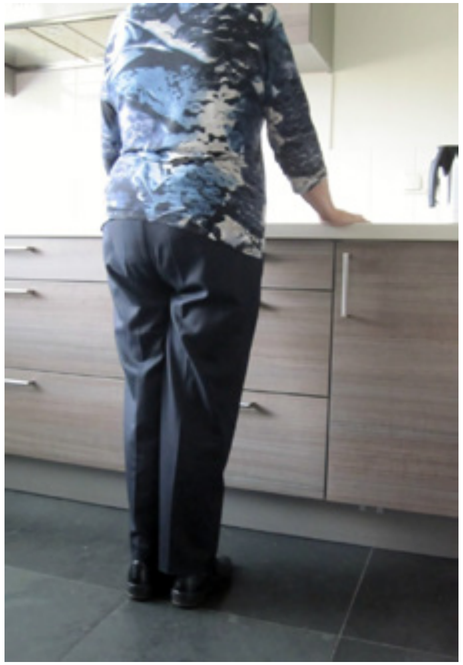

- Rechtop staan
- Handen licht steunend op het aanrecht

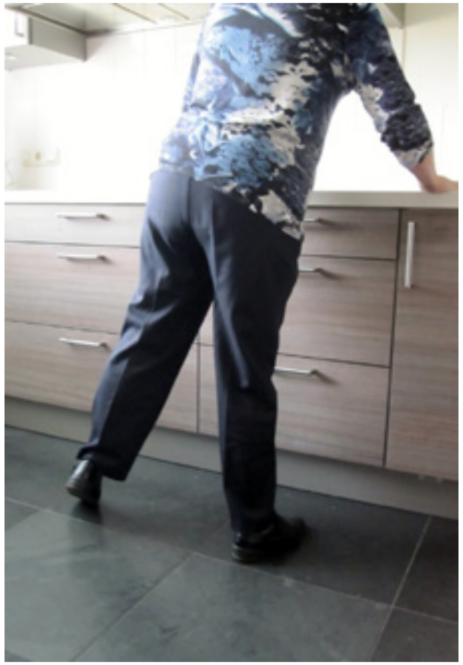

- Beweeg uw linker- en rechterbeen afwisselend zijwaarts
- Raak met uw tenen de vloer
- Herhaal ieder been 5x

Tip:

U kunt deze oefening moeilijker maken door het been te heffen zonder de vloer te raken met uw tenen

35. Achterwaarts bewegen been

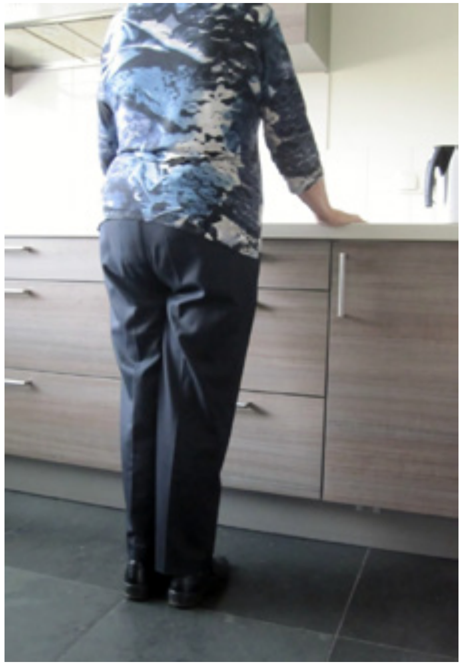

- Rechtop staan
- Handen licht steunend op aanrecht

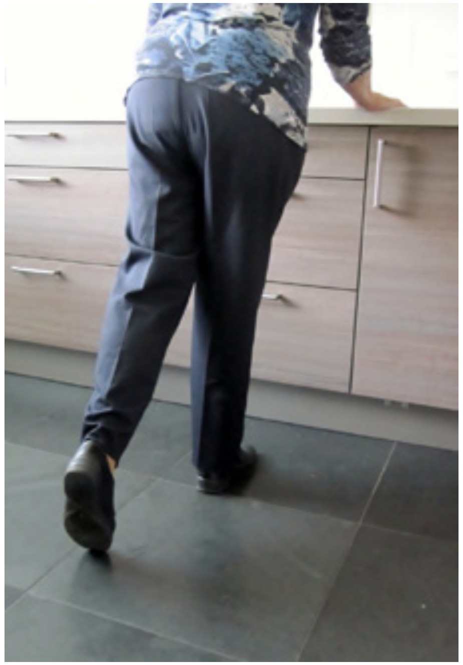

- Beweeg uw linker- en rechterbeen afwisselend naar achteren
- Raak met uw tenen de vloer
- Herhaal 5x

Tip:

U kunt deze oefening moeilijker maken door het been te heffen zonder de vloer te raken met uw tenen

A

B

B

36. Kniebuigingen met steun

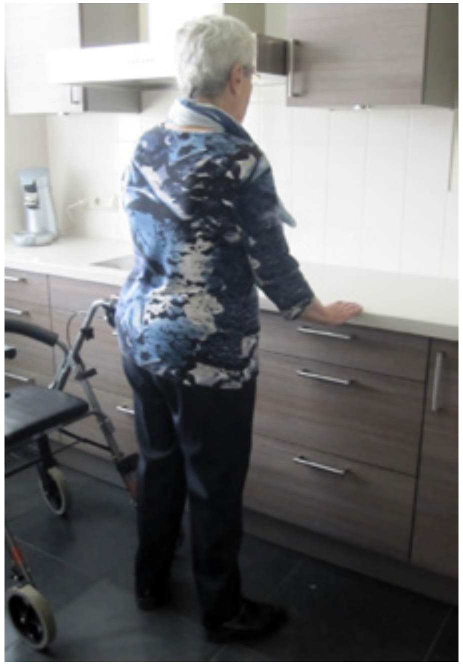

• Rechtop staan bij aanrecht

• Neem licht steun met handen op aanrecht

• Voeten op heupbreedte

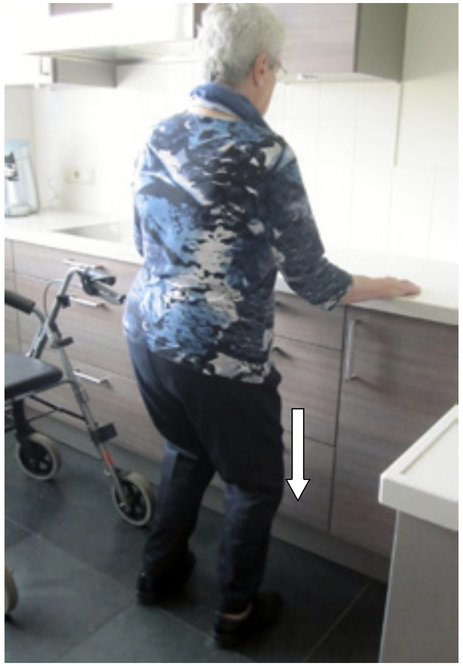

• Buig langzaam een stukje door uw knieën

• En ga weer staan

• Herhaal 5x

37. Tenenstand

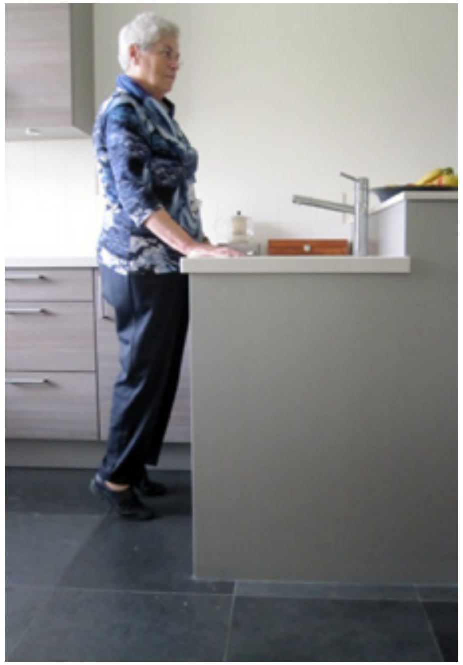

• Handen steunen op aanrecht

• Ga op uw tenen staan

Herhaal 5x

38. Hakkenstand

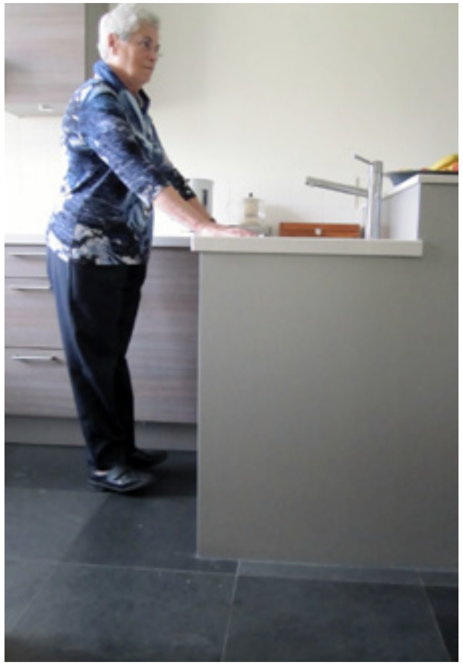

• Handen steunen op aanrecht

• Ga op uw hakken staan

• Herhaal 5x

39. Zijlig been heffen

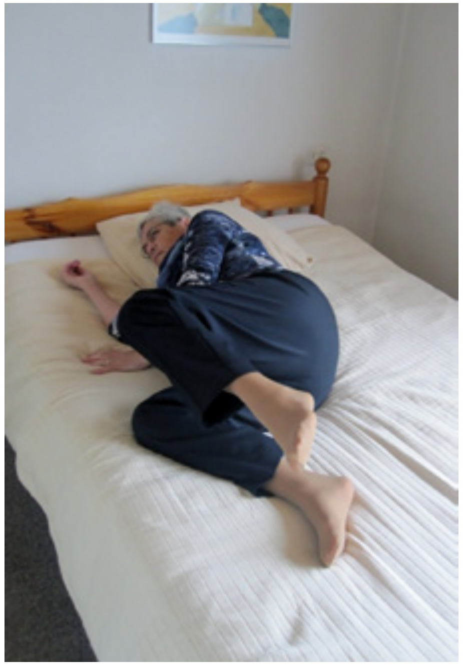

- Zijlig benen gebogen
- Uw bovenste been een stukje optillen
- En omlaag brengen
- Herhaal 1x en wissel van zij

Tip:

Bouw deze oefening voor beide kanten op naar 5x

40. Zijlig been heffen en uitstrekken

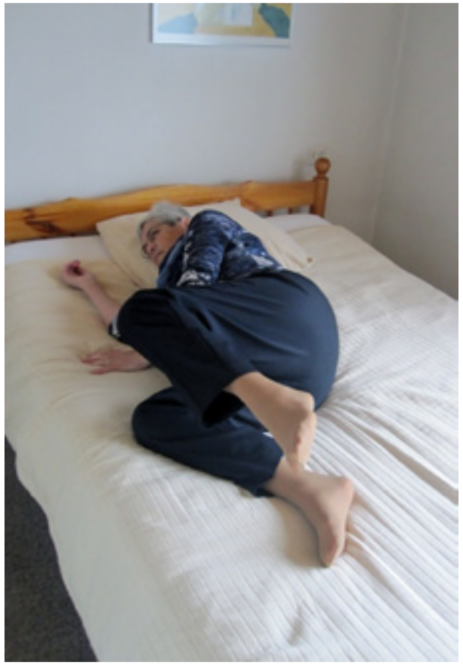

- Zijlig benen gebogen
- Uw bovenste been een stukje optillen

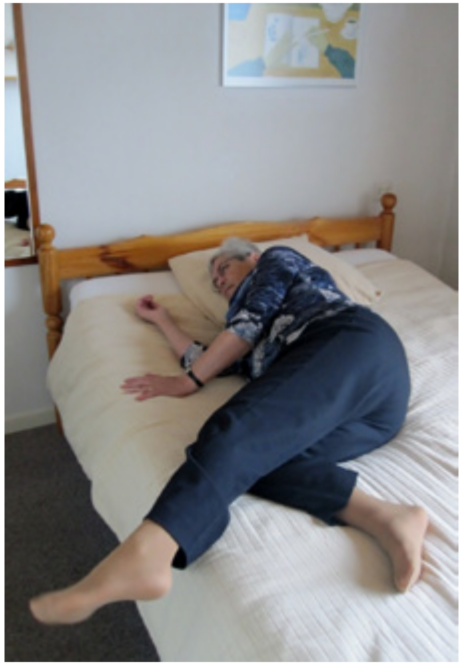

- Strek uw knie en probeer het been omhoog te houden
- Herhaal 1x en wissel van zij

Tip:

Bouw deze oefening voor beide kanten op naar 5x

B

41. Conditie: Buitenshuis lopen

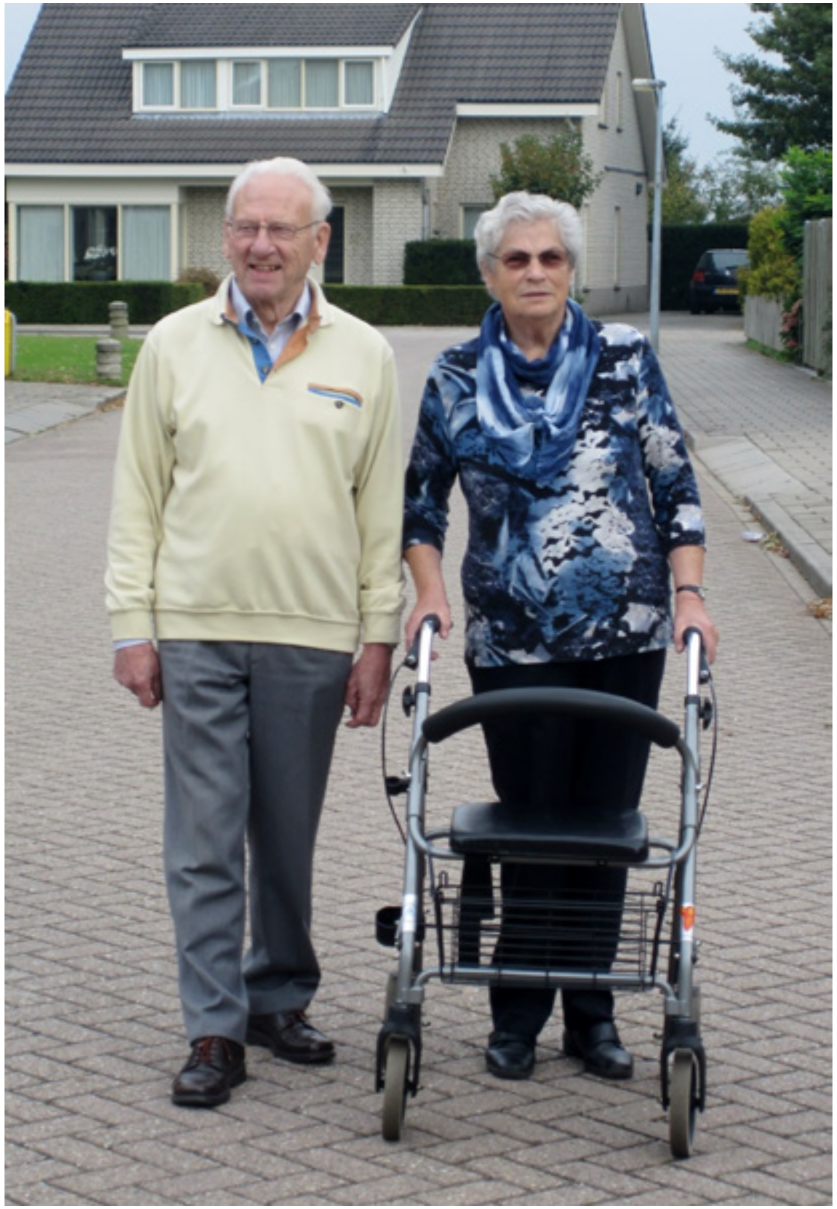

B

- Probeer iedere dag samen met iemand een stukje buiten te lopen
- Stel een haalbaar doel, bijvoorbeeld naar het eind van de straat en terug
- Maak eventueel gebruik van een pauzemoment door te gaan zitten op uw rollator
- Loop in rustig tempo maar probeer ook af en toe een stukje sneller te wandelen
- Probeer de afstand uit te breiden en te streven naar 10 minuten aan één stuk lopen
- Bepaal samen met uw partner of familielid wanneer u zeker genoeg bent om alleen buiten te lopen

B

Instructies Profiel C en D

Hoe kunt u het oefenprogramma gaan gebruiken?

In het oefenboek zijn de oefeningen voor profiel C en D gebundeld. In de kantlijn van het boek kunt u de kleuren zien en zo bepalen of de oefening bij uw profiel hoort. De oranje oefeningen horen bij profiel C en de rode bij profiel D. Sommige oefeningen behoren zowel bij profiel C als D, dan ziet u zowel een oranje als rood vlak in de kantlijn.

Profiel C

(oranje oefeningen)

In profiel C zijn de oefeningen zodanig opgebouwd dat u met meer vertrouwen zelfstandig binnen- en buitenshuis kunt functioneren waarbij uw loopkwaliteit, loopsnelheid, balans, kracht en conditie verbeteren.

Profiel D

(rode oefeningen)

In profiel D gaat u net als in profiel C oefenen met het doel om met meer vertrouwen binnen- en buitenshuis te kunnen functioneren. Het oefenprogramma is gericht op het verbeteren van uw loopkwaliteit, loopsnelheid, en conditie maar bevat tevens complexere balans– en krachtoefeningen.

Nederlandse Norm Gezond bewegen

Voldoende beweging zorgt ervoor dat u zich fitter gaat voelen. U beweegt voldoende wanneer u op minimaal vijf, maar bij voorkeur alle dagen van de week, 30 minuten matig intensief beweegt. Dit is de Nederlandse Norm voor Gezond Bewegen. Een matig intensieve beweging is een beweging waarvan u sneller gaat ademhalen. U krijgt het er warm van en uw hart gaat er wat sneller van kloppen.

Als u door beperkingen niet meer aan 30 minuten matig intensief bewegen toekomt, is elke extra hoeveelheid lichaamsbeweging zinvol, ongeacht duur, intensiteit, frequentie of type.

Oefenprogramma

Advies: 3x per week 30 minuten oefenen aan de hand van de door u uitgekozen oefeningen uit het oefenprogramma. U kunt de oefeningen ook opsplitsen in bijvoorbeeld 2x 15 minuten.

Begin het programma altijd even met rustige warming up oefeningen uit het onderdeel ‘Oefeningen ter verbetering van uw algehele fitheid’. Zie p. 48 t/m 51 voor het samenstellen van een oefenprogramma. Gebruik hierbij de inhoudsopgave op p. 52 t/m 53.

Wandelprogramma

Advies: ga minimaal 2x per week 30 minuten wandelen. U kunt indien nodig hiervoor een stok, rollator of nordic walking stokken gebruiken.

Het kan zijn dat 30 minuten wandelen nog niet mogelijk voor u is. Stel dan voor uzelf een persoonlijk doel op wat wel haalbaar is, bijvoorbeeld lopen naar de winkel om de hoek. Ook meerdere keren per dag een stukje wandelen is een optie. Probeer de afstand en tijd uit te breiden en te streven naar 30 minuten aan één stuk lopen. Maak eventueel gebruik van een rustmoment op een bankje.

Veiligheid

Voelt u zich niet lekker, heeft u acute pijnklachten, last van duizeligheid of bent u ziek, doe het dan even wat rustiger aan. Raadpleeg eventueel uw huisarts.

Mocht u tijdelijk, door bijvoorbeeld ziekte, niet hebben kunnen oefenen bepaal dan opnieuw welke oefeningen geschikt voor u zijn.

Tip:

Kijk uit met buitenshuis oefenen of wandelen tijdens gladheid in de winter. U zou op dat moment ook kunnen kiezen om hiervoor in de plaats oefeningen binnenshuis te doen.

Profiel C. Hoe kunt u een oefenprogramma samenstellen?

(Gebruik een kopie van het invulformulier op de pagina hiernaast)

Stap 1. Maak een prioriteitenlijst van (voor u) moeilijke activiteiten

Bijvoorbeeld:

1. Balans
2. Gaan liggen in bed
3. Traplopen
4. Buiten lopen
5. Opstaan uit bed

Stap 2. Selecteer de bijbehorende oranje oefeningen

Gebruik de inhoudsopgave op p. 52 t/m 53 om de oefeningen die bij de moeilijke activiteit horen te selecteren. Advies: voeg altijd looptraining toe.

Voorbeeld moeilijke activiteit: balans.

- Warming up: oefening 25, 26, 27, 28, 29, 30
- Moeilijke activiteit (balans): oefening 8, 9, 10, 11, 12, 31, 32, 33, 39, 40, 41, 42, 43
- Looptraining: oefening 15, 16, 17, 18, 19, 20, 21

Stap 3. Stel uw oefenprogramma samen

Start met een deel van de oefeningen. Sluit aan bij wat u kunt en/of wat u leuk vindt.

Bijvoorbeeld:

- Warming up: oefening 25, 26, 27, 33
- Balans: oefening 8, 9, 10,11
- Looptraining: oefening 15, 16, 17

Stap 4. Uitbreiden van oefenprogramma

Als u de oefening goed beheerst kunt u doorgaan met een volgende oefening uit stap 2. Of gebruik de andere oefeningen ter afwisseling.

Stap 5. Maak een nieuw oefenprogramma

Neem nu de volgende moeilijke activiteit van uw prioriteitenlijst en herhaal stap 2, 3 en 4 op een nieuw formulier. Herhaal ook regelmatig oefeningen uit het voorgaande oefenprogramma.

Oefenprogramma C

Schrijf niet
op deze pagina!

Stap 1. Maak een prioriteitenlijst van moeilijke activiteiten

1. ....
2. ....
3. ....
4. ....
5. ....

Stap 2. Selecteer de bijbehorende oranje oefeningen

Moeilijke activiteit: ....

- Warming up: oefening ....
- Moeilijke activiteit: oefening ....
- Looptraining: oefening ....

Stap 3. Stel uw oefenprogramma samen

- Warming up: oefening ....
- Moeilijke activiteit:oefening ....
- Looptraining: oefening ....

Stap 4. Uitbreiden van oefenprogramma

- Warming up: oefening ....
- Moeilijke activiteit:oefening ....
- Looptraining: oefening ....

Stap 5. Maak een nieuw oefenprogramma. Selecteer de bijbehorende oefeningen.

Neem nu de volgende moeilijke activiteit van uw prioriteitenlijst en herhaal stap 2, 3 en 4 op een nieuw formulier. Herhaal ook regelmatig oefeningen uit het voorgaande oefenprogramma.

Profiel D. Hoe kunt u een oefenprogramma samenstellen?

(Gebruik een kopie van het invulformulier op de pagina hiernaast)

Stap 1. Maak een prioriteitenlijst van (voor u) moeilijke activiteiten

Bijvoorbeeld:

1. Opstaan van de grond
2. Balans
3. Kracht been spieren
4. Buiten lopen
5. Opstaan uit bed

Stap 2. Selecteer de bijbehorende rode oefeningen

Gebruik de inhoudsopgave op p. 52 t/m 53 om de oefeningen die bij de moeilijke activiteit horen te selecteren. Advies: voeg altijd looptraining toe.

Voorbeeld moeilijke activiteit: opstaan van de grond.

- Warming up: oefening 25, 26, 27, 28, 29, 30, 31, 32, 33
- Moeilijke activiteit (opstaan van de grond): oefening 6a, 6b, 6c, 7

Stap 3. Stel uw oefenprogramma samen

Start met een deel van de oefeningen. Sluit aan bij wat u kunt en/of wat u leuk vindt.

Bijvoorbeeld:

- Warming up: oefening 25, 26, 27, 33
- Opstaan van de grond: 6a
- Looptraining: oefening 15, 16, 17

Stap 4. Uitbreiden van oefenprogramma

Als u de oefening goed beheerst kunt u doorgaan met een volgende oefening uit stap 2. Of gebruik de andere oefeningen ter afwisseling.

Stap 5. Maak een nieuw oefenprogramma

Neem nu de volgende moeilijke activiteit van uw prioriteitenlijst en herhaal stap 2, 3 en 4 op een nieuw formulier. Herhaal ook regelmatig oefeningen uit het voorgaande oefenprogramma.

Oefenprogramma D

Schrijf niet
op deze pagina!

Stap 1. Maak een prioriteitenlijst van moeilijke activiteiten

1. ....
2. ....
3. ....
4. ....
5. ....

Stap 2. Selecteer de bijbehorende rodeoefeningen

Moeilijke activiteit: ....

- Warming up: oefening ....
- Moeilijke activiteit: oefening ....
- Looptraining: oefening ....

Stap 3. Stel uw oefenprogramma samen

- Warming up: oefening ....
- Moeilijke activiteit:oefening ....
- Looptraining: oefening ....

Stap 4. Uitbreiden van oefenprogramma

- Warming up: oefening ....
- Moeilijke activiteit:oefening ....
- Looptraining: oefening ....

Stap 5. Maak een nieuw oefenprogramma. Selecteer de bijbehorende oefeningen.

Neem nu de volgende moeilijke activiteit van uw prioriteitenlijst en herhaal stap 2, 3 en 4 op een nieuw formulier. Herhaal ook regelmatig oefeningen uit het voorgaande oefenprogramma.

Inhoudsopgave Profiel C en D

Oefeningen ter bevordering van het verplaatsen

1. Gaan liggen in bed .... p. 54
2. Opstaan uit bed .... p. 55
3. Bruggetje en zijwaarts verplaatsen in bed .... p. 56
4. Opstaan van stoel .... p. 57
5. Gaan zitten op stoel .... p. 57
6. Veilig naar de grond .... p. 58
6a. Naar schuttershouding .... p. 58
6b. Naar knieënstand .... p. 59
6c. Naar zijwaartse zit en lig .... p. 59
7. Veilig opstaan van de grond via zijlig .... p. 60

Oefeningen ter bevordering van het reiken

8. Zijwaarts met één hand reiken .... p. 61
9. Zijwaarts met twee handen reiken .... p. 61
10. Reiken in de hoogte .... p. 61
11. Reiken in de laagte .... p. 62
12. Reiken achterwaarts .... p. 62
13. Kratje verplaatsen tafel-stoel .... p. 63
14. Kratje verplaatsen van tafel naar vloer .... p. 63

Looptraining

Oefeningen en tips voor bevordering van de loopkwaliteit en de loopsnelheid in huis en buitenshuis en veilig traplopen

15. Voorwaarts/achterwaarts lopen .... p. 64
16. Zijwaarts lopen .... p. 64
17. Zijwaarts lopen benen kruisen .... p. 65
18. 8-figuur lopen .... p. 65
19. Grotere stappen maken d.m.v. stappen tellen .... p. 66
20. Buitenshuis lopen: verhogen van de loopsnelheid .... p. 67
21. Traplopen .... p. 68
22. Over een voorwerp stappen .... p. 69
23. Over een voorwerp stappen met beker water p. 69
24. Over voorwerp heen stappen met dienblad .... p. 69

Oefeningen ter verbetering van uw algehele fitheid

Warming up en ondersteuning van reiken en grijpen

25. Armen voorwaarts heffen .... p. 70

C

D

26. Armen zijwaarts heffen .... p. 70
27. Hoofd draaien .... p. 70

Warming up en ondersteuning van lenigheid en kracht

28. Strekken knie .... p. 71
29. Knieheffen .... p. 71
30. Tenen naar u toe trekken en wegduwen .... p. 71
31. Zijwaarts been heffen .... p. 72
32. Achterwaarts heffen .... p. 72
33. Kniebuigingen .... p. 72

Ter ondersteuning van kracht in armen

34. Opdrukken stoelleuning/zitting .... p. 72

Ter ondersteuning van kracht in bovenbeenspieren, opstaan en gaan zitten in stoel

35. Functionele krachtoefening beenspieren .... p. 73

Ter ondersteuning van kracht in bil- en beenspieren, zijwaarts verplaatsen in bed en in en uit bed komen

36. Bruggetje .... p. 73
37. Zijlig been heffen .... p. 74
38. Zijlig been heffen en uitstrekken .... p. 74

Ter ondersteuning van balans, kracht en lopen

39. Tenenstand .... p. 74
40. Hakkenstand .... p. 75
41. Voeten voor elkaar plaatsen .... p. 75
42. Op één been staan .... p. 75
43. Voeten plaatsen op traptrede .... p. 75
44. Voor- en achterwaarts over voorwerp heen stappen .... p. 76
45. Voeten omlaag plaatsen en achterwaarts omhoog .... p. 76
46. Zijwaarts op– en afstappen .... p. 77
47. Bruggetje met één been .... p. 77

Ter ondersteuning voor het opbouwen van conditie

48. Verbeteren van conditie d.m.v. wandelprogramma .... p. 78

C

D

1. Gaan liggen in bed

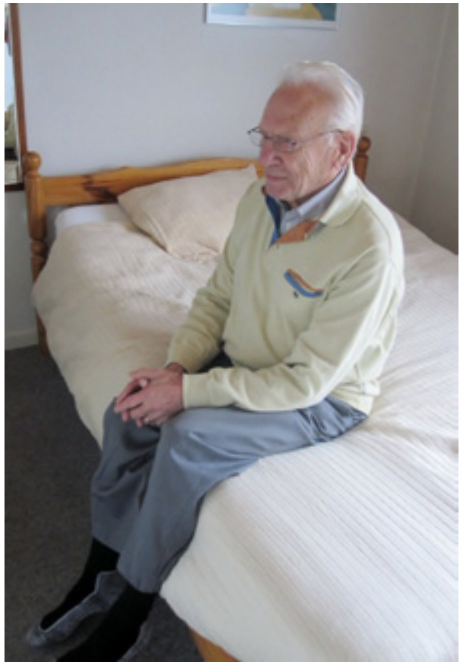

- Zit op bedrand

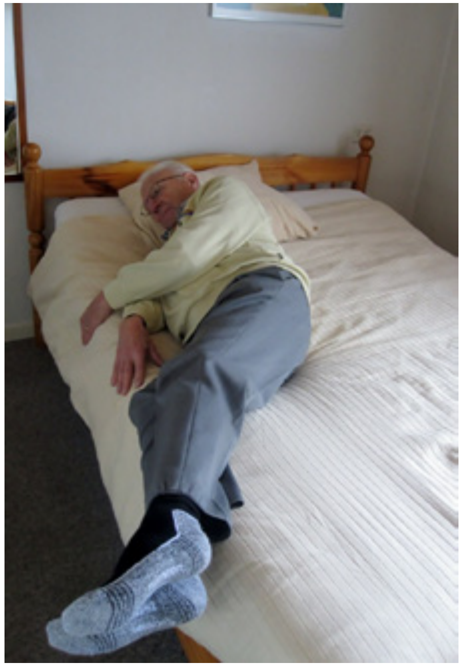

- Ga op de zij liggen
- Neem steun op uw rechterelleboog en linkerhand

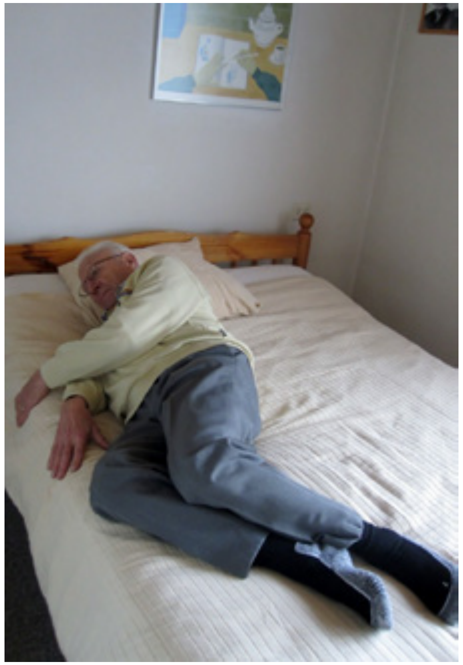

- Til uw voeten in bed

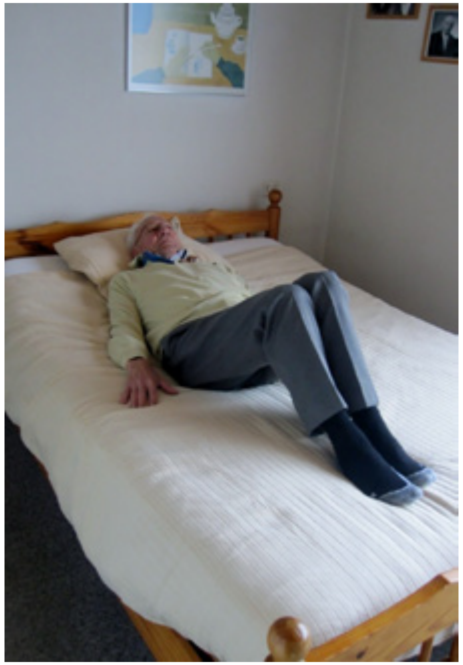

- Draai op de rug

Let op!

Gaat u via de andere kant in bed liggen? Steun op uw linkerelleboog en rechterhand bij het gaan liggen.

2. Opstaan uit bed

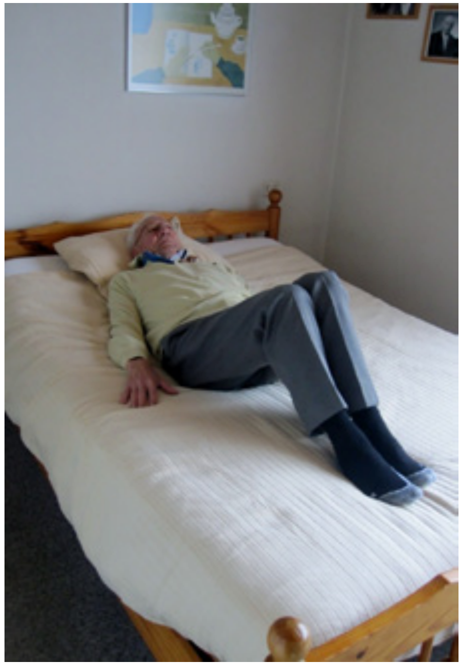

• Plaats uw voeten plat op het bed

• De knieën gebogen

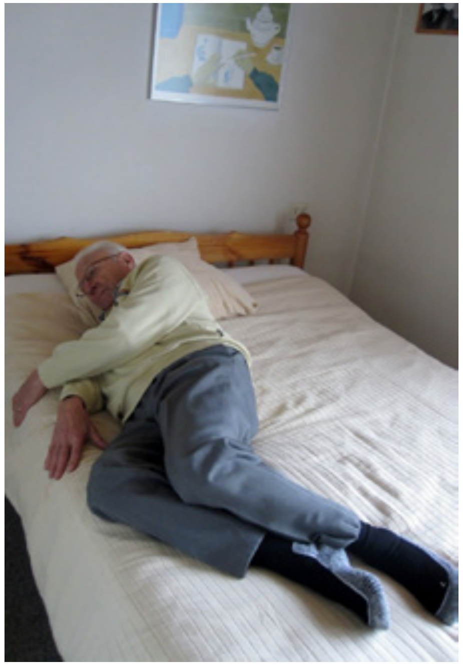

• Breng uw linkerhand naar de bedrand

• En draai op de rechterzij

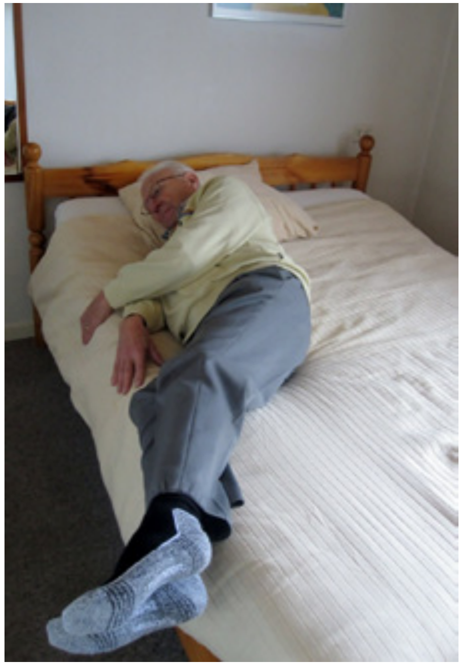

• Neem steun op uw rechterelleboog en linkerhand

• Breng de voeten buiten het bed

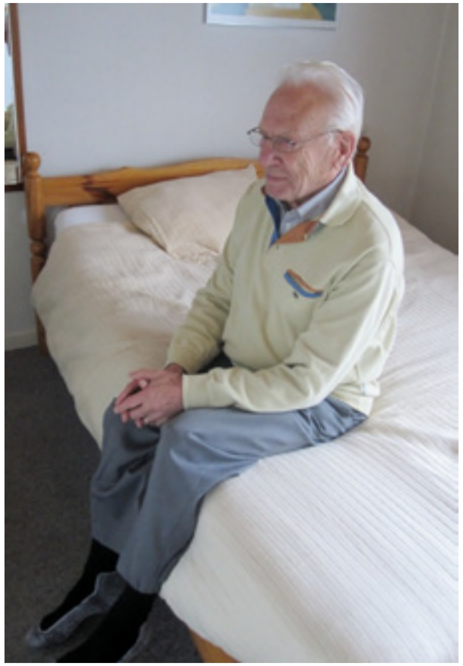

• Kom rustig tot zit

• Blijf even zitten indien u duizelig bent

Let op!

• Komt u via de andere kant uit bed? Draai op de linkerzij door uw rechterhand naar de bedrand te plaatsen. Steun op uw linkerelleboog en rechterhand bij het tot zit komen.

• Controleer uw afstand tot de rand van het bed door uw arm ter hoogte van uw schouder zijwaarts te plaatsen. De elleboog mag niet over de rand van het bed komen. Zo ja, doe eerst oefening 3 naar links om draairuimte te creëren.

3. Bruggetje en zijwaarts verplaatsen in bed

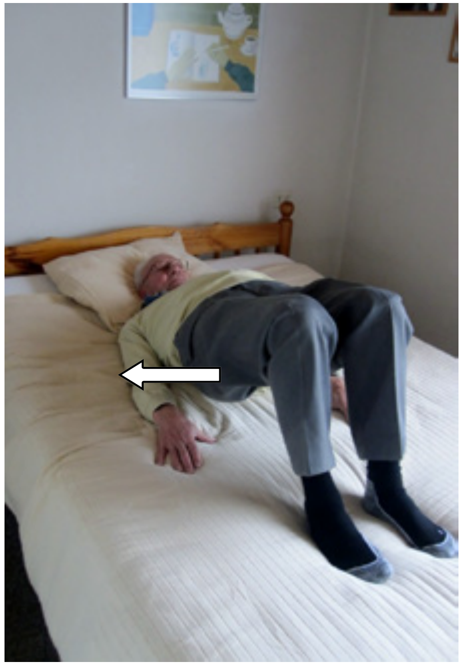

Tip:

Oefen het zijwaarts verplaatsen naar beide kanten. Het zijwaarts verplaatsen is belangrijk om voldoende ruimte te creëren voor het omdraaien.

- Plaats uw voeten plat op het bed
- Breng uw hakken zo ver mogelijk naar uw billen
- Til uw billen op (bruggetje)
- Verplaats de billen naar rechts
- Verplaats uw voeten naar rechts
- Verplaats uw bovenlichaam naar rechts

C

D

4. Opstaan van stoel

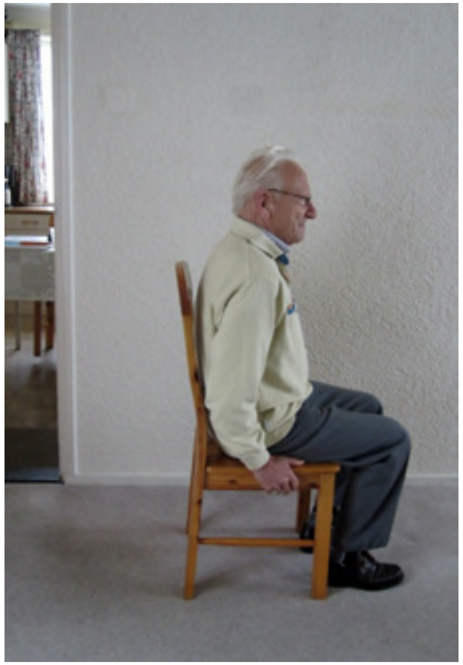

- Zet de voeten stevig op de grond
- Een stukje uit elkaar
- Plaats de handen op de stoelleuning of stoelzitting

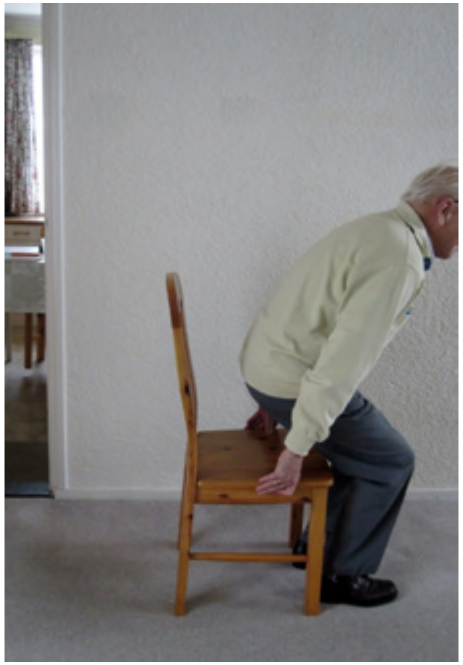

- Kom met het bovenlichaam naar voren en omhoog (sta op zoals een vliegtuig opstijgt en niet zoals een raket)

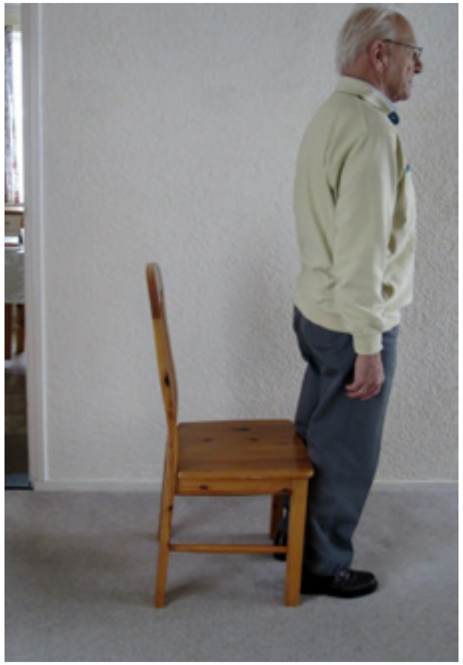

- Ga rustig staan
- Eerst de balans vinden en dan pas verder gaan

Tip profiel D:

Probeer tevens op te staan zonder steun aan leuning of zitting

5. Gaan zitten op stoel

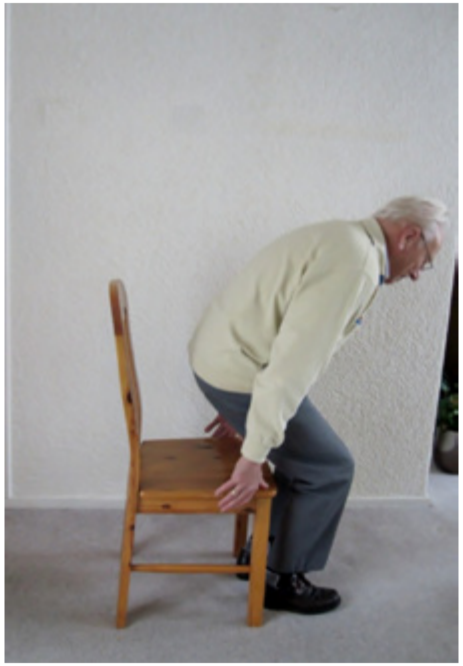

- Pak met beide handen de zitting of stoelleuning vast
- Ga rustig zitten (niet laten vallen op stoel)

Tip profiel D:

Probeer tevens op te staan zonder steun aan leuning of zitting

C

D

6. Veilig naar de grond

6a. Naar schuttershouding

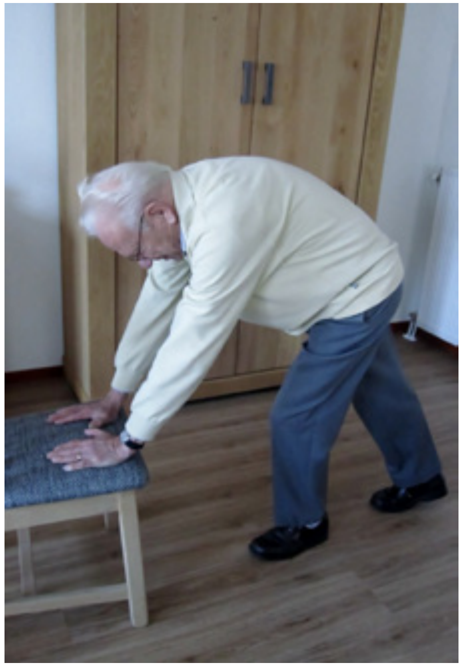

- Neem met de handen steun op een stoel
- Plaats uw voeten één voetlengte achter elkaar met het sterkste been voor

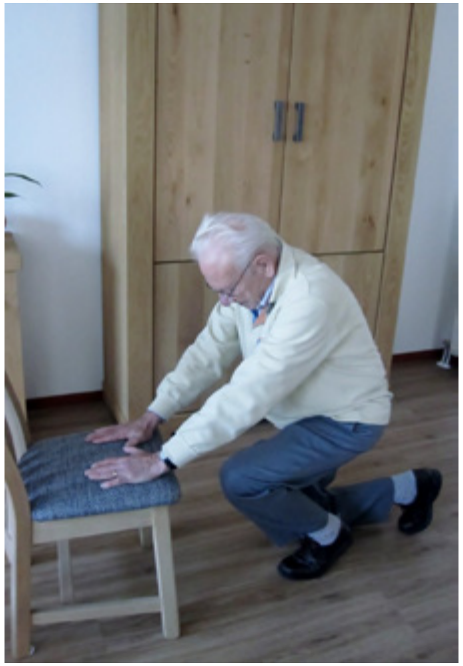

- Zak door uw knie (achterste been) naar de grond
- U bent nu in schuttershouding
- Ga vervolgens weer staan met de handen gesteund op de stoel

Tips:

- U wordt geadviseerd om de oefeningen 6a t/m 7 uit te voeren in het bijzijn van iemand die u eventueel kan helpen met opstaan
- Bouw oefening 6a, 6b, 6c en 7 op. Indien u oefening 6a goed kunt uitvoeren gaat u pas verder met oefening 6b en zo verder
- Oefening 6a t/m 7 niet uitvoeren als u knie- of heupproblemen of een knie- of heupprothese heeft. Overleg eerst met uw fysiotherapeut of huisarts of deze oefeningen geschikt voor u zijn

D

6b. Naar knieënstand

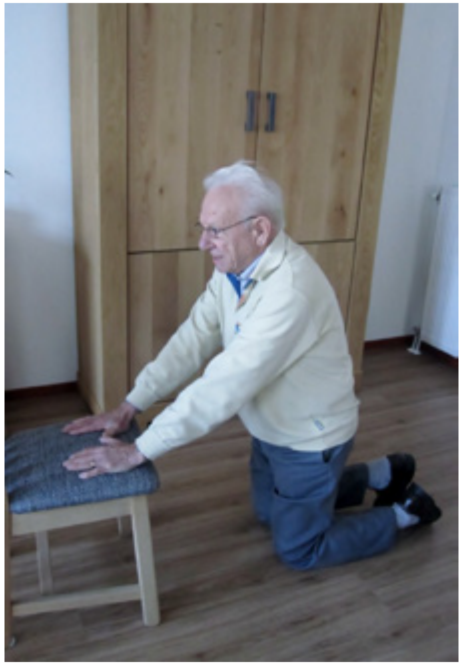

- Kom in schuttershouding (zie voorgaande oefening)
- Ga naar knieënstand
- Kom vervolgens via schuttershouding tot stand (zie voorgaande oefening)

6c. Naar zijwaartse zit en lig

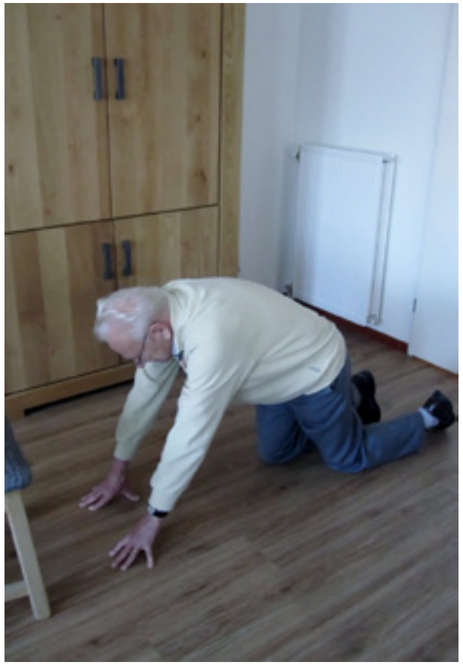

- Ga via knieënstand (zie vorige oefening) naar kruiphouding

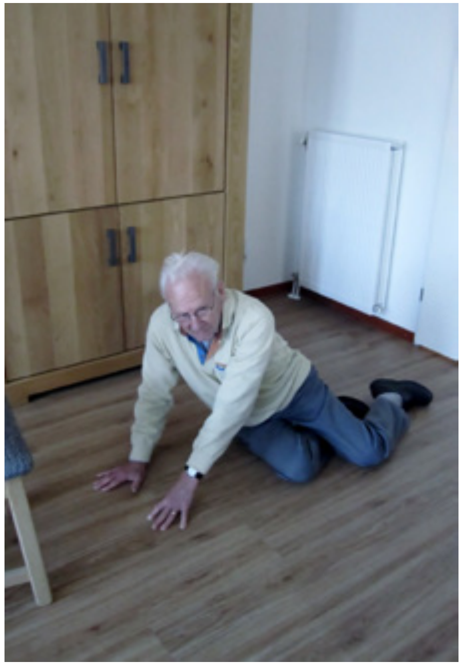

- Kom vanuit kruiphouding naar zijwaartse zit door met uw rechterbil naast uw rechter onderbeen te gaan zitten

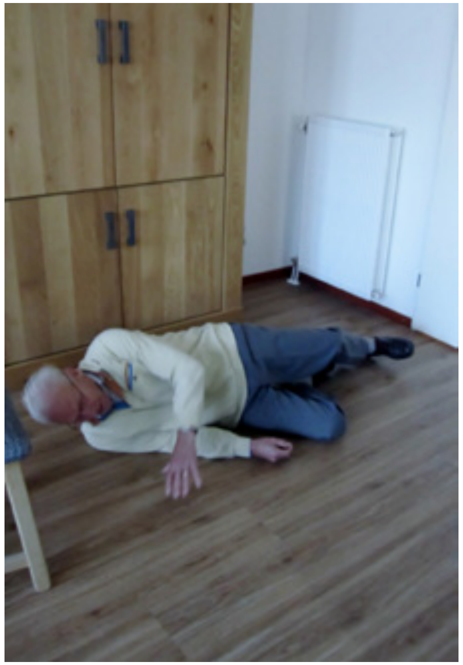

- Steun op uw handen en ga rustig op uw zij liggen
- Sta via oefening 7 veilig op vanaf de grond

Tip:

Probeer uit of het eventueel prettiger is tot zijwaartse zit te komen door met uw linkerbil naast uw linkerbeen te gaan zitten

D

7. Veilig opstaan van de grond via zijlig

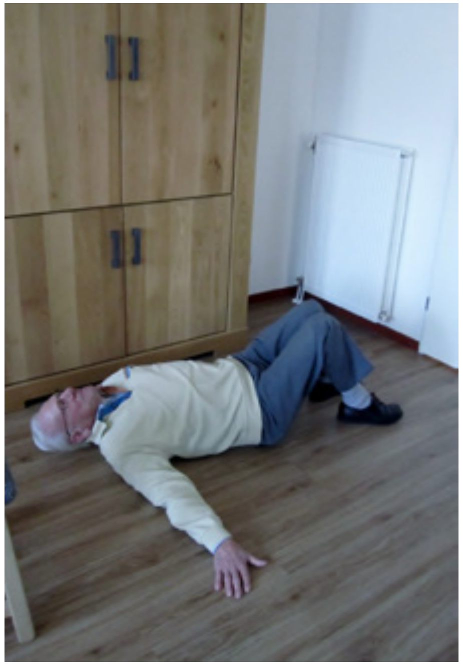

- Lig op grond
- Buig beide knieën
- Plaats de arm zijwaarts met de handpalm omlaag

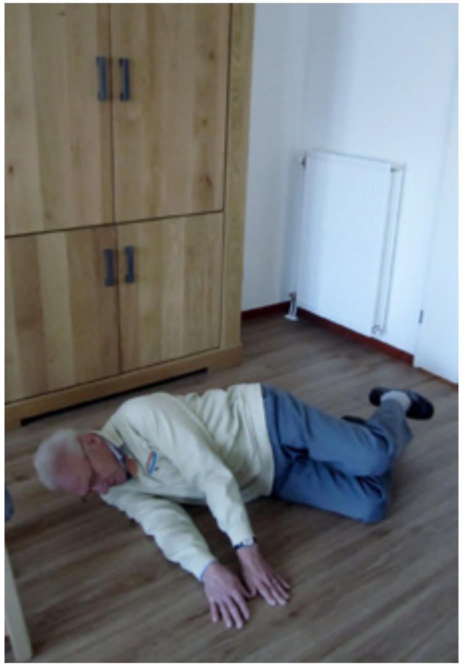

- Draai op uw zij

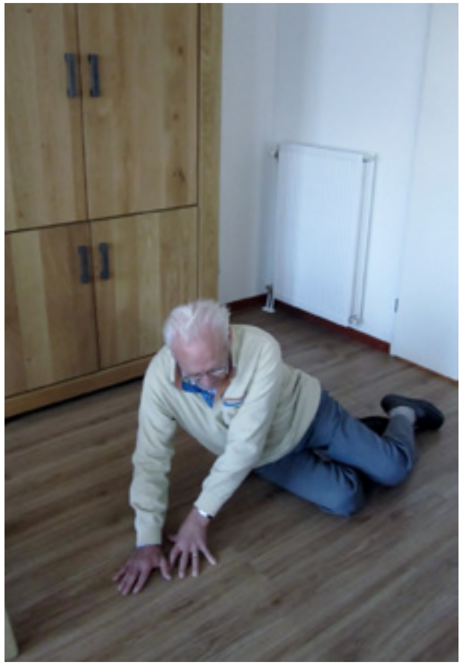

- Steun via uw rechterelleboog op beide handen en duw u naar zijwaartse zit
- Draai over uw gebogen benen en duw met steun op uw handen naar kruiphouding

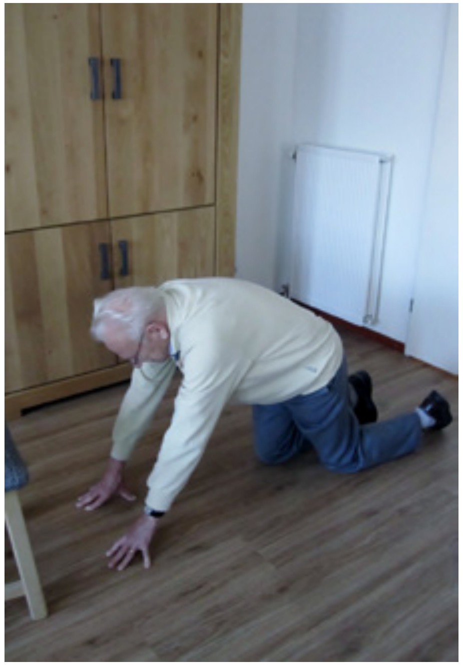

- Kruip naar de stoel
- Neem steun op stoel en kom via knieënstand en schuttershouding tot stand

Tip:

Probeer uit of het eventueel prettiger is om via uw andere zij tot zijwaartse zit te komen

D

8. Zijwaarts met één hand reiken

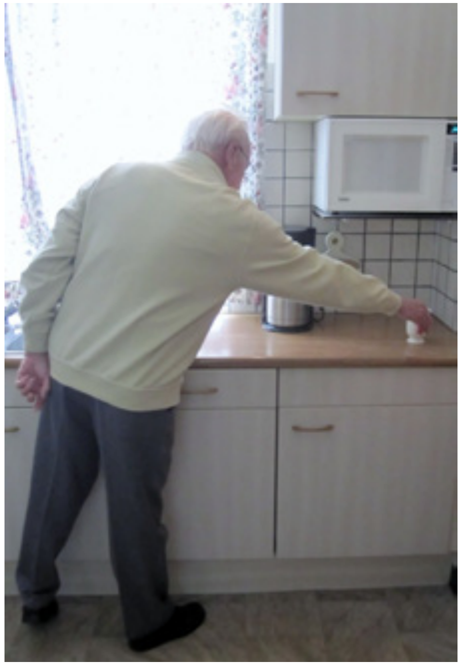

Tip voor profiel D:

Probeer ook net buiten uw steunvlak te reiken door een zijwaartse pas te maken

- Rechtop staan bij aanrecht
- Pak met de rechterhand het kopje door zijwaarts te reiken
- Probeer niet te steunen en reik zo ver mogelijk
- Herhaal 5x voor beide kanten

9. Zijwaarts met twee handen reiken

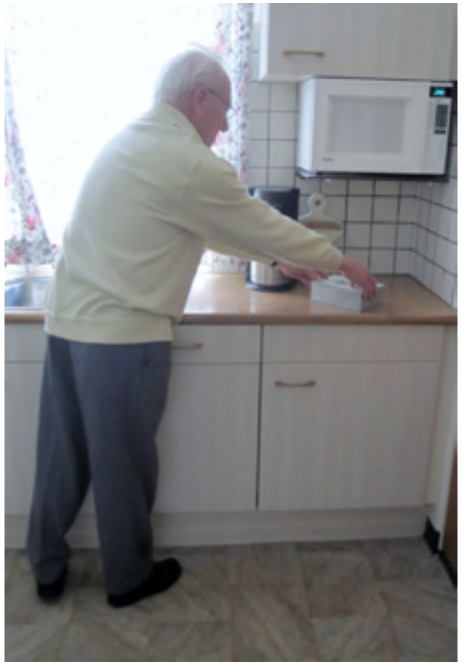

- Rechtop staan bij aanrecht
- Pak met twee handen een voorwerp rechts door te reiken
- Herhaal dit 5x voor beide kanten

Tip:

Probeer zo ver mogelijk te reiken

10. Reiken in de hoogte

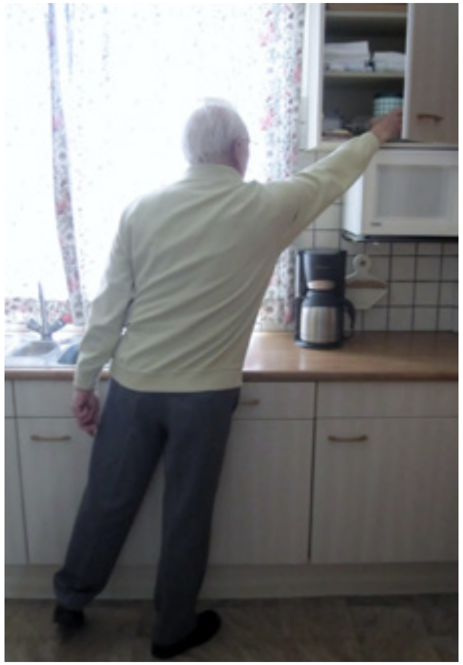

- Reik zijwaarts omhoog en open het aanrechtkastje
- Probeer niet te steunen, pak een voorwerp en plaats het op het aanrecht
- Reik zover mogelijk omhoog en herhaal 5x voor beide kanten

C

D

11. Reiken in de laagte

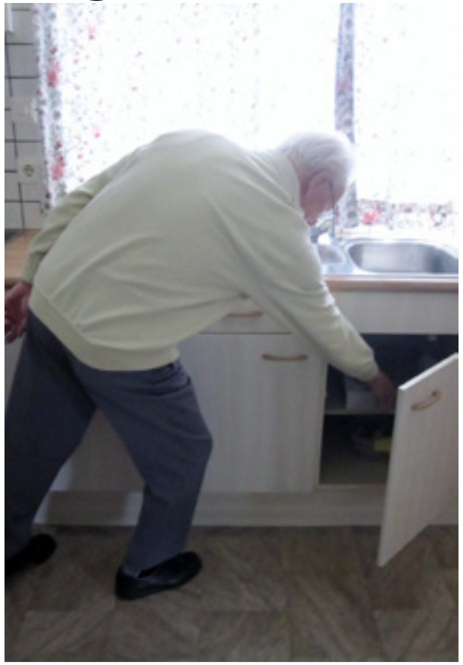

• Reik zijwaarts omlaag en open het aanrechtkastje

• Probeer niet te steunen, pak een voorwerp en plaats het op het aanrecht

• Reik zover mogelijk en herhaal 5x voor beide kanten

Tip voor profiel D:

Probeer ook een voorwerp op te rapen van de grond

12. Reiken achterwaarts

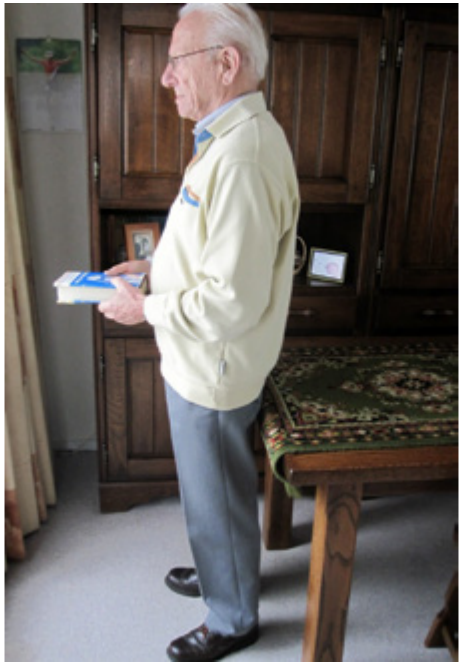

• Ga voor tafel staan, licht gesteund tegen tafel

• Neem een voorwerp in beide handen

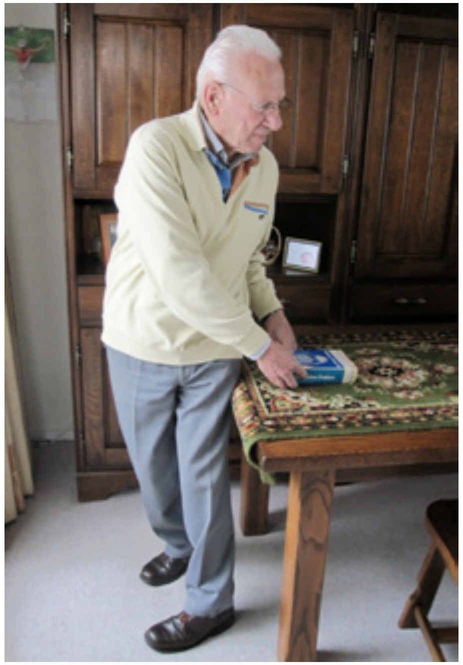

• Draai achterwaarts en leg voorwerp op tafel

• Doe dit voor beide kanten

Tip voor profiel D:

U kunt de oefening moeilijker maken door niet te steunen tegen de tafel

C

D

13. Kratje verplaatsen tafel-stoel

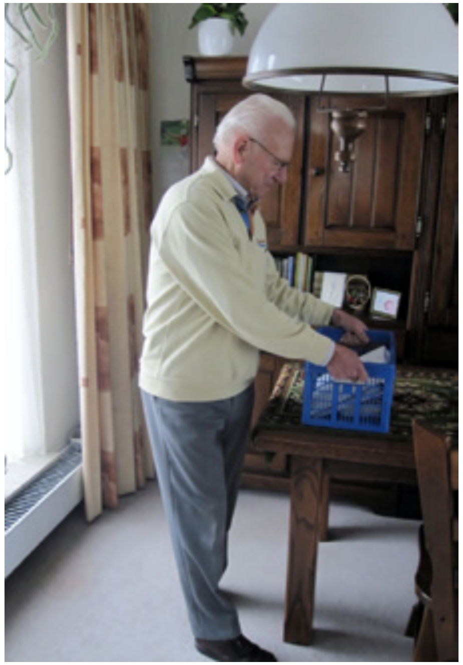

- Verplaats een kratje of doos van tafel naar stoel en weer terug
- Herhaal 5x

14. Kratje verplaatsen van tafel naar vloer

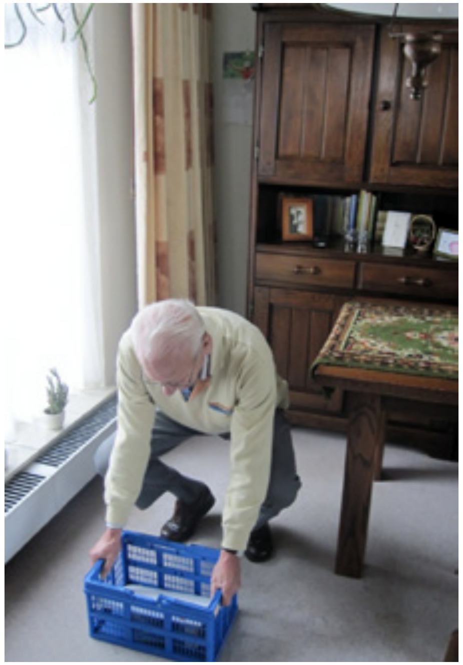

- Zet een kratje of doos op de vloer
- Buig door uw knieën
- Herhaal 1 tot 5x

D

15. Voorwaarts/achterwaarts lopen

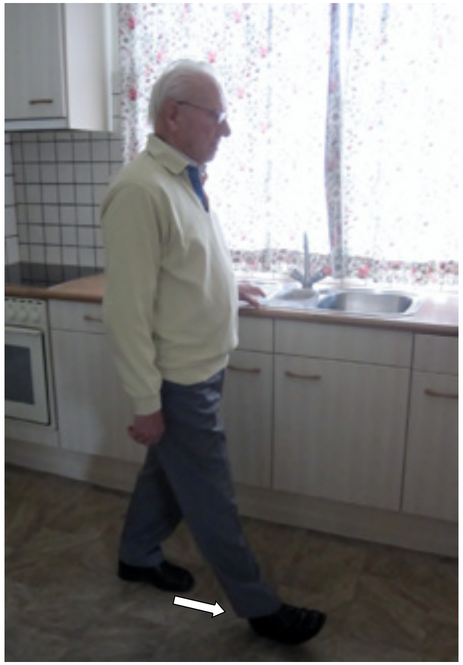

• Loop een aantal passen voorwaarts langs het aanrecht

• Land op de hak en rol uw voet naar voren af

• Probeer zonder steun te lopen

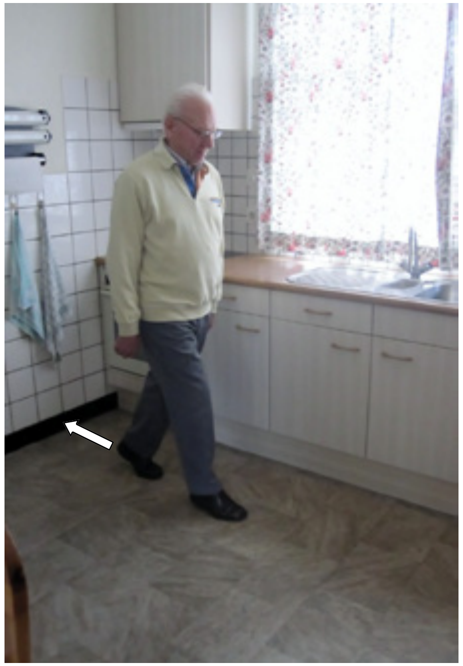

• Loop een aantal passen achterwaarts langs het aanrecht

• Land op de voorvoet en rol uw voet naar achteren af

• Herhaal het voor– en achterwaartse lopen 5x tot 10x

16. Zijwaarts lopen

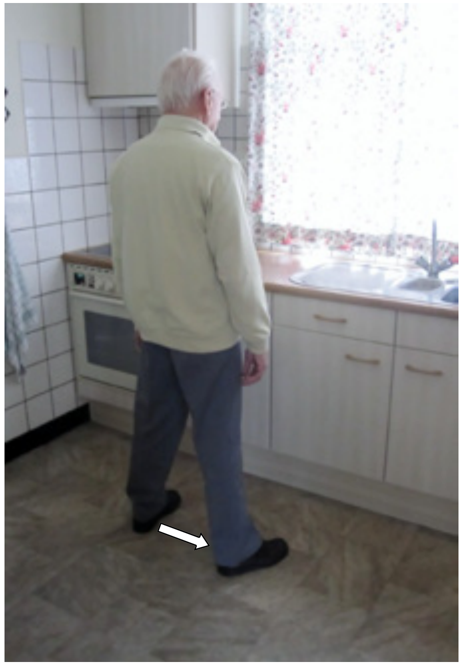

• Loop zijwaarts langs het aanrecht

• Sluit uw voeten aan

• Probeer niet te steunen

• Herhaal 5 tot 10x heen en weer

C

D

17. Zijwaarts lopen benen kruisen

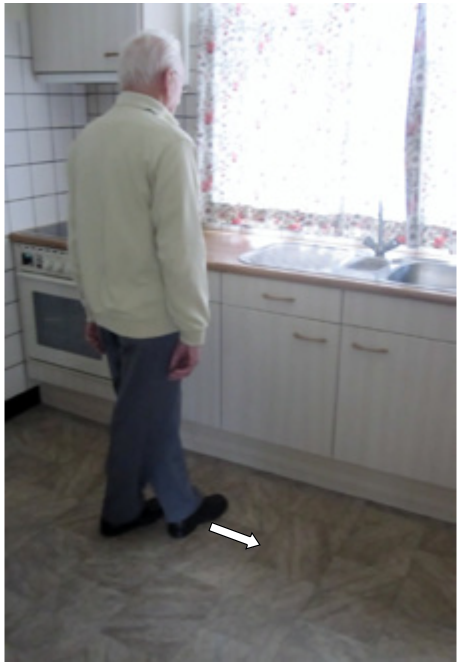

Tip:

U kunt de oefening moeilijker maken door voor– en achterlangs te kruisen

- Loop zijwaarts langs het aanrecht en kruis de benen voorlangs
- Indien nodig, steun nemen

18. 8-figuur lopen

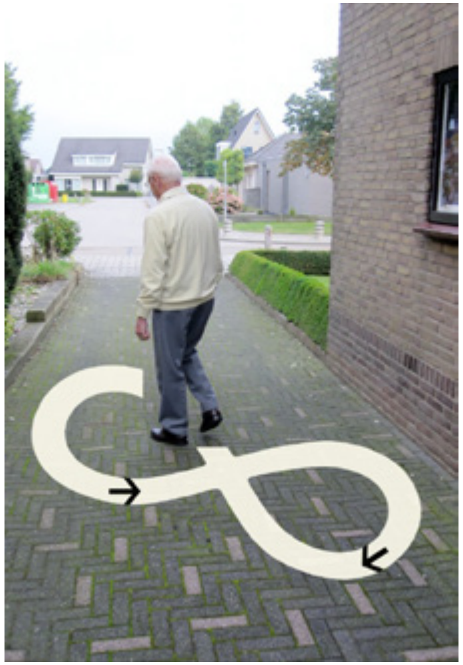

- Loop in een 8-figuur
- Land op de hakken en rol de voeten naar voren af
- Herhaal 5 tot 10x

C

D

19. Grotere stappen maken d.m.v. stappen tellen

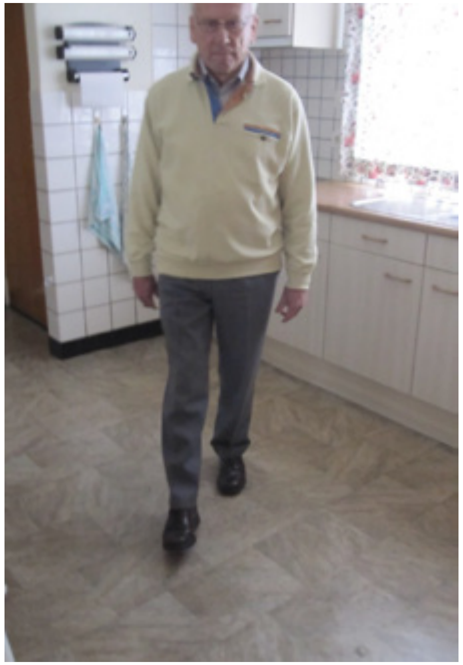

Tips voor profiel D:

- Loop dezelfde route met een beker water en tel het aantal stappen
- Probeer dezelfde route met beker water in minder stappen te lopen
- Herhaal 5 tot 10x

- Loop een bepaalde route in uw huis, bijvoorbeeld van de keuken naar de woonkamer
- Tel het aantal stappen
- Probeer dezelfde route in minder stappen te lopen
- Herhaal 5 tot 10x met grote stappen

C

D

20. Buitenshuis lopen: verhogen van de loopsnelheid

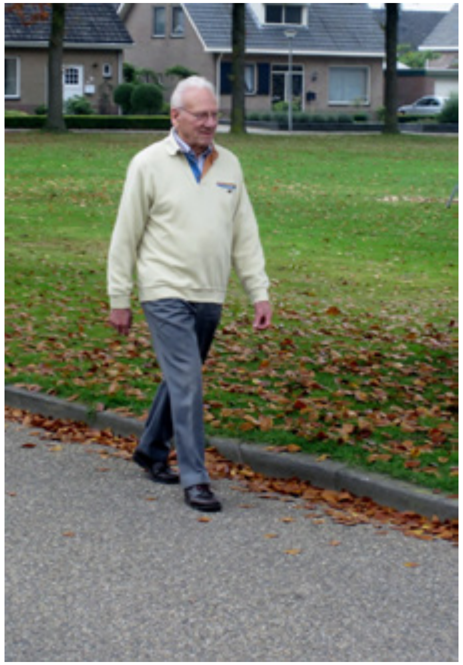

• Loop met aandacht

• Land op de hakken en rol de voeten naar voren af

• Zwaai ontspannen met uw armen mee

• Indien nodig gebruik een stok of rollator

Tips:

• Probeer met aandacht grotere passen te maken

• Probeer ook te variëren in loopsnelheid door stukjes sneller te lopen

• U kunt tevens uw loopsnelheid bevorderen door in de maat te lopen

• Tel bijvoorbeeld 1, 2, 1, 2 etc. of benoem links, rechts, links, rechts etc.

• Ook kan het helpen door bijvoorbeeld in de maat te lopen m.b.v. een metronoom* of op uw muziek via de MP-3 speler

*Een voorbeeld van een geschikte metronoom is de KORG MA-30 te koop of te bestellen bij muziekwinkels

C

D

21. Traplopen

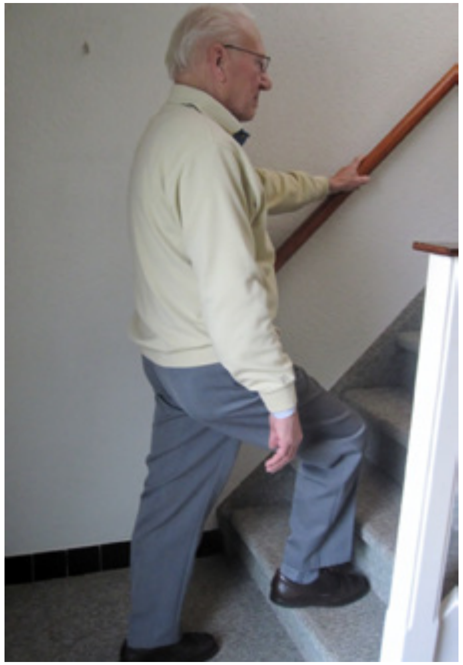

• Pak altijd de trapleuning vast

• Loop in rustig tempo met volle aandacht de trap omhoog en omlaag

Tips:

• Zorg dat er geen voorwerpen op de trap liggen

• Denk aan risico's en uw grenzen wat betreft veiligheid als u bijvoorbeeld met een wasmand naar boven of beneden loopt. U zou bijvoorbeeld ook de was in een tas kunnen doen waardoor het traplopen veiliger wordt.

• Indien u pijnklachten of zwakte in één been heeft, plaats dan bij trap op lopen eerst uw sterkste been omhoog en zet het andere been ernaast

• Bij trap af lopen plaatst u eerst het zwakkere been en zet het sterkere been ernaast

C

D

22. Over een voorwerp stappen

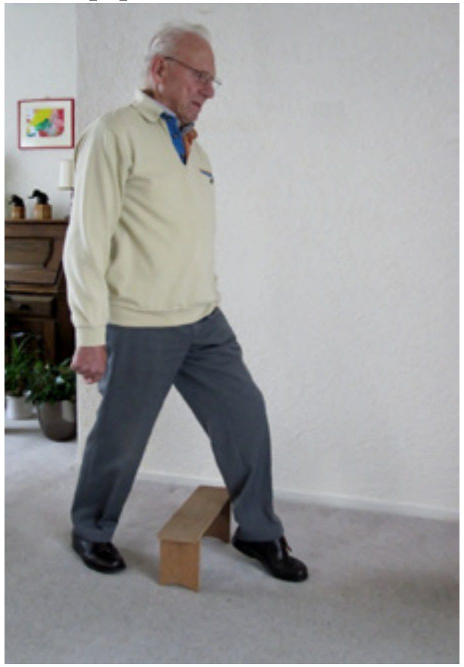

- Loop een route in huis
- Stap voorwaarts over een voorwerp heen
- Herhaal 5 tot 10x

Tip:

Gebruik een soortgelijk voorwerp zoals op de foto met afmetingen van ongeveer 30x15x12cm

23. Over een voorwerp stappen met beker water

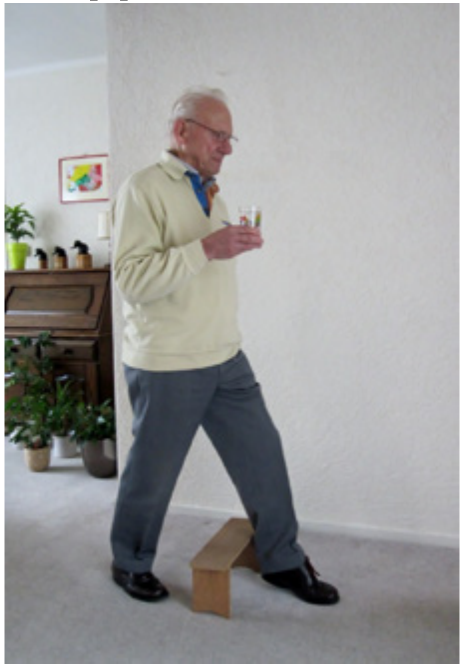

- Loop dezelfde route met een beker water
- Stap over een voorwerp heen
- Herhaal 5 tot 10x

24. Over een voorwerp stappen met dienblad

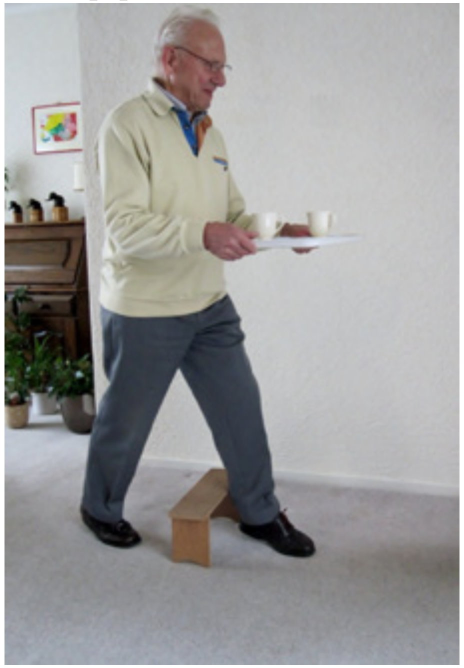

- Loop dezelfde route met een dienblad
- Stap over een voorwerp heen
- Herhaal 5 tot 10x

C

D

25. Armen voorwaarts heffen

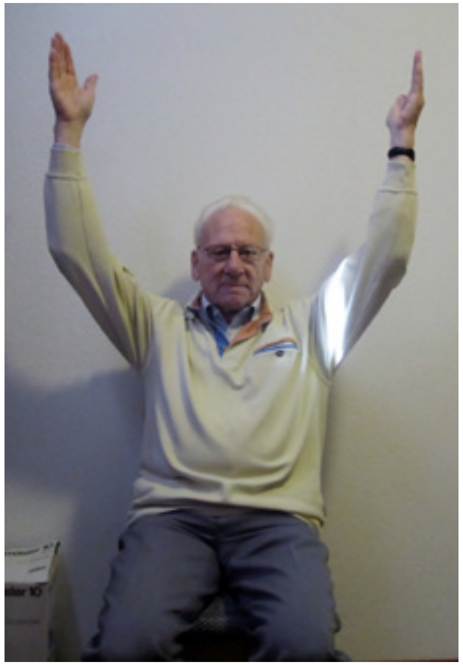

- Breng uw armen in 3 sec. voorwaarts omhoog en adem diep in
- Breng uw armen op de uitademing omlaag in 6 sec.
- Herhaal 10x

26. Armen zijwaarts heffen

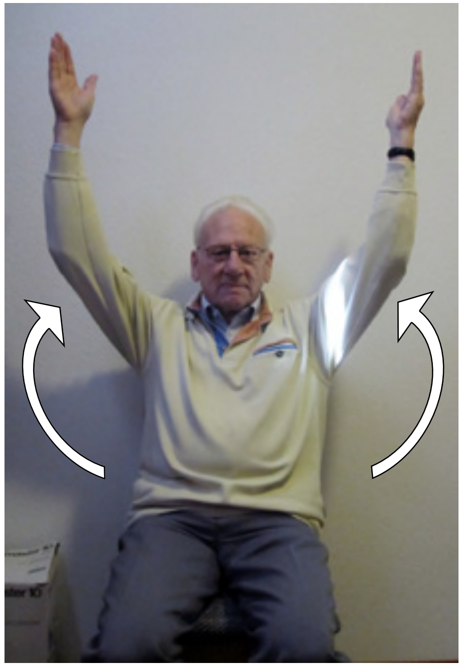

- Breng uw armen in 3 sec. zijwaarts omhoog en adem diep in
- Handpalmen wijzen omhoog naar elkaar toe
- Breng uw armen op de uitademing omlaag in 6 sec.
- Herhaal 10x

Tip:
U kunt oefening 1 en 2 wat zwaarder maken door bijvoorbeeld een pak rijst of flesje water in de handen te nemen

27. Hoofd draaien

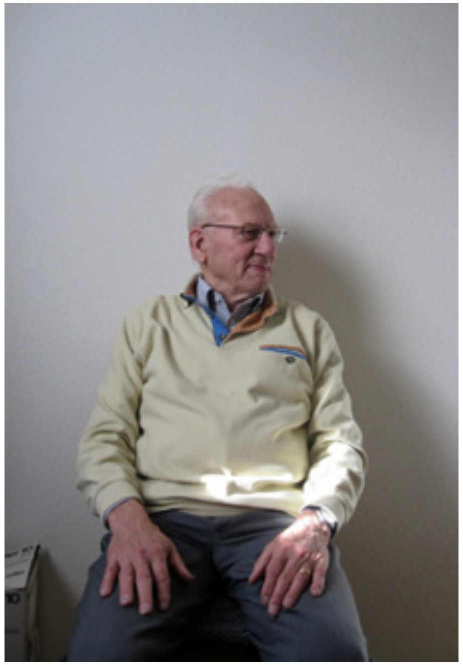

- Draai uw hoofd zo ver mogelijk naar links
- Probeer over uw schouder te kijken
- Doe dit vervolgens ook naar rechts
- Herhaal 10x naar beide kanten

28. Strekken knie

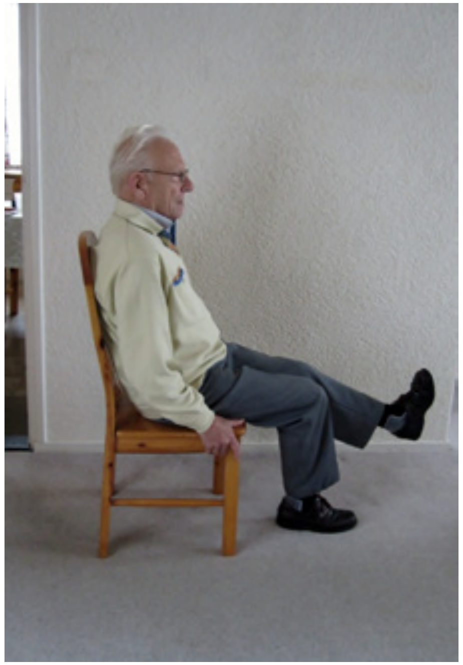

- Strek de linkerknie uit in langzaam tempo (2 sec.)
- Het bovenbeen rust op de stoel
- Buig het linkerbeen in 4 sec. naar de beginpositie
- Herhaal 10x voor beide benen

29. Knieheffen

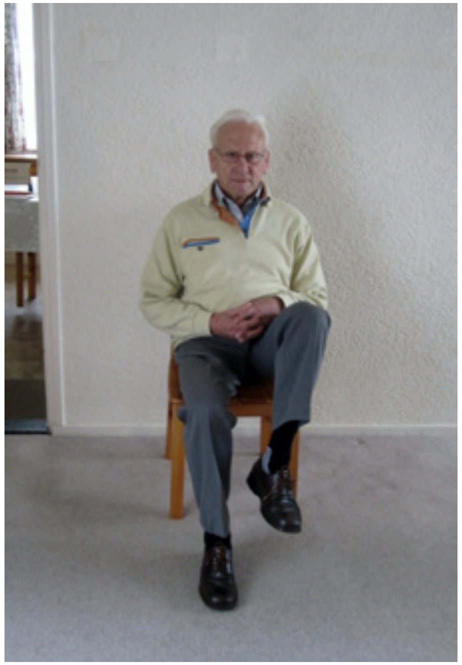

- Til de linkerknie op in 2 sec.
- Breng hem in 4 sec. terug naar de beginpositie
- Herhaal 10x voor beide benen

30. Tenen naar u toe trekken en wegduwen

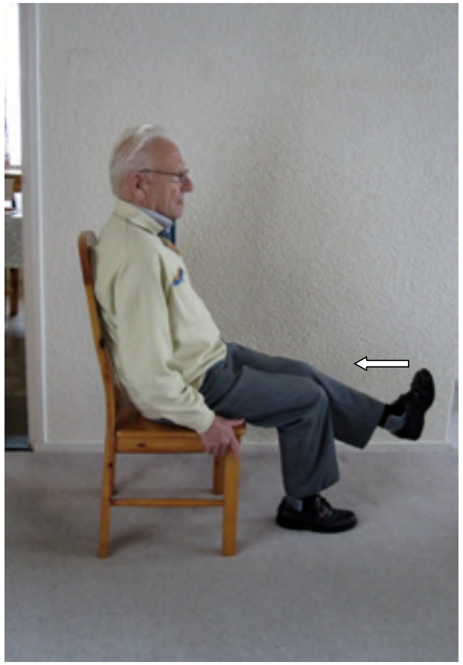

- Strek uw been uit
- Tenen naar u toe trekken
- Hou 2 sec. vast

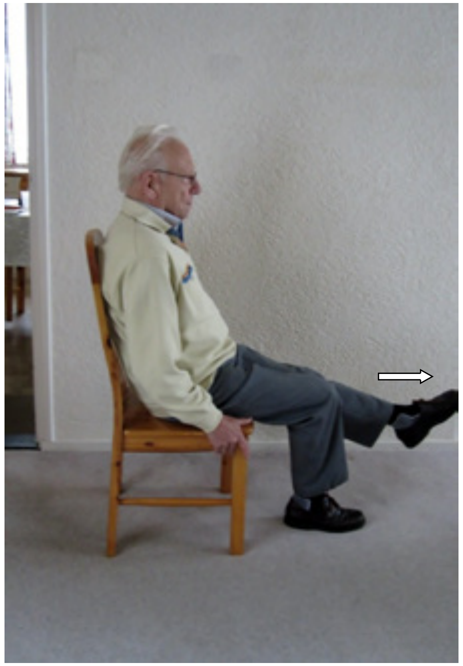

- En afwisselend wegduwen
- Hou 2 sec. vast
- Herhaal 10x keer voor beide voeten

C

D

31. Zijwaarts been heffen

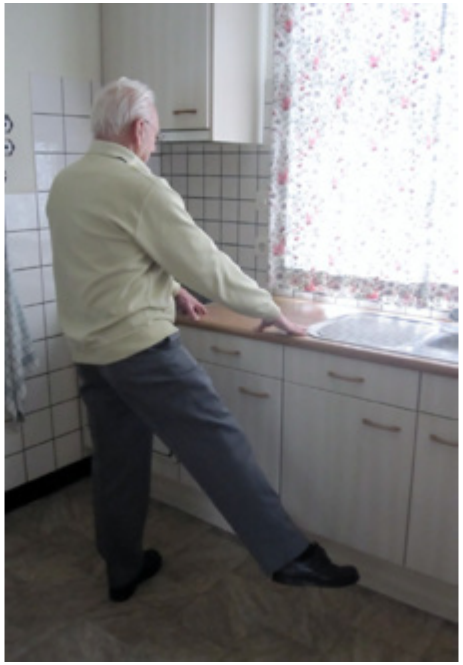

- Rechtop staan
- Indien nodig handen licht steunend
- Beweeg het rechterbeen zijwaarts
- Herhaal ieder been 10x

32. Achterwaarts heffen

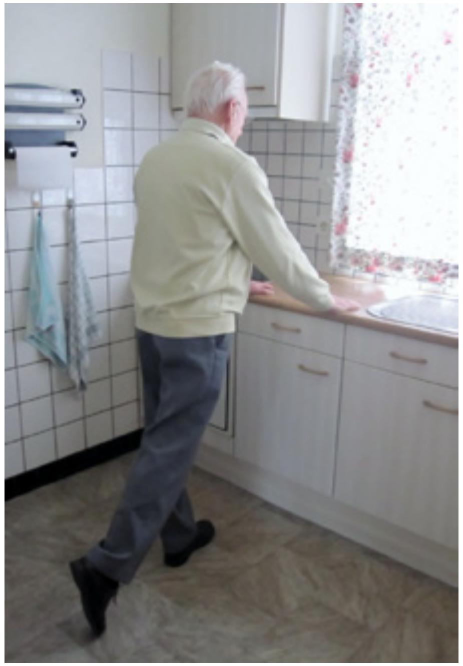

- Indien nodig, steun nemen
- Beweeg het rechterbeen naar achteren en raak met uw tenen de voer
- Herhaal ieder been 10x

Tip:

Probeer ook uw been achterwaarts omhoog te heffen

33. Kniebuigingen

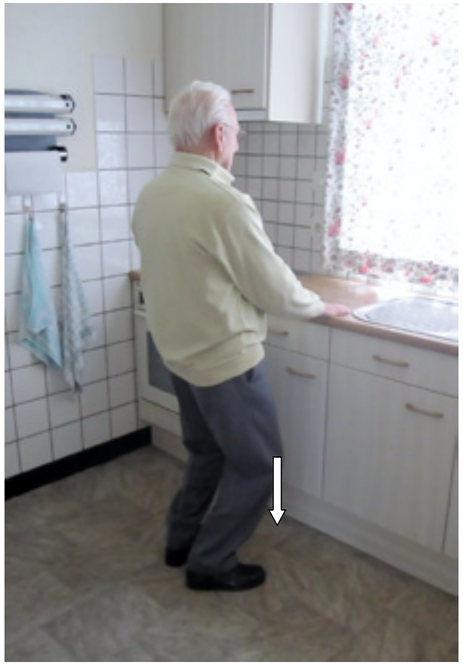

- Rechtop staan
- Indien nodig handen licht steunend
- Buig langzaam door uw knieën
- En ga weer staan
- Herhaal 10x

34. Opdrukken stoelleuning/zitting

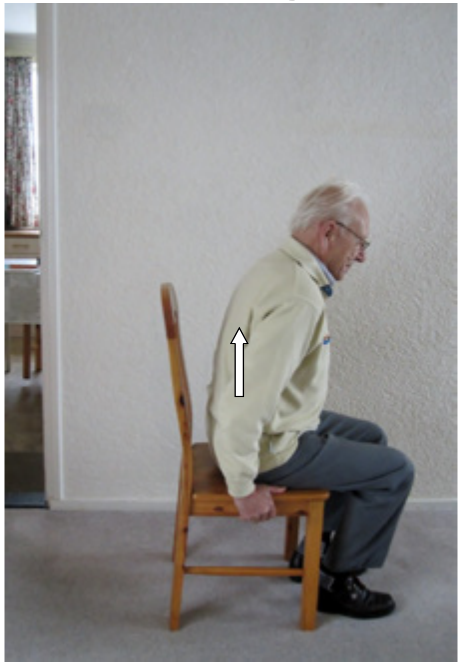

- Druk uw armen op door ze te strekken
- Gebruik hiervoor de stoelzitting of stoelleuning
- Neem hierbij zoveel mogelijk gewicht op uw armen

Tip:

Probeer deze oefening op te bouwen naar 5 tot 10x

C

D

35. Functionele krachtoefening beenspieren

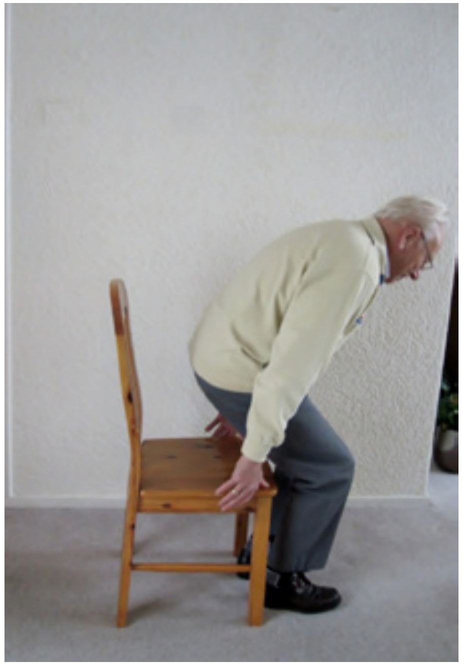

- Sta op
- Ga vervolgens langzaam zitten
- Herhaal 10x

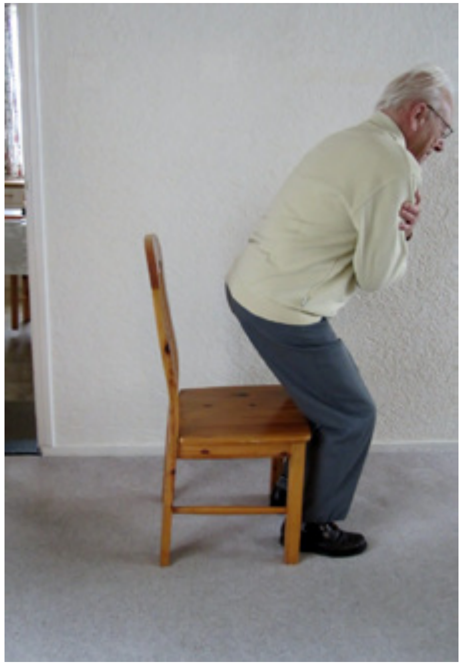

- Probeer deze oefening ook zonder steun van handen.
- Herhaal 1 tot 10x

Tip voor profiel D:

Probeer afhankelijk van uw fysieke conditie deze oefening op te bouwen en in series toe te passen. Bijvoorbeeld 3 series van 10 tot 15x met tussen de series een korte pauze.

36. Bruggetje

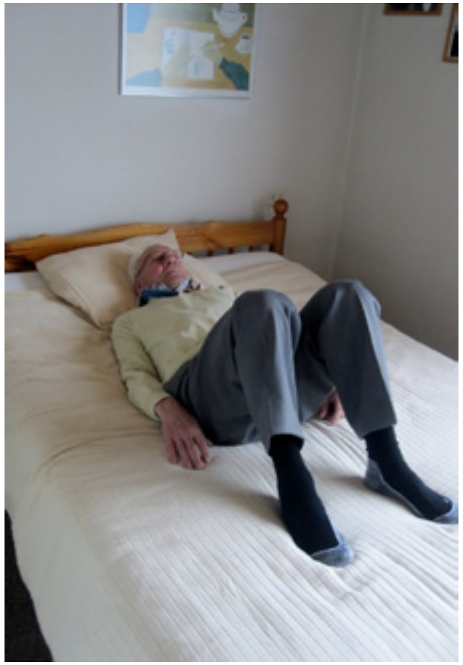

- Plaats uw voeten plat op het bed
- Breng uw hakken zo ver mogelijk naar uw billen

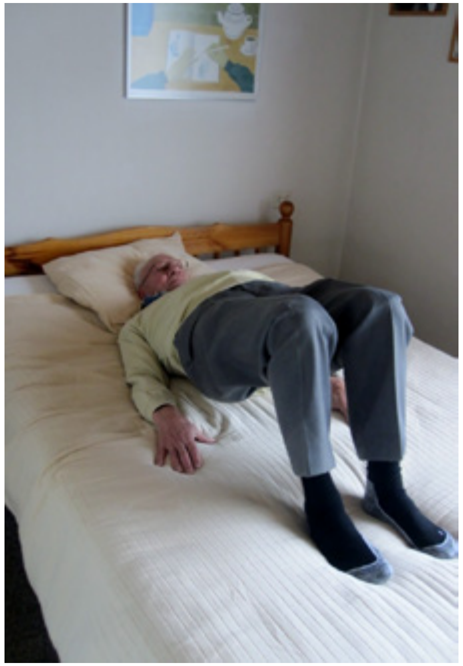

- Til uw billen op (bruggetje)
- Leg de billen weer terug op het bed

Tip:

Probeer de herhalingen op te bouwen naar 10x

37. Zijlig been

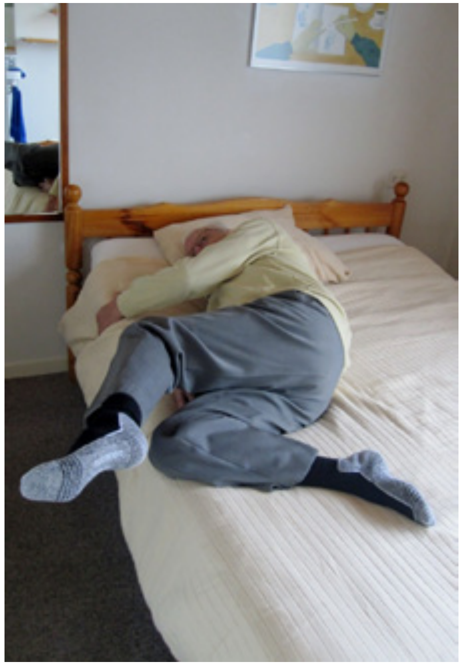

- Zijlig benen gebogen
- Uw bovenste been een stukje optillen
- En omlaag brengen
- Herhaal 1x en wissel van zij
- Bouw deze oefening voor beide kanten op naar 10x

38. Zijlig been heffen en uitstrekken

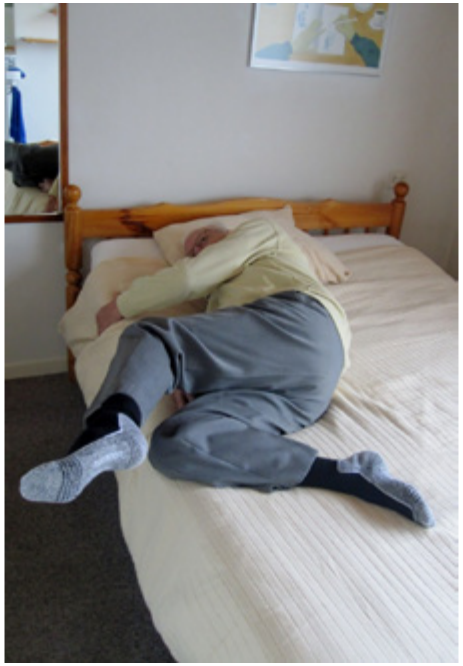

- Zijlig benen gebogen
- Uw bovenste been een stukje optillen

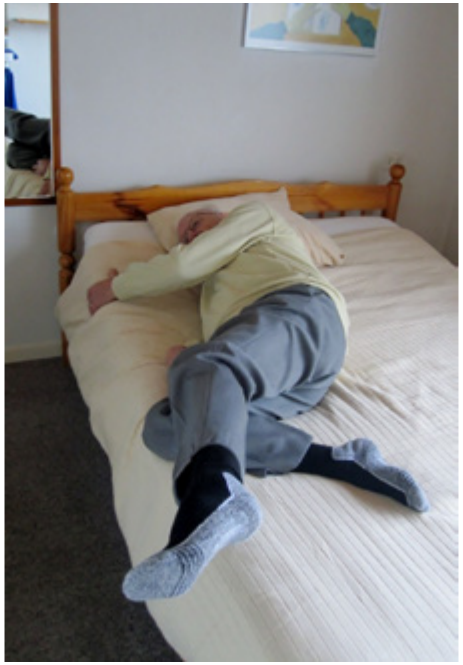

- Strek uw knie en probeer het been omhoog te houden
- Herhaal 1x en wissel van zij
- Bouw deze oefening voor beide kanten op naar 10x

39. Tenenstand

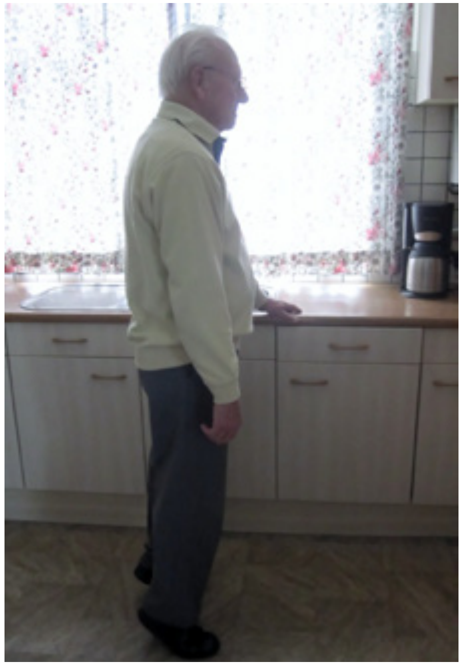

- Ga op uw tenen staan
- Neem steun indien nodig
- Herhaal 5 tot 10x

C

D

40. Hakkenstand

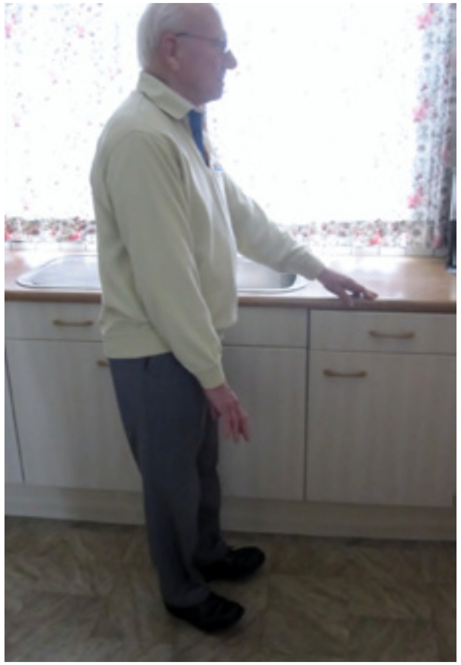

- Ga op uw hakken staan
- Neem steun indien nodig
- Herhaal 5 tot 10x

Tip:
Heeft u meer behoefte aan steun, steun dan met twee handen op het aanrecht

41. Voeten voor elkaar plaatsen

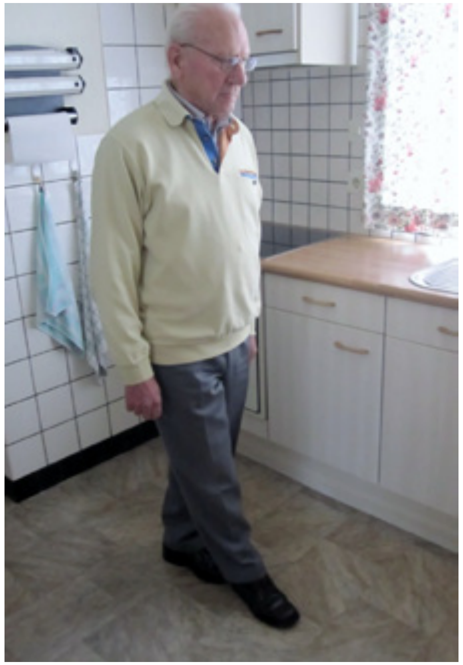

- Neem steun indien nodig
- Blijf 10 tot 30 sec. staan
- Wissel van voet

Tip:
Probeer ook 'voetje voor voetje' te lopen. Neem steun indien nodig.

42. Op één been staan

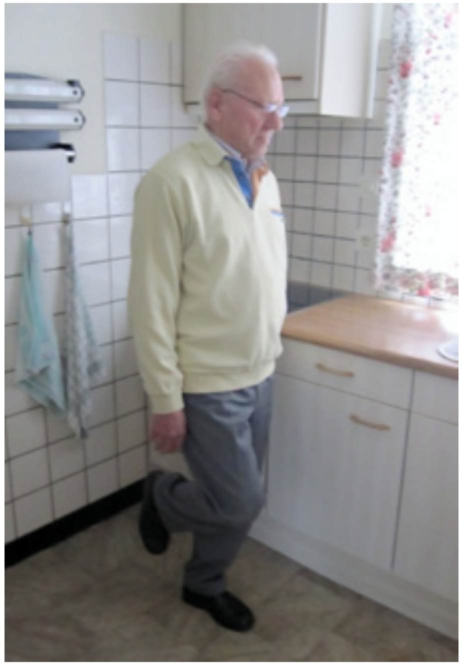

- Neem steun indien nodig
- Probeer 1 tot 10 sec. te blijven staan
- Herhaal 1 tot 5x voor beide benen

43. Voeten plaatsen op traptrede

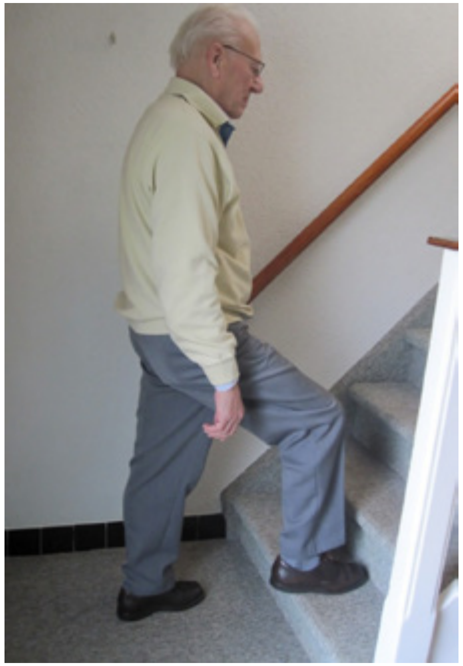

- Plaats de voeten afwisselend op de onderste traptrede
- Herhaal 5 tot 10x voor beide voeten
- Probeer deze oefening ook zonder steun aan trapleuning

C

D

44. Voor– en achterwaarts over voorwerp heen stappen

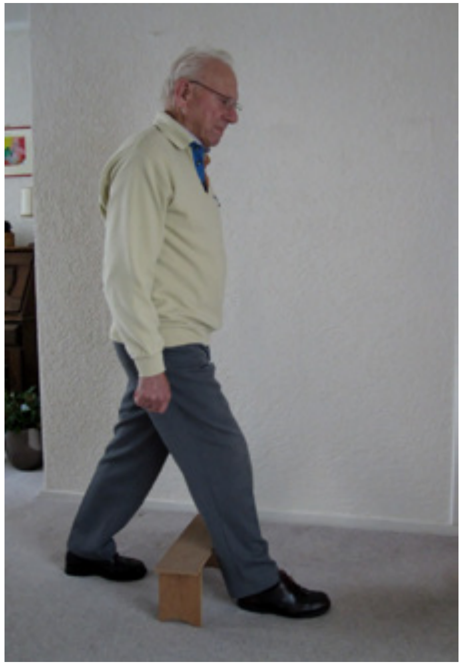

Tip:

Neem steun indien nodig door deze oefening in de keuken bij het aanrecht te doen

• Loop voor- en achterwaarts over voorwerp heen

• Herhaal 1 tot 10x

45. Voeten omlaag plaatsen en achterwaarts omhoog

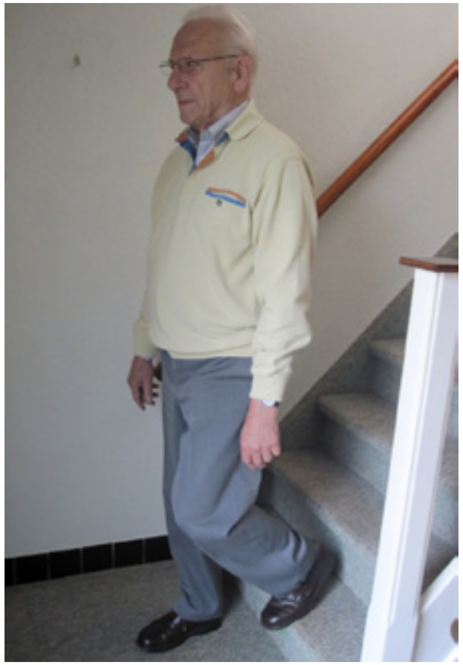

• Ga met twee voeten op de onderste traptrede staan

• Plaats afwisselend een voet op de grond en achterwaarts terug op de onderste traptrede

• Herhaal 5 tot 10x voor beide voeten

• Indien nodig, steun nemen aan trapleuning

46. Zijwaarts op- en afstappen

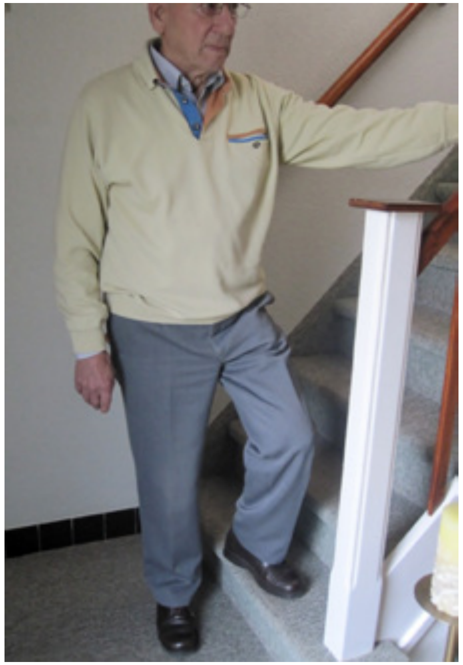

• Plaats uw linkervoet op de eerste traptrede

• Raak met uw rechtervoet afwisselend de eerste traptrede en de vloer

• Herhaal 5 tot 10x, ook voor de andere kant

• Indien nodig, steun nemen aan trapleuning

47. Bruggetje met één been

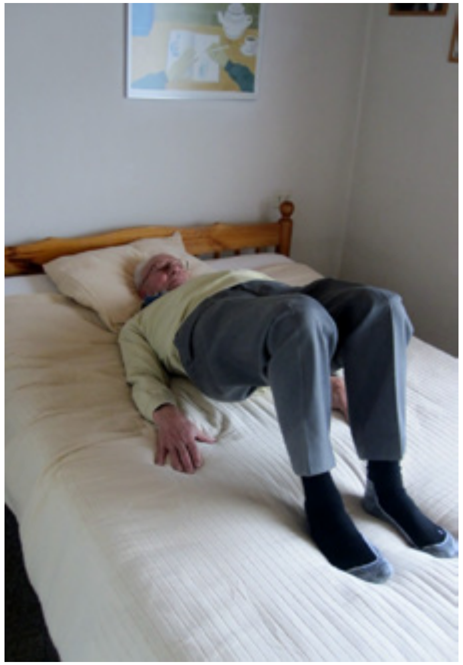

• Plaats uw voeten plat op het bed

• Breng uw hakken zo ver mogelijk naar uw billen

• Til uw billen op (bruggetje)

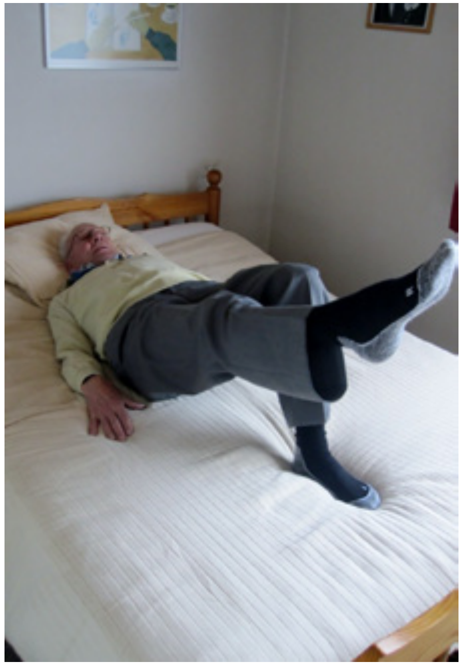

• Strek één been uit

• Laat uw billen omhoog

• Plaats het been terug en strek het andere been uit

Tip:

Deze oefening bevordert de spierkracht in uw bil en beenspieren. Probeer de herhalingen op te bouwen naar 10x.

D

48. Verbeteren van conditie d.m.v. wandelprogramma

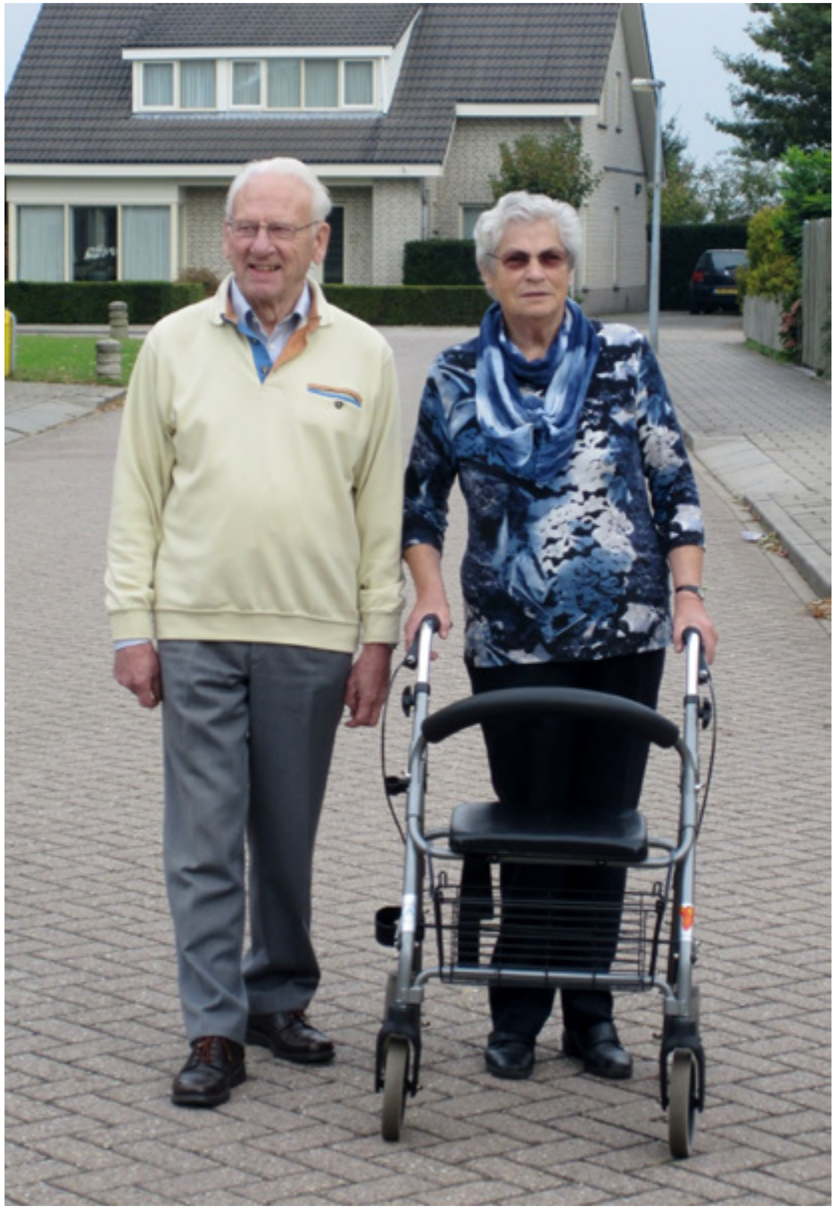

- Ga minimaal 2x per week 30 minuten wandelen
- U kunt indien dit nodig is hierbij een stok, rollator of bijvoorbeeld nordic walking stokken gebruiken
- Het kan zijn dat 30 minuten wandelen nog niet mogelijk voor u is. Stel dan een haalbaar doel, bijvoorbeeld lopen naar de winkel om de hoek en terug
- Ook meerdere keren per dag een stukje wandelen van bijvoorbeeld 10 minuten is een optie
- Probeer de afstand uit te breiden en te streven naar 30 minuten aan één stuk lopen

C

D
